# Intrathecal nivolumab in metastatic solid tumors with leptomeningeal disease: dose escalation part of the multicenter IT-PD1/NOA-26 phase 1 trial

**DOI:** 10.1038/s43018-026-01185-4

**Published:** 2026-06-04

**Authors:** Ghazaleh Tabatabai, Isabel Ramirez, Beatrix Welte, Paula Bombach, Hannes Becker, Denise Bernhardt, Regine Mayer-Steinacker, Friedegund Meier, Nicolas Neidert, Roland Roelz, Iris Mildenberger, Lukas Bunse, Michael Platten, Ulrich Herrlinger, Uwe M. Martens, Ulrike Ernemann, Manuela Neumann, Marcos Tatagiba, Lina Maria Serna-Higuita, Peter Martus, Mirjam Renovanz

**Affiliations:** 1 Department of Neurology & Interdisciplinary Neuro-Oncology, Hertie Institute for Clinical Brain Research, University Hospital Tübingen, Eberhard Karls University Tübingen, Tübingen, Germany; 2 Center for Neuro-Oncology, Comprehensive Cancer Center, University Hospital Tübingen, Eberhard Karls University Tübingen, Tübingen, Germany; 3 Cluster of Excellence (EXC 2180) ‘Image-Guided and Functionally Instructed Tumor Therapies’, Eberhard Karls University of Tübingen, Tübingen, Germany; 4 German Consortium for Translational Cancer Research (DKTK), DKFZ Partner site Tübingen, Tübingen, Germany; 5 Center for Clinical Trials, University Hospital Tübingen, Eberhard Karls University Tübingen, Tübingen, Germany; 6 Department of Neurosurgery, University Hospital Tübingen, Eberhard Karls University Tübingen, Tübingen, Germany; 7 Department of Radiation Oncology, University Hospital of the Technical University Munich, Munich, Germany; 8 Department of Medical Oncology and Hematology, University Hospital Ulm, Ulm, Germany; 9 Department of Dermatology, Faculty of Medicine and University Hospital Carl Gustav Carus, Technische Universität Dresden, Dresden, Germany; 10 National Center for Tumor Diseases (NCT), NCT/UCC Dresden, a partnership between DKFZ, Faculty of Medicine and University Hospital Carl Gustav Carus, TUD Dresden University of Technology, and Helmholtz-Zentrum Dresden-Rossendorf (HZDR), Dresden, Germany; 11 Department of Neurosurgery, University Hospital Freiburg, Breisgau, Germany; 12 Department of Neurology, Medical Faculty Mannheim, MCTN, University of Heidelberg, Mannheim, Germany; 13 DKTK (German Consortium for Translational Cancer Research) Clinical Cooperation Unit (CCU) Neuroimmunology and Brain Tumor Immunology, German Cancer Research Center (DKFZ), Heidelberg, Germany; 14 Department of Neurooncology, Center for Neurology, University Hospital Bonn, Bonn, Germany; 15 Department of Medical Oncology and Hematology, SLK-Clinics Heilbronn GmbH, Heilbronn, Germany; 16 Department of Neuroradiology, University Hospital Tübingen, Eberhard Karls University Tübingen, Tübingen, Germany; 17 Department of Neuropathology, University Hospital Tübingen, Eberhard Karls University Tübingen, Tübingen, Germany; 18 Department of Clinical Epidemiology and Applied Biostatistics, University Hospital Tübingen, Tübingen, Germany

**Keywords:** Cancer, Clinical trials

## Abstract

Leptomeningeal metastatic disease (LMD) of solid tumors represents a cancer stage with high unmet therapeutic need. Here we report results from the dose escalation part of a multicenter phase 1 trial investigating intraventricular nivolumab, now continuing in the expansion part. Eligible participants had LMD from tumors with an approval for intraveneous PD1/PDL1 therapy or high tumor mutational burden. The primary endpoint was safety across four dose levels (20, 30, 40 and 50 mg) with each cohort reviewed by an independent data safety monitoring board before escalation. The secondary endpoint was overall survival. Exploratory endpoints included participant-reported outcome measures. Of 30 enrolled participants, 24 received at least one dose (intention-to-treat population) and 18 completed predefined safety evaluations (per-protocol population). One dose-limiting toxicity occurred at 40 mg. The primary endpoint was met and the recommended fixed dose for the ongoing expansion part is 50 mg. Median overall survival (OS) was 6.6 months. The 6-month, 12-month and 18-month OS rates were 55.0% (95% confidence interval (CI): 37.5–80.6%), 33.3% (95% CI: 17.8–62.4%) and 16.7% (95% CI 6.03–46.1%), respectively. Participant-reported quality of life remained stable. Intraventricular nivolumab has demonstrated safety and feasibility (ClinicalTrials.gov: NCT05112549).

## Abstract (web summary)

Tabatabai et al. evaluated the safety of intrathecal administration of nivolumab in participants with leptomeningeal disease in the IT-PD1/NOA-26 phase 1 clinical trial.

## Main

Metastatic involvement of the central nervous system (CNS) by solid tumors can manifest as parenchymal brain metastases or as leptomeningeal disease (LMD). LMD, also known as leptomeningeal carcinomatosis, is a particularly aggressive and clinically challenging form of CNS dissemination. It occurs in approximately 5–10% of persons with solid tumors, most commonly arising from lung cancer, breast cancer or melanoma. The incidence of LMD is rising—likely because of improved systemic disease control and longer survival times, which allow for sanctuary site progression within the CNS. LMD remains associated with a dismal prognosis, with median OS ranging from 4 to 8 weeks following diagnosis^1–4^.

Current consensus guidelines^5–7^ define two major subtypes: type I LMD, characterized by confirmed malignant cells in cerebrospinal fluid (CSF) cytology or positive biopsy; type II LMD, based on characteristic clinical signs (for example, cranial neuropathies, radiculopathies and encephalopathy) and/or magnetic resonance imaging (MRI) findings (leptomeningeal contrast enhancement), in the absence of cytological confirmation^8,9^. Furthermore, the clinical utility of liquid biopsies is under continuous investigations^10^ and circulating tumor DNA in CSF has been included as supportive and supplemental diagnostic tools in the recent National Comprehensive Cancer Network (NCCN) guideline.

This reflects both the biological complexity of LMD and the clinical challenges posed by these individuals, who are often heavily pretreated and present with rapidly progressive neurological decline. Current treatment recommendations—such as those provided by the NCCN—stratify individuals into ‘good-risk’ and ‘poor-risk’ groups on the basis of performance status, systemic disease control and neurological burden. Mainstay therapies include radiotherapy, intrathecal chemotherapy (for example, methotrexate and cytarabine), systemic therapies that cross the blood–brain barrier and best supportive care^11,12^. A few prospective clinical trials have been conducted in this population including field and proton craniospinal irradiation^13^ and intrathecal therapy (IT) options with liposomal cytarabine, pemetrexed, patritumab deruxtecan and trastuzumab^14–18^.

Recent efforts have investigated the role of immune checkpoint inhibitors (ICIs) in LMD and shown potential activity of these agents, despite the unique immunological and pharmacokinetic challenges within the leptomeningeal compartment. Brastianos et al. conducted a single-arm, open-label phase 2 trial of intravenous pembrolizumab in participants with LMD from solid tumors, demonstrating preliminary activity and safety^19^. Prakadan et al.^20^ characterized immune responses in the leptomeningeal tumor microenvironment during intravenous ICI therapy, identifying correlates of response. Naidoo et al. treated LMD with intravenous pembrolizumab, showing clinical benefit in selected participants^19,21,22^. Glitza et al.^23^ recently reported on the IT administration route of nivolumab in melanoma-associated LMD.

Here, we report results of the dose escalation phase of the investigator-initiated phase 1 clinical trial IT-PD1/NOA-26, evaluating intrathecal administration of nivolumab in participants with LMD from various solid tumors, including nonmelanoma entities. Importantly, our trial systematically evaluates neurocognitive function, participant-reported psychological burden and health-related quality of life, critical endpoints that are highly relevant to participants’ daily function and care planning. Our findings offer insights into the feasibility of localized immune modulation in LMD and provide a rationale for further exploration of compartment-specific immunotherapy strategies.

## Results

### Clinical trial design and trial profile

We designed the IT-PD1/NOA-26 investigator-initiated phase 1 trial of intrathecal nivolumab in participants with LMD from solid tumors with a registration for intravenous medication with PD1 or PDL1 antibody in Europe. In a first amendment, we further included participants with a high tumor mutational burden (>10 mutations per Mb) (Methods and Supplementary Information).

**Fig. 1:**
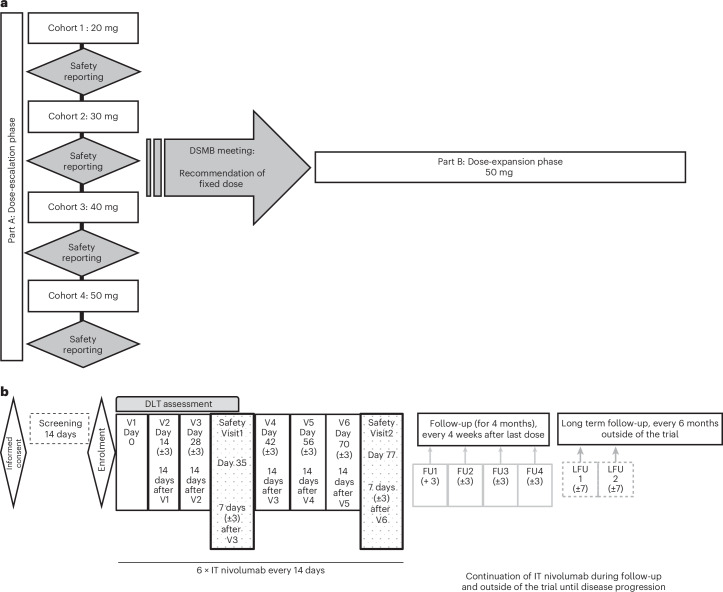
Trial design. **a**, This prospective, open-label, multicenter phase I trial included a 3 + 3 dose-escalation phase with four fixed-dose cohorts (20–50 mg) and an expansion cohort. Participants in part A were treated in a staggered manner, with DSMB safety reviews before each dose escalation. After cohort 4, the DSMB selected the dose for part B. Details are provided in the NOA-26 study protocol. **b**, After consent, eligibility was assessed during screening. Intrathecal nivolumab was administered at IMP visits 1–6 every 14 days (±3 days). Safety visits occurred 7 days after visit 3 (V3) and V6. Follow-ups (FUs) were performed every 4 weeks. Participants with stable disease continued treatment until progression. After FU4, participants were followed every 6 months for 1 year.

This phase 1 trial comprises two parts: an intrathecal nivolumab dose escalation phase (part A, 3 + 3 design) and a dose expansion cohort (part B) with a fixed recommended dose (Fig. 1a). For part A, we used four dose levels: 20, 30, 40 and 50 mg. Given the 3 + 3 design for part A and four cohorts, the initial enrollment plan was to include 12–24 eligible participants for safety assessment. The primary endpoint was safety, dose-limiting toxicity (DLT) and the definition of the maximum tolerable dose of IT nivolumab for Part B. The secondary endpoint was OS and exploratory endpoints included participant-reported outcome assessments (Methods).

Eligible participants were ≥18 years of age, provided written informed consent and had a good-risk profile according to NCCN guidelines (version 1.2021). Enrollment required confirmation by a tumor board of clinical indication for intrathecal (IT) therapy, potential need for systemic treatment and feasibility of study participation. Participants were required to have a Karnofsky performance score (KPS) > 50% and a confirmed diagnosis of LMD according to positive CSF cytology and/or MRI findings^8^. Thorough CSF evaluation, including exclusion of differential diagnoses, was mandatory. Repeated lumbar punctures were recommended if initial cytology was negative. Participants were required to be able to receive IT nivolumab through an intraventricular catheter. Female participants of childbearing potential and male participants with female partners of childbearing potential were required to use highly effective contraception during treatment and for 150 days thereafter. Prior radiation therapy of the CNS, if performed, had to be completed ≥2 weeks before enrollment and ≥4 weeks before the first IT investigational medicinal product (IMP) dose (Methods).

Study treatment consisted of six IT applications of nivolumab every 2 weeks followed by follow-up visits and long-term follow-up visits outside the trial (Fig. 1b). Participants who received at least one intrathecal nivolumab application were defined as the intention-to-treat (ITT) population in the study protocol. Dropouts within the ITT population were defined if a participant after enrollment and before safety visit 1 suffered from tumor progression, death, withdrawal of informed consent or any other reasons, but without DLT. As predefined in the protocol, dropouts before safety visit 1 were replaced. The per-protocol (PP) population included those participants of the ITT who participated in safety visit 1 and were eligible for safety and DLT assessment by the data safety monitoring board (DSMB).

**Fig. 2:**
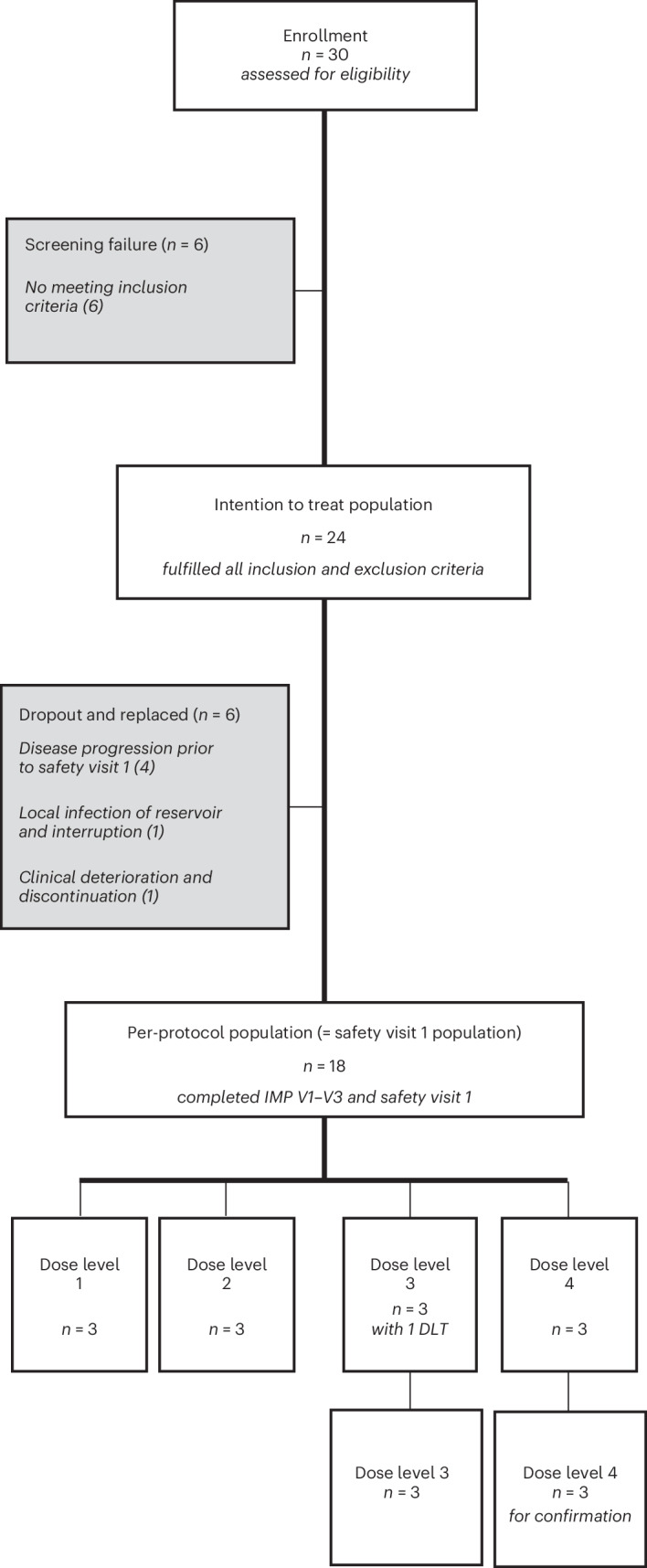
Trial profile. Profile of participant enrollment. The CONSORT checklist based on current guidelines^36^ is included in the Supplementary information.

From December 2021 through the data cutoff date April 22, 2025, a total of 30 participants consented; 24 participants fulfilled all inclusion and exclusion criteria and received at least one dose of IMP (ITT) and 18 participants comprised the predefined PP population (safety visit 1 population; Fig. 2). We had six dropouts and one DLT in dose level 3 (40 mg) (individual participant summaries from NOA-26 in Supplementary Information). The DSMB recommended three additional participants in the last dose level, in the absence of a DLT, to confirm the safety of 50 mg of nivolumab.

The primary analysis population was the PP population (Fig. 2). Baseline characteristics, OS and participant-reported outcomes were analyzed in the ITT population. Participant characteristics at baseline are outlined in Table 1. In brief, the median age was 50.5 years, the median KPS was 75%, most of the participants were female (*n* = 18) and primary tumor entities were melanoma, non-small cell lung cancer (NSCLC) and breast cancer (Table 1).

**Table 1:** Participant characteristics at baseline (ITT population, *n* = 24)

Variable	*n* (%)
Age (median, min–max) (IQR)	50.5 (18–63) (38–58)
KPS (median, range) (IQR)	75 (50–90) (70–80)
	n (%)
Gender	
• Male	6 (25)
• Female	18 (75)
Primary tumor diagnosis	
• Melanoma i) *BRAF*^V600E^ mutant ii) *BRAF*^V600E^ mutant, *NRAS* wild type iii) *BRAF*^V600K^ mutant, *NRAS* wild type iv) *BRAF*^V600E^ wild type, *NRAS* wild type • Lung cancer i) NSCLC PDL1 ≥ 50%, EML4–ALK fusion present EML4–ALK fusion present PDL1 ≤ 50%, EML4–ALK fusion absent PDL1 ≤ 50% EML4–ALK fusion absent ii) SCLC • Breast Triple-negative	13/24 (54) 6/13 (46) 3/13 (23) 1/13 (8) 3/13 (23) 7/24 (29) 5/7 (71) 1/5 (20) 1/5 (20) 1/5 (20) 1/5 (20) 1/5 (20) 2/7 (29) 4/24 (17) 4 (100)
MRI brain consistent with LMD	22/24 (92)
MRI spine consistent with LMD	13/24 (54)
MRI brain plus MRI spine consistent with LMD	11/24 (46)
CSF consistent with LMD	8/24 (33)
Parenchymal brain metastases in addition to LMD	14/24 (58)
Spinal metastases in addition to LMD	3/24 (13)
Prior whole-brain radiation therapy	12/24 (50)
Prior stereotactic radiation to CNS	14/24 (58)
Prior intravenous checkpoint inhibitors	13/24 (54)
Glucocorticoid dosage, dexamethasone equivalent at enrollment	
• None • 0.5 mg • 0.67 mg • 0.86 mg • 1.0 mg • 1.5 mg	14/24 (58) 1/24 (4) 1/24 (4) 1/24 (4) 1/24 (4) 6/24 (25)

Source data

### Safety assessments during intrathecal nivolumab dose escalation

Participants of the PP population received at least three intraventricular dosages and safety data at safety visit 1 were defining for the assessment by the DSMB. The treatment was technically feasible in all cases without intraparticipant adjustment. Treatment-related adverse events (TRAEs) that were at least possibly related to intrathecal administration of nivolumab by the investigator were generally manageable and predominantly low-grade. The most frequent TRAEs included nausea (*n* = 9, spanning grades 1 to 3), headache (*n* = 5, primarily grade 1), tingling in the feet and/or face (*n* = 6, all grade 1) and fever (*n* = 3, grade 1). Several participants experienced neurologic symptoms such as dizziness (*n* = 5, mostly grade 1 with one grade 3 event), double vision (n = 1, grade 1) and dysphasia (n = 3, all grade 3) (Table 2). Other low-grade events included fatigue, decreased appetite and intermittent meningism. These findings suggest an acceptable safety profile for IT nivolumab, with close monitoring warranted for neurological and hepatic adverse effects. One DLT occurred in cohort 3 participant NOA-26-013. Participant 01-013 received three IT administrations of nivolumab and completed safety visit 1. The participant developed a urinary tract infection leading to sepsis, followed by hepatic failure (common toxicity criteria (CTC) grade 4). This serious AE was rated as DLT and reported in an ad hoc DSMB meeting. The DSMB assessed the serious AE as likely related to IT nivolumab, potentially exacerbated by concurrent BRAF and MEK inhibition (individual participant summaries from NOA-26 in Supplementary Information).

**Table 2:** TRAEs until safety visit 1

AE	CTC grade 1	CTC grade 2	CTC grade 3	CTC grade 4
Chills	1			
Decreased AP		1		
Decreased bilirubin		1		
Diarrhea		1		
Dizziness	4		1	
Double vision	1			
Dysphasia			3	
Fatigue		1		
Fever	3			
Gait uncertainty	1			
Headache	4	1		
Hepatic failure				1
Involuntary movement eyelid	1			
Involuntary movements both legs	1			
Meningism intermittent		1		
Muscle twitching	1			
Nausea	3	5	1	
Numbness in the lips and right side of the face	1			
Paresis trochlearis nerve		2		
Sepsis				1
Tingling in feet and/or face	6			
Vomiting	1	3		
Weight loss	1			

### AEs related to Ommaya reservoir

According to the inclusion criteria of the NOA-26 trial, participants were required to have a clinical indication for IT and an Ommaya reservoir in place before enrollment and treatment initiation. Consequently, implantation of the Ommaya reservoir was not considered a trial-specific procedure. Nonetheless, all AEs were assessed for a potential association with the reservoir. Three participants experienced serious AEs before IMP visit 1 administration that were attributed to the Ommaya reservoir. In participants NOA-26-005 and NOA-26-024, revision surgery was required because of device malfunction (Table 2); both events were graded as CTC grade 3. In addition, participant NOA-26-006 developed headache following Ommaya reservoir implantation, classified as a CTC grade 3 event and managed with ibuprofen and metamizole (Table 2).

### Clinical outcome parameters and participant-reported quality of life

**Fig. 3:**
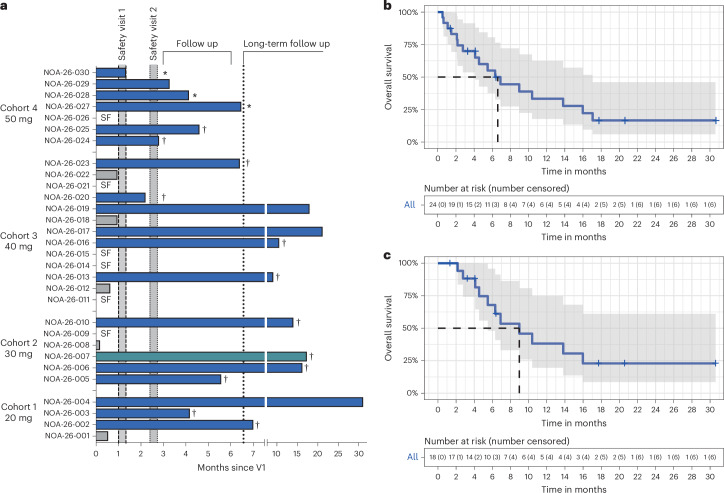
Clinical outcome. **a**, Bars represent 30 enrolled participants including screening failures (SFs). Participant NOA-26-007 (green) discontinued after IMP V2 because of infection but resumed treatment during follow-up; the green bar denotes OS. Blue bars represent OS in participants of PP populations. Gray bars represent dropouts. Participants with asterisks (*) are still in the trial. A cross (+) indicates death. **b**, OS of ITT population (*n* = 24). **c**, OS of PP population (*n* = 18). Shaded areas represent 95% CIs; vertical marks (|) indicate censored subjects. Dashed lines denote the median survival time. ‘Number at risk’ indicates number of participants alive and under observation; values in parentheses represent the cumulative number of censored participants. Source data

As of the data cutoff on April 22, 2025, seven participants were alive and three participants of the dose level 4 cohort were still in the trial: NOA-26-027 and NOA-26-028 who were in follow-up and NOA-26-030 who completed safety visit 1 and was still on study treatment (Fig. 3a). Three participants continued with IT nivolumab treatment outside the trial in the long-term follow-up: NOA-026-004 from dose level 1 (20 mg) and NOA-26-017 and NOA-26-019 from dose level 3 (40 mg). These participants were alive 31, 21 and 20 months, respectively, after the first IMP dose (Fig. 3a). While safety and toxicity were the primary endpoints, OS was a preplanned secondary endpoint.

**Fig. 4:**
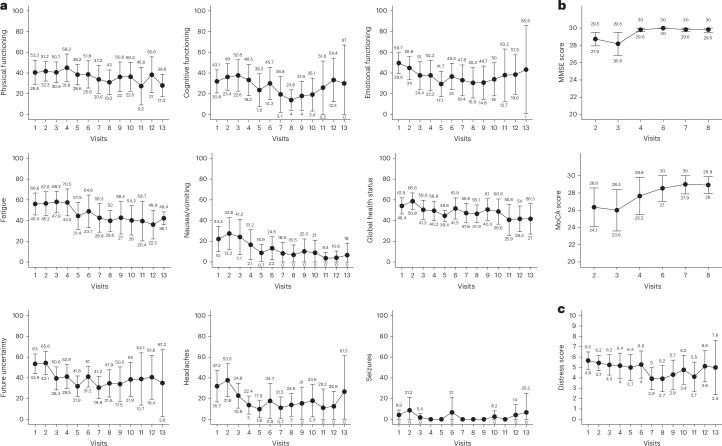
Participant-reported quality-of-life and neurocognitive assessments. **a**, Selected scales from European Organization for Research and Treatment of Cancer QLQ-C30 and QLQ-BN20 (scores 0–100; higher scores indicate better functioning or greater symptom burden, respectively) (participants, *n* = 24). **b**, Neurocognitive results from Mini Mental Status Examination and Montreal Cognitive Assessment (scores 0–30; higher scores indicate better cognitive function) (participants, *n* = 24). **c**, Distress scores (range 0–10; higher scores indicate greater psychological distress) (participants, *n* = 23). Points represent mean values of the items; error bars indicate 95% CIs. Source data

**Extended Data Fig. 1:**
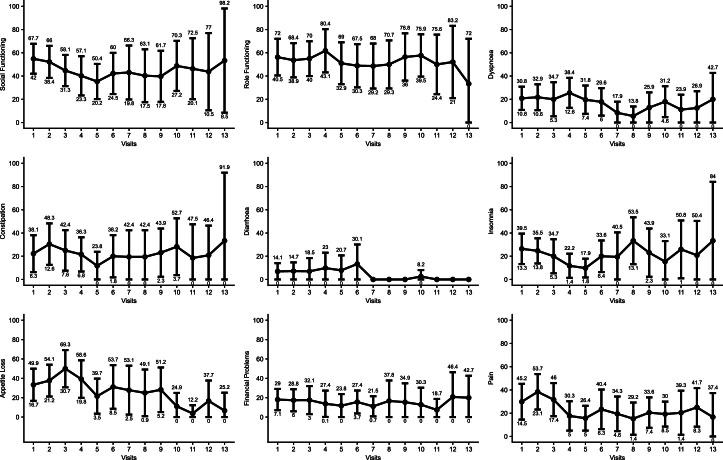
Additional functioning and symptom scales and single items from the EORTC QLQ-C30. Scores 0–100; higher scores indicate better functioning or greater symptom burden, respectively), 24 patients included (n = 24). Points represent mean values of the items, and error bars indicate 95% confidence intervals (CI). Number of patients per visit as indicated in Supplementary Table 1. Source data

**Extended Data Fig. 2:**
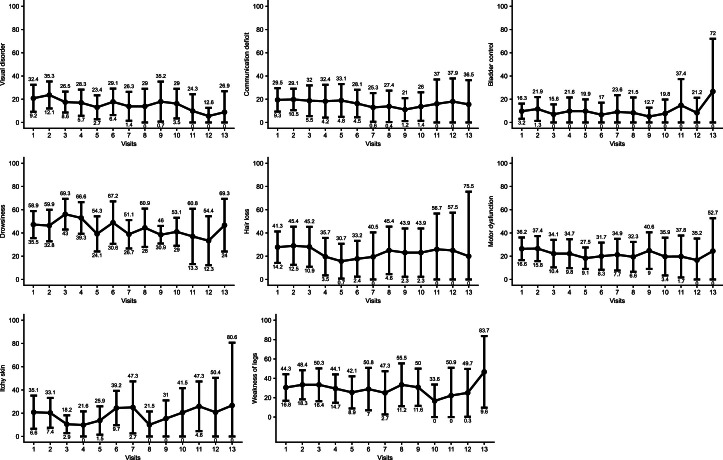
Additional functioning and symptom scales and single items from the EORTC QLQ-BN20. Scores 0–100; higher scores indicate better functioning or greater symptom burden, respectively), 24 patients included (n = 24). Points represent mean values of the items, and error bars indicate 95% confidence intervals (CI). Number of patients per visit as indicated in Supplementary Table 1. Source data

The median OS in the ITT population was 6.6 months (95% confidence interval (CI): 4.1–17.1 months) with a 6-month OS rate of 55.0% (95% CI: 37.5–80.6%), 12-month OS rate of 33.3% (95% CI: 17.8–62.4%) and 18-month OS rate of 16.7% (95% CI: 6.03–46.1%) (Fig. 3b). In the PP population who completed safety visit 1, the median OS was 9.0 months (95% CI: 5.4 months–not attained) with a 6-month OS rate of 67.9% (95% CI: 48.1–95.8%), 12-month OS rate of 38.2% (95% CI: 19.5–74.6%) and 18-month OS rate of 22.9% (95% CI: 8.6–61.1%) (Fig. 3c). All participants who were deceased at the time of analysis demonstrated radiographic or clinical progression before death. During the study, we systematically conducted neurocognitive bedside tests and participant-reported quality-of-life assessments and distress. These parameters remained stable (Fig. 4 and Extended Data Figs. 1 and 2).

## Discussion

LMD represents a devastating manifestation of cancer, characterized by diffuse dissemination of malignant cells in the leptomeninges and marked by a dismal prognosis and limited therapeutic options. In the NOA-26 investigator-initiated phase 1 trial, we demonstrate that IT administration of nivolumab in participants with LMD from solid tumors is feasible, generally well tolerated and associated with signals of clinical activity, as well as stable participant-reported quality of life and neurocognitive functioning.

The pathophysiology of LMD and its underlying biology and molecular mechanism are complex, involving a combination of impaired CSF flow dynamics, immunosuppressive microenvironments and distinct molecular alterations^24,25^. Among the existing therapeutic approaches, IT drug delivery remains one of the few options capable of achieving therapeutic drug concentrations within the CSF. IT administration has been traditionally used for conventional chemotherapeutic agents (for example, methotrexate and cytarabine). IT therapies have manageable adverse effects^26,27^, including reports from clinical trials using intrathecal applications^28–31^. ICI therapy, administered systemically, is a valid option^32,33^ and has been investigated in LMD of different solid tumors^19,21,34^.

In NOA-26, we investigated an IT delivery route. The safety profile in NOA-26 is consistent with a previous study of IT nivolumab in participants with melanoma and LMD^23^. In their study, they used 5, 10, 20 and 50 mg and did not observe any DLT.

We observed one DLT with 40 mg of nivolumab. In line with the 3 + 3 design, this result necessitated the enrollment of three additional participants at the same dose level before escalation to the next cohort (cohort 4). The final maximum tolerated dose (MTD) was established at 50 mg. In a sensitivity analysis, we found that the decision rules for dose escalation and the choice of the MTD were identical to those obtained from a more advanced Bayesian optimal interval design^35^.

In the LMD melanoma trial, a median OS of 4.9 months was reported as a secondary outcome parameter. In part A of NOA-26 and until the data cutoff of our analysis, we observed a median OS of 6.6 months (95% CI: 4.1–17.1 months) (Fig. 3b) across a study cohort of different solid tumors, demonstrating that the therapeutic application of IT-PD1 blockade may extend beyond melanoma. Furthermore, a secondary OS analysis in the PP population (Fig. 3c) revealed a median OS of 9.0 months (95% CI: 5.5 months–not attained). We view this as an encouraging signal at this early stage and enrollment for part B of the NOA-26 trial is currently ongoing. The final analysis will allow more comprehensive analyses of clinical outcome, including comparisons with previous studies and will certainly support the development of additional hypotheses for the next phase of clinical development. Importantly, our trial investigated systematically participant-reported outcome measures during intraventricular nivolumab treatment. These data show sustained neurological function and preserved participant-reported quality of life over time, highlighting the functional relevance of this approach. Although participation in participant-reported outcomes and neurocognitive screening declined over time, at least half of the participants completed assessments up to visit 10 (Supplementary Table 1), supporting the validity of the results. Nevertheless, a potential bias must be considered, as participants with higher symptom burden might have been unable to complete the questionnaires.

Systemic PD1/PDL1 blockade and other systemic treatments were administered in parallel in the majority of participants in our trial, without signs of cumulative immune-related (ir)AEs. This aligns with emerging preclinical data suggesting that intrathecal checkpoint inhibition can synergize with systemic immunity through trafficking of activated T cells across CNS interfaces, although further mechanistic studies are warranted to confirm these effects in participants. The currently ongoing expansion part of NOA-26 will further provide insight into coadministration of 50 mg of nivolumab plus systemic anticancer treatments. While the heterogeneous tumor types included in NOA-26 limit the statistical power for subgroup analyses, it is remarkable that we observed long-term responses, even beyond 12 months (Fig. 3), in a disease that has a devastating prognosis. Nevertheless, this study had limitations. It was conducted at an early stage of clinical development (phase 1). In addition, the LMD study population was inherently selected according to predefined inclusion and exclusion criteria. These findings are, therefore, not generalizable to the broader population and warrant further investigations in larger prospective clinical trials.

Taken together, our findings demonstrate the feasibility and acceptable safety of IT nivolumab in patients with LMD from solid tumors. They also complement and expand prior studies by providing prospective, protocol-driven evidence in a more heterogeneous disease population. The encouraging signals of clinical benefit in some individuals combined with preserved neurocognitive function and quality of life justify further investigation in the ongoing expansion part of NOA-26 and at a later stage with distinct combination treatments, as well as in randomized control settings.

## Methods

### Regulatory status and study enrollment

This study was an authorized nonrandomized multicenter phase 1 investigator-initiated trial. The study design complied with all relevant regulations regarding the use of human study participants and was conducted in accordance with the criteria set by the Declaration of Helsinki. Participants were recruited at all clinical sites in Germany according to predefined inclusion and exclusion criteria. Eligible participants were identified by study investigators and enrolled following provision of informed consent. Because of the specific nature of the disease and its very high unmet therapeutic need, rarity and often unfavorable course, the study population was necessarily selected. Furthermore, this was a phase 1 trial. These factors limit generalizability to broader populations at this stage of clinical development.

The first submission to the institutional review board and to the Paul Ehrlich Institute (PEI) was in May 2021. The study received approval by the PEI in August 2021 and by the institutional review ethics board in September 2021. Recruitment was initiated on October 12, 2021, the first participant enrolled in December 2021 and part A was completed in April 2025.

The data cutoff of this report was 22 April 2025.

The first substantial amendment was submitted in July 2022 and approved. The study population was expanded by solid tumors with high tumor mutational burden. The second substantial amendment was submitted in September 2023 and included the following major changes: (1) an interruption of the IMP administration for up to 6 weeks because of related or unrelated AEs; (2) participant information with secondary use of biosamples; and (3) the current version of the SmPC.

The NOA-26 trial was transferred on the basis of Clinical Trials Regulation (CTR) in Europe and the Clinical Trials Information System (CTIS) transition was completed. NOA-26 has run under the CTR since May 24, 2024. The first substantial amendment after CTIS transition was implemented in November 2024. The current version of the NOA-26 Study protocol is version 5.1 (November 15, 2024) and is included in the Supplementary information.

Data collection and analysis were not performed blind to the conditions of the experiments.

### Study oversight

A copy of the clinical trial protocol and the CONSORT checklist (in compliance with CONSORT guidelines^36^) are available in the Supplementary Information. The investigator-initiated trial was designed by the Lead principal investigator and sponsor delegate and conducted in accordance with the provision of the declaration of Helsinki and Good Clinical Practice guidelines. It was approved by the institutional review board and the PEI.

The trial is registered on ClinicalTrials.gov (NCT05112549).

Part A was designed as the dose escalation phase of the trial with a 3 + 3 design, with four dose levels (20 mg, 30 mg, 40 mg and 50 mg), leading to a minimum of 12 and a maximum of 24 subjects.

After completion of safety visit 1 in each cohort without any DLT, the trial proceeded with enrollment into the next-higher-dose cohort. If one study participant developed a DLT at a specific dose, three additional subjects were enrolled into that same dose cohort. Development of DLTs in more than one of six participants in a specific dose cohort would suggest that the MTD was exceeded and further dose escalation was not allowed.

On each dose level, the exposure of participants followed a staggered approach by an interval of at least 1 week for the first three participants in each cohort.

The DSMB evaluated the safety reporting after each dose level. Furthermore, at the end of part A, the DSMB made the recommendation to determine the recommended fixe dose for part B.

For the ongoing part B (expansion phase), a maximum of 25 and a minimum of 20 subjects will be treated.

The primary objective was to assess the maximum tolerable dose and safety of IT nivolumab. The secondary endpoint was OS. Exploratory analyses included participant-reported outcome assessments.

DLT were defined as follows: (1) Any AE of CTCAE grade ≥4 related to the IMP and (2) all neurological AEs of CTCAE grades 2 and 3.

**Extended Data Fig. 3:**
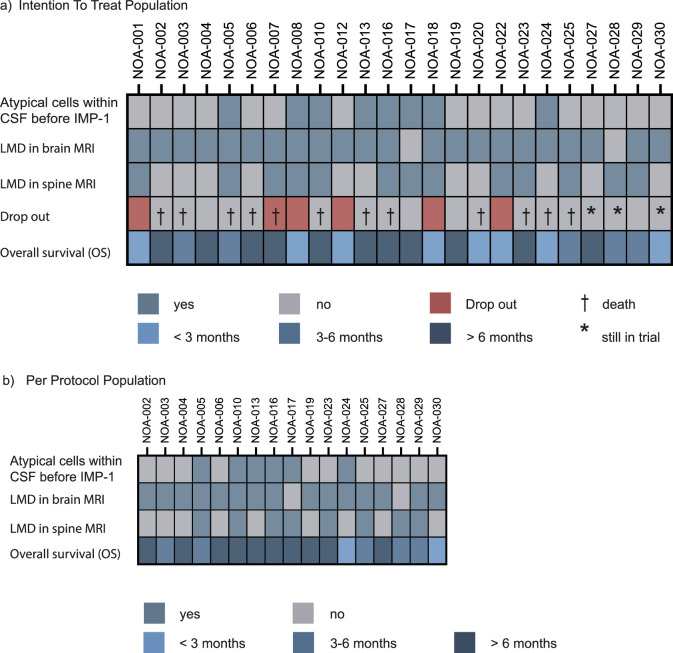
Neuroanatomical localization and CSF cytology. Neuroanatomical localization of leptomeningeal disease (LMD) was assessed by cerebral and spinal MRI. Corresponding CSF cytology is shown per patient. Individual overall survival is shown for each patient in the Intention To Treat Population (a) and in the Per Protocol Population (b). Abbreviations: CSF; Cerebrospinal Fluid, LMD; Leptomeningeal Disease. Source data

Extended Data Fig. 3 gives an overview on neuroanatomic LMD localization and CSF cytology per participant. Supplementary Table 2 summarizes systemic therapies before enrollment and during trial treatment. Supplementary Table 3 includes all TRAEs until safety visit 1.

The trial has been monitored by an independent DSMB who evaluated the study after each cohort and decided on proceeding to the next dose level.

Further information on research design is available in the Nature Portfolio Reporting Summary linked to this article.

### Intraventricular delivery route for intrathecal application of nivolumab

The IMP for this study was nivolumab, an anti-PD1 antibody. In NOA-26, each participant received six IT applications of nivolumab every 2 weeks with preplanned safety visits after three administrations. After safety visit 2, the participant entered the follow-up phase. Afterward, participants could continue with IT nivolumab until disease progression.

Intrathecal administration of nivolumab was performed exclusively through the ventricular route. Enrollment required a tumor board recommendation, as outlined in inclusion criterion 4. Fixed dosing was implemented without intraparticipant escalation or reduction; administered doses ranged between 20 mg and 50 mg. The MTD identified in part A, as determined by the DSMB, was carried forward into part B.

Concurrent systemic PD1/PDL1 inhibition was permitted on the basis of tumor board recommendation but had to be scheduled at least 24 h apart from intrathecal administration. Both systemic therapy and IT were withheld in the event of irAEs requiring discontinuation. Participants with prior systemic PD1/PDL1-related irAEs could be treated intrathecally if full recovery was documented.

### Dose escalation and definition of DLTs

For the 3 + 3 design, three subjects were initially enrolled into cohort 1 with a given dose of 20 mg. If safety visit 1 was completed and there was no DLT observed in any of these subjects, the trial proceeded to enroll three subjects into the next-higher-dose cohort 2 with a given dose of 30 mg. If there was no DLT observed in any of these subjects, the trial proceeded to enroll an additional three subjects into the next-higher-dose cohort 3 with a given dose of 40 mg. If there was no DLT observed in any of these subjects, the trial proceeded to enroll an additional three subjects into the final next-higher-dose cohort 4 with a given dose of 50 mg.

DLTs were defined in the protocol as (1) CTCAE grade ≥4 AEs related to the investigational drug or (2) CTCAE grade 2–3 neurological events attributable to the study drug that, according to the 2021 NCCN Guidelines on immune-related toxicity management, warranted permanent discontinuation of checkpoint inhibitors.

The DSMB reviewed all safety data after each cohort and approved the continuation to the next dose level. In the last dose level (as outlined in Fig. 2), three participants were enrolled without any DLT. To confirm this, three additional participants were enrolled per request of the DSMB. These additional three participants did not experience any DLTs and the safety signal was confirmed. As this was a phase 1 trial, no further replications were foreseen within the scope of this study. The currently ongoing expansion phase (part B) with a fixed dose of 50 mg will investigate and confirm safety of treatment with 50 mg of nivolumab IT.

### Statistical methods

The statistical analyses for the NOA-26 trial are outlined in the enclosed study protocol (Supplementary Information). Chapter 9.1.1 gives an overview of sample size and power calculations. This trial included a minimum of 32 evaluable participants (12 in part A, 20 in part B) and a maximum of 49 evaluable participants (24 in part A, 25 in part B). Between 12 and 24 participants were included in the dose-finding phase part A with four doses (20, 30, 40 and 50 mg) and 20 evaluable participants were included in the expansion phase using the MTD identified in the 3 + 3 phase. With 20 evaluable participants, it could be shown that the DLT was smaller than 33%, assuming a true DLT of maximal 7% (exact binomial test, type 1 error = 0.025 one-sided, power = 80%, H0: DLT = 33%, H1: DLT < 33%, assumed alternative: DLT ≤ 7%). The statistical analysis was descriptive. Dose levels were pooled because of the small number of subjects per level (*n* = 3 or *n* = 6). For continuous measurements, medians and interquartile ranges (IQRs) were used; for categorical variables, absolute frequencies and percentages were used. Censored data were presented using Swimmer plots, Kaplan–Meier plots and related parameters (median survival and survival after 6, 12 and 18 months including two-sided 95% CIs). Quality-of-life and cognitive scales were displayed using line graphs including two-sided 95% CIs. In these figures, for each time point, the number of participants under observation is given. These numbers are presented in an additional table. We used SPSS for Windows (IBM, release 29) and R (release 4.1.2)^37,38^. No data were excluded from the analyses. Participants who did not meet screening criteria or who withdrew during the trial are separately identified, with their status clearly defined in the corresponding tables and figures.

### Reporting summary

Further information on research design is available in the Nature Portfolio Reporting Summary linked to this article.

## Supplementary information

Supplementary Information Individual participant summaries.

Supplementary Information Supplementary Tables 1–3.

Reporting Summary

Supplementary Data Study protocol.

Supplementary Data CONSORT 2025 checklist.

## Source data

Source Data Table 1, Figs. 3 and 4 and Extended Data Figs. 1–3 Raw data and statistical source data.

## Data Availability

All data included in the paper were generated as part of the clinical trial and are subject to participant confidentiality. Thus, pseudonymized or deidentified clinical data can be made available per request. All requests will be reviewed by the Institutional Review Board of the University Hospital Tübingen and the Legal Department of the University Hospital Tübingen. Any requests should be directly addressed to the corresponding author (ghazaleh.tabatabai@med.uni-tuebingen.de). These requests will be processed within a timeframe of 3–6 months. Any participant-related data not included in this publication were generated as part of the NOA-26 clinical trial and are protected by participant confidentiality. All deidentified clinical data will be made available with the final reporting of the NOA-26 clinical trial. All participants are referred to in the manuscript with a code (NOA-26-XXX). This code is not the study identifier of these participants but a code that was used for this manuscript and this publication. The full study protocol is available in the Supplementary Information. The full set of clinical data (all AEs, all TRAEs and individual participant summaries) is available in the Supplementary Information. Source data are provided with this paper.
