# Supplementary material for: Intrathecal nivolumab in metastatic solid tumors with leptomeningeal disease: dose escalation part of the multicenter IT-PD1/NOA-26 phase 1 trial
Source: Nat Cancer. 2026 Jun 4;7(7):1094–103. doi: 10.1038/s43018-026-01185-4 (PMC13400297; doi:10.1038/s43018-026-01185-4)
Supplement: Supplementary file 1 — Individual participant summaries. [file 43018_2026_1185_MOESM1_ESM.pdf]

Supplementary information

Individual patient summaries

Cohort 1 (20 mg intrathecal nivolumab): Individual patient summaries

Patient NOA-26-001 was discontinued after the first intrathecal administration due to rapid intracranial disease progression. An SAE of intracranial hemorrhage occurred within a preexisting and progressive brain metastasis and was deemed unrelated to the IMP. The patient died two weeks later due to disease progression.
Primary tumor diagnosis was a melanoma, BRAF V600E mutant.
Concomitant systemic therapy during trial treatment was Dabrafenib/Trametinib.

Patient NOA-26-002 completed the treatment and safety evaluation period without DLTs. This patient died during the follow-up phase from disease progression.
Primary tumor diagnosis was melanoma, BRAF V600E mutant.
Concomitant systemic therapy during trial treatment was Dabrafenib/Trametinib.

Patient NOA-26-003 completed the first safety visit without DLTs. Disease progression in the CNS led to discontinuation before the fourth administration. An SAE of grade 2 colitis occurred during the DLT period and was attributed to concomitant intravenous nivolumab and ipilimumab, not to intrathecal administration. The patient died following the first safety visit due to disease progression.
Primary tumor diagnosis was melanoma, BRAF V600E mutant.
This patient did not receive any additional systemic therapy during trial treatment.

Patient NOA-26-004 completed all six scheduled intrathecal administrations without any DLTs or AEs until Safety Visit 1. Disease remained stable at the CNS and systemic levels at the end of treatment. The patient entered the follow-up phase and continues intrathecal nivolumab treatment outside of the trial protocol.
Primary tumor diagnosis was melanoma, BRAF and NRAS wildtype.
This patient did not receive any additional systemic therapy during trial treatment.

Cohort 2 (30 mg intrathecal nivolumab): Individual patient summaries

Patient NOA-26-005 completed all treatment visits without DLT. An SAE occurred due to Ommaya reservoir dysfunction diagnosed prior to the first IMP administration; the reservoir was replaced. Nausea and vomiting episodes were consistent with the patient's medical history and attributed to underlying disease. Due to disease progression the patient was transferred to a hospice before Follow-up V4 and died.
Primary tumor diagnosis was melanoma, BRAF V600E mutant.
Concomitant systemic therapy during trial treatment was Encorafenib/Binimetinib.

Patient NOA-26-006 completed the study period without DLT. An SAE due to a wound granuloma required minor surgical intervention. Additionally, the patient tested SARS-CoV-2-positive without symptoms. Both events were unrelated to the IMP.

Primary tumor diagnosis was melanoma, BRAF, KIT and NRAS wildtype.
This patient did not receive any additional systemic therapy during trial treatment.

Patient NOA-26-007 developed a skin infection at the Ommaya site leading to non-related SAEs (no DLT) intracerebral abscess and meningitis after the first IMP dose. IMP administration was discontinued after 2nd IMP dose. A subsequent episode of colitis (SAE) was considered unrelated, given the patient's prior history of checkpoint-inhibitor-induced colitis and ileostomy. Once the patient's health status had stabilized, treatment with IT nivolumab was continued outside of the study. The patient completed follow-up 4 and continued with long-term follow-up.
Primary tumor diagnosis was melanoma, BRAF V600E mutant.
Concomitant systemic therapy during trial treatment was Dabrafenib/Trametinib.

Patient NOA-26-008 discontinued treatment after IMP-V1 due to tumor progression; no SAEs occurred.
Primary tumor diagnosis was triple negative breast cancer.
This patient did not receive any additional systemic therapy during trial treatment.

Patient NOA-26-009 was a screening failure.

Patient NOA-26-010 completed three IMP administrations and Safety Visit 1 without DLT or SAE and continued with treatment and follow-up.
Primary tumor diagnosis was triple negative breast cancer.
Concomitant systemic therapy during trial treatment was Capecitabine.

Cohort 3 (40 mg intrathecal nivolumab): Individual patient summaries

In cohort 3, one dose-limiting toxicity (DLT) was observed, triggering cohort expansion to six patients per the 3+3 dose-escalation design. In total, eleven serious adverse events (SAEs) were reported in this cohort, including two CTCAE grade 4 events—"sepsis" and "hepatic failure"—in patient **NOA-26-013**. Both were assessed as related to the investigational medicinal product (IMP) and qualified as suspected unexpected serious adverse reactions (SUSARs). The ethics committee, competent authority, and Data Safety Monitoring Board (DSMB) were informed accordingly in ad-hoc reports and meetings.

Patient NOA-26-013 received three intrathecal administrations of nivolumab and completed Safety Visit 1. Primary tumor diagnosis was melanoma, BRAF V600E mutant, NRAS wildtype.
Concomitant systemic therapy during trial treatment was Dabrafenib/Trametinib.
The patient developed a urinary tract infection leading to sepsis, followed by hepatic failure.
This DLT and associated SAEs were reported in an ad hoc DSMB meeting and discussed in detail with the DSMB. The DSMB assessed both SAEs as likely related to IT nivolumab, potentially exacerbated by concurrent BRAF/MEK inhibition.

We provide here a summary of the ad hoc reportings and DSMB assessments:
Patient NOA-26-013 was the first participant enrolled in Cohort 3 of the IT-PD1/NOA-26 trial and received intrathecal nivolumab at a dose of 40 mg. The 51-year-old patient had a long (> 10 years) medical history of metastatic BRAF-mutated melanoma, with multiple systemic

progressions and treatments including BRAF/MEK inhibition and prior intravenous ipilimumab/nivolumab, the latter discontinued due to immune-related colitis and hepatitis. About 9 years after initial diagnosis, the LMD was diagnosed leading to trial enrollment.
Following two intrathecal administrations, the patient experienced two hospitalizations for bacterial infections, both attributed to urinary tract infection and assessed as unrelated to the study drug. The third intrathecal nivolumab application took place. Four days later, the patient was admitted to a local hospital with rapid clinical deterioration and markedly abnormal laboratory findings, including elevated procalcitonin, CRP, GLDH, ASAT, and ALAT, fulfilling CTCAE grade 4 criteria. The event was promptly reported on June 30 as a serious adverse event (SAE), dose-limiting toxicity (DLT), and SUSAR. Treatment included intravenous antibiotics, and, following a liver biopsy on July 4 showing acute portal and lobular hepatitis compatible with immune-mediated liver injury, high-dose intravenous corticosteroids (prednisolone 100 mg/day).
The patient fully recovered and all laboratory abnormalities resolved completely.

The investigators classified the event as a DLT per protocol and considered the temporal relationship consistent with a nivolumab-related immune toxicity rather than with the ongoing BRAF/MEK inhibition. Intrathecal nivolumab was discontinued for this patient, who returned to standard follow-up and later resumed BRAF/MEK inhibition.

In its initial response, the DSMB concurred that the toxicity was a treatment-related DLT likely attributable to nivolumab, though not necessarily dose-dependent, and potentially exacerbated by concurrent BRAF/MEK therapy. The DSMB found no need for modifications to the study design or exclusion criteria but recommended that future amendments explicitly state that concomitant anticancer therapy may be continued and that increased toxicity risks be reflected in the patient information and consent form. The Board also requested clarification regarding corticosteroid dosing, indication, start date, and revisions to the causality assessment in the SAE report.

After reviewing additional source documents and a detailed written response from investigators, the DSMB confirmed that the presentation met the formal definition of a DLT. However, the Board emphasized that alternative causes, particularly broad-spectrum antibiotics and multiple concomitant medications, could equally have contributed to the acute hepatitis. The patient recovered fully from the toxic event and maintained stable disease for approximately six months. Upon later progression, she was re-treated with a checkpoint inhibitor (pembrolizumab) without recurrence of immune-related toxicity.
Patient NOA-26-013 fully recovered from this DLT and SUSAR.

Overall, the DSMB found no indication for safety-driven changes to the ongoing trial but encouraged refinement of patient information materials and careful risk–benefit considerations for heavily pretreated patients.

For Cohort 3, the occurrence of one DLT implies an expansion to six patients under the 3+3 design; however, no immediate changes to the trial protocol were deemed necessary. Based on the DSMB recommendation, we prepared an amendment of patient information and patient consent, and we included the risk of increased toxicity of concurrent BRAF/MEK included in these amended documents.

Patient NOA-26-011: Screening failure due to liver enzyme abnormalities and disease progression.

Patient NOA-26-012: Received one dose before experiencing intracerebral edema; CT imaging confirmed disease progression. Treatment was discontinued.
Primary tumor diagnosis was SCLC.
Concomitant systemic therapy during trial treatment was Sorotasib.

Patient NOA-26-014: Initial leptomeningeal contrast enhancement was not confirmed on repeat imaging; classified as screening failure.

Patient NOA-26-015: Screening failure due to systemic disease progression and lack of treatment eligibility.

Patient NOA-26-016: Completed treatment and both safety visits. Experienced one non-related SAE, bacterial meningitis (CTCAE grade 4), successfully treated with intravenous antibiotics and corticosteroids. A non-IMP-related myocardial infarction (CTCAE grade 3) led to temporary interruption of BRAF/MEK inhibitors. The patient continued intrathecal nivolumab during follow-up.
Primary tumor diagnosis was melanoma, BRAF V600K mutant, NRAS and cKIT wildtype.
Concomitant systemic therapy during trial treatment was Dabrafenib/Trametinib.

Patient NOA-26-017 completed the treatment and safety phase without DLTs and continues intrathecal nivolumab in long-term follow-up.
Primary tumor diagnosis was NSCLC, PD-L1 ≥ 50%, with EML4::ALK translocation.
Concomitant systemic therapy during trial treatment was Brigatinib.

Patient NOA-26-018 received two doses; due to tumor progression, the patient discontinued the treatment.
Primary tumor diagnosis was melanoma, BRAF-/NRAS-/KIT- wildtype.
This patient did not receive any additional systemic therapy during trial treatment.

Patient NOA-26-019 completed treatment and safety phase without DLTs and continues intrathecal nivolumab in follow-up.
Primary tumor diagnosis was melanoma, BRAF V600E mutant.
Concomitant systemic therapy during trial treatment was Dabrafenib/Trametinib.

Patient NOA-26-020 completed three doses and the first safety visit without DLTs or SAEs. Treatment was then discontinued after hospitalization and tumor progression. Three SAEs unrelated to IMP were reported. The patient received palliative care and died shortly after.
Primary tumor diagnosis was a SCLC.
Concomitant systemic therapy during trial treatment was Paclitaxel.

Patient NOA-26-021 received one dose. Only after this first injection, the investigators became aware that the patient had 16 mg dexamethasone during the screening period and 12 mg dexamethasone at IMP-V1. Investigators and sponsor decided that this was a protocol deviation and that this patient had been ineligible for the trial due to this high dexamethasone

dose. A total of four SAEs were reported, including a grade 2 vomiting episode and a CTCAE grade 3 sigmoid perforation requiring extensive surgery. A grade 4 delirium (unrelated to IMP) occurred and led to death, likely in combination with disease progression. The case was discussed with the DSMB and was not classified as a dose-limiting toxicity (DLT). Due to the high dose of dexamethasone at study entry (and violation of includion criteria), the patient was classified as a screening failure.

Patient NOA-26-022 received two doses. One SAE (vomiting, grade 3, not related to IMP) due to cerebral edema was reported. Treatment was discontinued after tumor progression. Primary tumor diagnosis was NSCLC, with EML4/ALK translocation.
Concomitant systemic therapy during trial treatment was Lorlatinib.

Patient NOA-26-023 received all three doses and completed the first safety visit without DLTs or SAEs. The patient continued intrathecal nivolumab treatment.
Primary tumor diagnosis was NSCLC, PD-L1<50%, EML4/ALK translocation.
This patient did not receive any additional systemic therapy during trial treatment.

As only one of six patients in cohort 3 experienced a DLT, the MTD was not exceeded, and the trial continued to the next dose level.

Cohort 4 (50 mg intrathecal nivolumab): Individual patient summaries

Patient NOA-26-024: Received IMP V1–V3 and completed the first safety visit without any DLT or SAE. Due to disease progression, treatment was discontinued after IMP V4. No further visits were possible due to clinical deterioration.
Primary tumor diagnosis was triple negative breast cancer.
This patient did not receive any additional systemic therapy during trial treatment.

Patient NOA-26-025: Completed IMP V1–V3 and Safety Visit 1 without DLT or SAE. At Visit 4, IT nivolumab was not administered due to a wound infection at the Ommaya reservoir to prevent meningitis. Treatment was stopped after disease progression.
Primary tumor diagnosis was NSCLC, EML4/ALK translocation absent.
Concomitant systemic therapy during trial treatment was Docetaxel, Nintedanib and Bevacizumab. Docetaxel/Bevacizumab was discontinued after Safety V1 and Nintendanib was discontinued after IMP2.

Patient NOA-26-026: Screening failure due to clinical deterioration prior to treatment initiation.

Patient NOA-26-027: Received IMP V1–V3 and Safety Visit 1 without DLT.
After IMP V1, two SAEs occurred: Fever (up to 38.8°C) resolved after paracetamol and aphasia (no seizure observed; CT and CSF without any evidence for stroke or infection). Empirical antibiotics (vancomycin, meropenem) and levetiracetam were given. Both events were considered related to the study drug and reported as SUSARs but did not qualify as DLTs. The patient fully recovered and continued treatment. Patient completed Safety Visit 1 and proceeded with treatment and follow-up as scheduled.
Primary tumor diagnosis was melanoma, BRAF V600E mutant, NRAS wildtype.

Concomitant systemic therapy during trial treatment was Dabrafenib/Trametinib and was discontinued after Safety V1 visit.

Summary of Initial Patients in Cohort 4: Four patients were screened. One (01-026) was a screening failure. Three patients (08-024, 01-025, 06-027) received IMP V1–V3 and completed the first safety visit without any DLT. The DSMB recommended to enroll additional 3 patients in Cohort 4 to confirm safety at the highest dose level.

Patient NOA-26-028 received all six intrathecal nivolumab doses. No SAEs occurred. MRI showed progression with at least two new brain metastases; stereotactic radiotherapy is planned. Clinically stable and in follow-up.
Primary tumor diagnosis was a NSCLC, PD-L1 >50%.
Concomitant systemic therapy during trial treatment was Topotecan.

Patient NOA-26-029 completed all six doses. One Grade 2 SAE ("CRP elevation") occurred; the patient was hospitalized due to elevated infection markers but discharged after 3 days with no confirmed infection. The event was deemed unrelated to nivolumab. Clinical and imaging progression, including trochlear nerve palsy (causing double vision) and facial nerve impairment. Whole-brain radiotherapy was scheduled.
Primary tumor diagnosis was triple negative breast cancer.
Concomitant systemic therapy during trial treatment was Capecitabine, Tucatinib, Trenantone, Exemestan, Trastuzumab.
Capecitabine was stopped after IMP1.
During the follow-up period, Tucatinib was stopped after FU1.

Patient NOA-26-30 received IMP V1–V3 and Safety Visit 1 without any AE or SAE and proceeded with study treatment.
Primary tumor diagnosis was melanoma, BRAF V600E mutant.
Concomitant systemic therapy during trial treatment was Encrorafenib/Binimetinib.
