# Supplementary material for: Intrathecal nivolumab in metastatic solid tumors with leptomeningeal disease: dose escalation part of the multicenter IT-PD1/NOA-26 phase 1 trial
Source: Nat Cancer. 2026 Jun 4;7(7):1094–103. doi: 10.1038/s43018-026-01185-4 (PMC13400297; doi:10.1038/s43018-026-01185-4)
Supplement: Supplementary file 2 — Supplementary Tables 1–3. [file 43018_2026_1185_MOESM2_ESM.pdf]

Supplementary Table 1:

Number of patients (ITT population, n=24) who completed patient-reported outcomes and neurocognitive screening tests

**Visit No**	**1**	**2**	**3**	**4**	**5**	**6**	**7**	**8**	**9**	**10**	**11**	**12**	**13**
**Completed by**													
**EORTC QLQ C30**	24	23	20	17	17	15	12	12	13	13	9	8	5
**EORTC QLQ BN20**	24	23	20	17	17	15	12	12	13	13	9	8	5
**MMST**	*	23	21	16	*	13	12	12					
**MoCA**	*	23	20	17	*	13	12	12					
**Distress Score**	24	23	20	17	17	14	12	12	13	13	9	8	5

*MoCA and MMST were not documented

Visit Numbers

1: Baseline
2: IMP Visit 1
3: IMP Visit 2
4: IMP Visit 3
5: Safety Visit 1#
6: IMP Visit 4
7: IMP Visit 5
8: IMP Visit 6
9: Safety Visit 2
10: Follow-up 1
11: Follow-up 2
12: Follow-up 3
13: Follow-up 4

Supplementary Table 2: Systemic Therapies prior to enrolment and during trial treatment

ID	Concomitant systemic tumor therapies	Systemic tumor therapies prior to LMD diagnosis and trial enrollment*
**NOA-26-001**	Dabrafenib/Trametinib	Dabrafenib/Trametinib, Ipilimumab/Nivolumab, Nivolumab, Pembrolizumab
**NOA-26-002**	Dabrafenib/Trametinib	Dabrafenib/Trametinib, Ipilimumab/Nivolumab, Nivolumab
**NOA-26-003**	None	Nivolumab, Dabrafenib/Trametinib, Ipilimumab/Nivolumab
**NOA-26-004**	None	Nivolumab, Ipilimumab/Nivolumab, Nivolumab
**NOA-26-005**	Encorafenib/Binimetinib	Nivolumab, Nivolumab/Ipilimumab, Encorafenib/Binimetinib
**NOA-26-006**	None	Nivolumab/Ipilimumab, Nivolumab
**NOA-26-007**	Dabrafenib/Trametinib	Nivolumab/Ipilimumab, Dabrafenib/Trametinib, Infliximab, Ustekinumab, Nivolumab/Ipilimumab
**NOA-26-008**	None	Carboplatin/nab-Paclitaxel/Pembrolizumab, Epirubicin/Cyclophosphamid
**NOA-26-010**	Capecitabine	Epirubicin/Cyclophosphamid, Paclitaxel/Carboplatin
**NOA-26-012**	Sorotasib	Cisplatin/Vinorelbin, Carboplatin/Pemetrexed/Pembrolizumab, Docetaxel/Ramucirumab, Methotrexat
**NOA-26-013**	Dabrafenib/Trametinib	Dabrafenib/Trametinib, Ipilimumab/Nivolumab
**NOA-26-016**	Dabrafenib/Trametinib	Pembrolizumab, Dabrafenib/Trametinib, Ipilimumab/Nivolumab,
**NOA-26-017**	Brigatinib	Alectinib/Biphosphonate
**NOA-26-018**	None	Ipilimumab/Nivolumab
**NOA-26-019**	Dabrafenib/Trametinib	Ipilimumab/Nivolumab, Nivolumab, Dabrafenib/Trametinib, Pembrolizumab
**NOA-26-020**	Paclitaxel	Carboplatin AUC5/Etoposid, Topotecan
**NOA-26-021**	None	Cisplatin/Vinorelbine
**NOA-26-022**	Lorlatinib	Brigatinib, Crizotinb, Bevacizumab
**NOA-26-023**	None	Paclitaxel/Cisplatin, Vinorelbine
**NOA-26-024**	None	Paclitaxel/Epirubicin
**NOA-26-025**	Docetaxel/Nintedanib/Bevacizumab	Carboplatin/Nab-Paclitaxel, Atezolizumab, Denusomab, Carboplatin/Pemetrexed, Bevacizumab
**NOA-26-027**	Dabrafenib/Trametinib	Dabrafenib/Trametinib, Nivolumab/Ipilimumab, Nivolumab, Encorafenib/Binimetinib
**NOA-26-028**	Topotecan	Cisplatin/Etoposid, Durvalumab
**NOA-26-029**	Capecitabine,Tucatinib, Trenantone, Exemestan, Trastuzumab	Taxotere/Carboplatin/Herceptin/Pertuzumab, Letrozol, Trastuzumab/Deruxtecan
**NOA-26-030**	Encorafenib/Binimetinib	None

**No sequential or new systemic therapies were administered after the IMP6 or Safety Visit 2. Further details are outlined in Supplementary information "Individual Patient Summaries".*

Supplementary Table 3: All Treatment-related Adverse Events until Safety Visit 1

Subject	Description	Term	CTCAE Grading	SAE	Treatment	Description of treatment	Related to IMP	Action	DLT
Cohort 1	n/a	n/a	n/a	n/a	n/a	n/a	n/a	n/a	n/a
Cohort 2									
NOA26-005	Headache intermittent	Nervous system disorders	2 – Moderate	No	Yes	Pain killers	Yes	Dose not changed	No DLT acc. to protocol
NOA26-005	Nausea	Gastrointestinal disorders	2 – Moderate	No	Yes	Ondansetron and granisetron was intake	Yes	Dose not changed	No DLT acc. to protocol
NOA26-005	Vomitting	Gastrointestinal disorders	2- Moderate	No	Yes	Ondansetron was intake	Yes	Dose not changed	No DLT acc. to protocol
NOA26-005	Meningismus intermittent	Nervous system disorders	2- Moderate	No	Yes	Pain killers	Yes	Dose not changed	No DLT acc. to protocol
Cohort 3									
NOA26-013	Sepsis	Infections and infestations	4- Life-threatening	Yes	Yes	i.v. antibiotics with tazobactam	Yes	Drug withdrawn	Yes
NOA26-013	Hepatic failure	Hepatobiliary disorders	4- Life-threatening	Yes	Yes	i.v. antibiotics, Prednisolon, surveillance and hospitalisation	Yes	Drug withdrawn	Yes
NOA26-019	Nausea	Gastrointestinal disorders	2- Moderate	No	Yes	Drug treatment with Ondansetron 4 mg	Yes	Dose not changed	No DLT acc. to protocol
Cohort 4									
NOA26-024	Vomiting	Gastrointestinal disorders	1- Mild	No	Yes	Vomex 50 mg 1-1-1	Yes	Dose not changed	No DLT acc. to protocol
NOA26-024	Headache	Nervous system disorders	1- Mild	No	No	-	Yes	Dose not changed	No DLT acc. to protocol

Subject	Description	Term	CTCAE Grading	SAE	Treatment	Description of treatment	Related to IMP	Action	DLT
NOA26-027	Nausea	Gastrointestinal disorders	2- Moderate	No	Yes	Ondansetron 8 mg	Yes	Dose not changed	No DLT acc. to protocol
NOA26-027	Dizziness	Nervous system disorders	3- Severe	No	No	-	Yes	Dose not changed	No DLT acc. to protocol
NOA26-027	Nausea	Gastrointestinal disorders	3- Severe	No	Yes	Ondansetron 8 mg and Vomex 50 mg	Yes	Dose not changed	No DLT acc. to protocol
NOA26-027	Dysphasia	Nervous system disorders	3- Severe	Yes	Yes	Levetiracetam	Yes	Dose not changed	No DLT acc. to protocol
NOA26-027	Fever	General disorders and administration site conditions	1- Mild	No	Yes	Paracetamol	Yes	Dose not changed	No DLT acc. to protocol
NOA26-027	Vomitting	Gastrointestinal disorders	2- Moderate	No	Yes	Vomex 50 mg once	Yes	Dose not changed	No DLT acc. to protocol
NOA26-027	Fever	General disorders and administration site conditions	1- Mild	No	No	-	Yes	Dose not changed	No DLT acc. to protocol
NOA26-027	Fever	Infections and infestations	1- Mild	No	No	-	Yes	Dose not changed	No DLT acc. to protocol
NOA26-028	Nausea during application i.th. Nivolumab	General disorders and administration site conditions	2- Moderate	No	Yes	Ondansetron 4 mg as required s.l.	Yes	Dose not changed	No DLT acc. to protocol
NOA26-028	Dizziness during application i.th. Nivolumab	General disorders and administration site conditions	1- Mild	No	Yes	Just water drinking, non-drug treatment	Yes	Dose not changed	No DLT acc. to protocol

Subject	Description	Term	CTCAE Grading	SAE	Treatment	Description of treatment	Related to IMP	Action	DLT
NOA26-028	Involuntary movements both legs, also involuntary movement both legs during Treatment nivolumab	General disorders and administration site conditions	1- Mild	No	No	-	Yes	Dose not changed	No DLT acc. to protocol
NOA26-028	Involuntary movement eyelid Right / twitch	General disorders and administration site conditions	1- Mild	No	No	-	Yes	Dose not changed	No DLT acc. to protocol
NOA26-028	Dizziness during application i.th. nivolumab	General disorders and administration site conditions	1- Mild	No	Yes	Drinking water and half lying position	Yes	Dose not changed	No DLT acc. to protocol
NOA26-028	Muscle twitching for 2 days, not daily, for few seconds	Musculoskeletal and connective tissue disorders	1- Mild	No	No	-	Yes	Dose not changed	No DLT acc. to protocol
NOA26-028	Nausea (during application of Nivolumab)	General disorders and administration site conditions	1- Mild	No	Yes	Drinking water & waiting<br><br>Ondansetron 4mg was given on 17.01.2025	Yes	Dose not changed	No DLT acc. to protocol
NOA26-028	Tingling in face and feets	Nervous system disorders	1- Mild	No	No	-	Yes	Dose not changed	No DLT acc. to protocol
NOA26-028	Numbness in the lips and right side of the face	Nervous system disorders	1- Mild	No	No	-	Yes	Dose not changed	No DLT acc. to protocol

Subject	Description	Term	CTCAE Grading	SAE	Treatment	Description of treatment	Related to IMP	Action	DLT
NOA26-028	Headache	General disorders and administration site conditions	1- Mild	No	No	-	Yes	Dose not changed	No DLT acc. to protocol
NOA26-028	Vomiting after application<br><br>nivolumab	Gastrointestinal disorders	2- Moderate	No	Yes	Ondansetron 4 mg	Yes	Dose not changed	No DLT acc. to protocol
NOA26-028	Tingling in face (Right lower lip) and both feets	Nervous system disorders	1- Mild	No	No	-	Yes	Dose not changed	No DLT acc. to protocol
NOA26-028	Tingling both feets and sool of feets	Nervous system disorders	1- Mild	No	No	-	Yes	Dose not changed	No DLT acc. to protocol
NOA26-028	Dizziness during application nivolumab	General disorders and administration site conditions	1 – Mild	No	No	-	Yes	Dose not changed	No DLT acc. to protocol
NOA26-028	Nausea during application nivolumab	General disorders and administration site conditions	1 – Mild	No	No	-	Yes	Dose not changed	No DLT acc. to protocol
NOA26-028	Tingling in both legs	Nervous system disorders	1 – Mild	No	No	-	Yes	Dose not changed	No DLT acc. to protocol
NOA26-028	Nausea	General disorders and administration site conditions	1 – Mild	No	No	-	Yes	Dose not changed	No DLT acc. to protocol
NOA26-028	Nausea during application i.th. Nivolumab	Gastrointestinal disorders	2- Moderate	No	No	-	Yes	Dose not changed	No DLT acc. to protocol

Subject	Description	Term	CTCAE Grading	SAE	Treatment	Description of treatment	Related to IMP	Action	DLT
NOA26-029	Diarrhea	Gastrointestinal disorders	2- Moderate	No	Yes	Loperamid 2 mg as required	Yes	Dose not changed	No DLT acc. to protocol
NOA26-029	Headache	General disorders and administration site conditions	1 – Mild	No	No	-	Yes	Dose not changed	No DLT acc. to protocol
NOA26-029	Tingling in the heels both sides	Nervous system disorders	1 – Mild	No	No	-	Yes	Dose not changed	No DLT acc. to protocol
NOA26-029	Weight loss	Metabolism and nutrition disorders	1 – Mild	No	No	-	Yes	Dose not changed	No DLT acc. to protocol
NOA26-029	Decreased AP	Blood and lymphatic system disorders	2- Moderate	No	No	-	Yes	Dose not changed	No DLT acc. to protocol
NOA26-029	Decreased bilirubin	Blood and lymphatic system disorders	2- Moderate	No	No	-	Yes	Dose not changed	No DLT acc. to protocol
NOA26-029	Tingling in both feets	Nervous system disorders	1 – Mild	No	No	-	Yes	Dose not changed	No DLT acc. to protocol
NOA26-029	Gait uncertainty	Nervous system disorders	1 – Mild	No	No		Yes	Dose not changed	No DLT acc. to protocol
NOA26-029	Double Vision for short time intermittently	Eye disorders	1 – Mild	No		-	Yes	Dose not changed	No DLT acc. to protocol
NOA26-029	Headache	Nervous system disorders	1 – Mild	No	No	-	Yes	Dose not changed	No DLT acc. to protocol

Subject	Description	Term	CTCAE Grading	SAE	Treatment	Description of treatment	Related to IMP	Action	DLT
NOA26-029	Chills	General disorders and administration site conditions	1 – Mild	No	No	-	Yes	Dose not changed	No DLT acc. to protocol
NOA26-029	Paresis N. Trochlearis	Eye disorders	2- Moderate	No	Yes	Masking of the right or left eye possible for binocular double Vision<br>+ cMR recommended	Yes	Dose not changed	No DLT acc. to protocol
NOA26-029	Dizziness	General disorders and administration site conditions	1 – Mild	No	No	-	Yes	Dose not changed	No DLT acc. to protocol
NOA26-029	Fatigue	General disorders and administration site conditions	2- Moderate	No	Yes	Resting, Taking naps, regulated activities	Yes	Dose not changed	No DLT acc. to protocol

Supplementary Table 4: All Adverse Events until Safety Visit 1

Subject	Description	Term	CTCAE Grading	SAE	Treatment	Description of treatment	Related to IMP	Action	DLT
NOA26-001	Lymphopenia	Blood and lymphatic system disorders	3- Severe	No	No	-	No	Dose not changed	No DLT acc. to protocol
NOA26-001	Intracranial hemorrhage	Nervous system disorders	4- Life-threatening	Yes	Yes	Surgical procedure	No	Drug withdrawn	No DLT acc. to protocol
NOA26-002	Lymphopenia	Blood and lymphatic system disorders	3- Severe	No	No	-	No	Dose not changed	No DLT acc. to protocol
NOA26-002	Low vitamine D3	Blood and lymphatic system disorders	1 – Mild	No	Yes	Vitamin D3 is taken	No	Dose not changed	No DLT acc. to protocol
NOA26-003	Diarrhea	Gastrointestinal disorders	2- Moderate	No	No	-	No	Dose not changed	No DLT acc. to protocol
NOA26-003	Colitis	Gastrointestinal disorders	2- Moderate	Yes	Yes	Prednisolon 50mg / day	No	Dose not changed	No DLT acc. to protocol
NOA26-003	Dysphasia	Nervous system disorders	3- Severe	No	No	-	No	Dose not changed	No DLT acc. to protocol
NOA26-003	Constipation	Gastrointestinal disorders	1 – Mild	No	Yes	Laxoberal was given	No	Dose not changed	No DLT acc. to protocol

Subject	Description	Term	CTCAE Grading	SAE	Treatment	Description of treatment	Related to IMP	Action	DLT
NOA26-004	Metastasis right tibia	Skin and subcutaneous tissue disorders	2- Moderate	No	Yes	Radiation	No	Dose not changed	No DLT acc. to protocol
NOA26-005	Anemia	Blood and lymphatic system disorders	2- Moderate	No	No	-	No	Dose not changed	No DLT acc. to protocol
NOA26-005	Revision of Ommaya reservoir (Dysfunction)	Surgical and medical procedures	3- Severe	No	Yes	Revision of shunt	No	Dose not changed	No DLT acc. to protocol
NOA26-005	Headache intermittent	Nervous system disorders	2- Moderate	No	Yes	Pain killers	Yes	Dose not changed	No DLT acc. to protocol
NOA26-005	Nausea	Gastrointestinal disorders	2- Moderate	No	Yes	andosetron and granisetron was intake	Yes	Dose not changed	No DLT acc. to protocol
NOA26-005	Vomitting	Gastrointestinal disorders	2- Moderate	No	Yes	Ondansetron was intake	Yes	Dose not changed	No DLT acc. to protocol
NOA26-005	Meningism intermittant	Nervous system disorders	2- Moderate	No	Yes	Pain killers	Yes	Dose not changed	No DLT acc. to protocol
NOA26-006	Headache	Nervous system disorders	3- Severe	No	Yes	Ibuprofen and Novaminsulfon was given	n.a. (Date of onset prior to first administra tion of IMP)	n.a.	No DLT acc. to protocol

Subject	Description	Term	CTCAE Grading	SAE	Treatment	Description of treatment	Related to IMP	Action	DLT
NOA26-006	Constipation	Gastrointestinal disorders	2- Moderate	No	Yes	Laxoberal was given	n.a. (Date of onset prior to first administra tion of IMP)	Dose not changed	No DLT acc. to protocol
NOA26-006	Wound complication	Injury, poisoning and procedural complications	2- Moderate	Yes	Yes	Surgery<br><br>antibiotics	No	Dose not changed	No DLT acc. to protocol
NOA26-006	Sars-Cov-2-Infection	Infections and infestations	1 – Mild	Yes	No	-	n.a. (Date of onset prior to first administra tion of IMP)	Dose not changed	No DLT acc. to protocol
NOA26-006	Headache	Nervous system disorders	1 – Mild	No	Yes	Ibuprofen	No	Dose not changed	No DLT acc. to protocol
NOA26-006	Fatigue	General disorders and administration site conditions	1 – Mild	No	No	-	No	Dose not changed	No DLT acc. to protocol
NOA26-006	Memory impairment	Nervous system disorders	1 – Mild	No	No	-	No	Dose not changed	No DLT acc. to protocol
NOA26-007	Wound infection	Infections and infestations	1 – Mild	No	Yes	Antibiotics, wound revision, explantation of Ommaya reservoir	No	Dose not changed	No DLT acc. to protocol

Subject	Description	Term	CTCAE Grading	SAE	Treatment	Description of treatment	Related to IMP	Action	DLT
NOA26-007	Meningitis	Infections and infestations	3- Severe	Yes	Yes	Antibiotics	No	Drug withdrawn	No DLT acc. to protocol
NOA26-007	Intracerebral abscess	Infections and infestations	3- Severe	Yes	Yes	iv and it antibiotics, revision of abscess, surgery	No	Drug withdrawn	No DLT acc. to protocol
NOA26-007	Colitis	Gastrointestinal disorders	3- Severe	Yes	No	-	No	n.a.	No DLT acc. to protocol
NOA26-008	Lymphocyte count decreased	Investigations	3- Severe	No	Yes	Drug treatment: cotrim forte	No	Dose not changed	No DLT acc. to protocol
NOA26-010	Urinary tract infection	Renal and urinary disorders	2- Moderate	No	Yes	Venofloxacin	No	Dose not changed	No DLT acc. to protocol
NOA26-010	Seizure	Nervous system disorders	2- Moderate	No	Yes	Levetiracetam increased	No	Dose not changed	No DLT acc. to protocol
NOA26-010	Hypokaliemia	Blood and lymphatic system disorders	2- Moderate	No	Yes	Potassium intake	No	Dose not changed	No DLT acc. to protocol
NOA26-010	Hemorrhoid bleeding	Infections and infestations	1 – Mild	No	No	-	No	Dose not changed	No DLT acc. to protocol

Subject	Description	Term	CTCAE Grading	SAE	Treatment	Description of treatment	Related to IMP	Action	DLT
NOA26-012	Telangiectasia back and neck	Skin and subcutaneous tissue disorders	1 – Mild	No	No	-	n.a. (Date of onset prior to first administra tion of IMP)	n.a.	No DLT acc. to protocol
NOA26-012	Respiratory disorder, other: extenuated respiratory sound	Respiratory, thoracic and mediastinal disorders	1 – Mild	No	No	--	n.a. (Date of onset prior to first administra tion of IMP)	n.a.	No DLT acc. to protocol
NOA26-012	Edema limbs	General disorders and administration site conditions	1 – Mild	No	No	-	n.a. (Date of onset prior to first administra tion of IMP)	n.a.	No DLT acc. to protocol
NOA26-012	Eye disorders, other: flickering of the eyes, right	Eye disorders	1 – Mild	No	No	-	n.a. (Date of onset prior to first administra tion of IMP)	n.a.	No DLT acc. to protocol
NOA26-012	Hearing impaired: hypakusis	Ear and labyrinth disorders	1 – Mild	No	No	-	n.a. (Date of onset prior to first administra tion of IMP)	n.a.	No DLT acc. to protocol

Subject	Description	Term	CTCAE Grading	SAE	Treatment	Description of treatment	Related to IMP	Action	DLT
NOA26-012	Vomiting	Gastrointestinal disorders	2- Moderate	No	Yes	8 mg Ondansetron i.v.	No	Dose not changed	No DLT acc. to protocol
NOA26-012	Nausea	Gastrointestinal disorders	3- Severe	No	Yes	8 mg Ondansetron i.v.	No	Dose not changed	No DLT acc. to protocol
NOA26-012	Somnolence	Nervous system disorders	2- Moderate	No	No	-	No	Dose not changed	No DLT acc. to protocol
NOA26-012	Intracerebral edema	Nervous system disorders	3- Severe	Yes	Yes	Increasing Dexamethason from 1,5 mg/d to 4 mg/d (11-MAY-2023) and to 2 x 8 mg/d (since 12-MAY-2023)--> end of study	No	Dose not changed	No DLT acc. to protocol
NOA26-012	Pain in muscles	General disorders and administration site conditions	2- Moderate	No	Yes	Novaminsulfon i.v. 4 x 1 g/d			No DLT acc. to protocol
NOA26-012	Pruritus	Skin and subcutaneous tissue disorders	2- Moderate	No	Yes	Fenistil Creme, Tropfen und i.v.(4 mg/d starting 13-MAY-2023)	No		No DLT acc. to protocol
NOA26-013	Bacterial infection	Infections and infestations	3- Severe	Yes	Yes	i.v. antibotics with Meropenem	No	Dose not changed	No DLT acc. to protocol
NOA26-013	Sepsis	Infections and infestations	4- Life-threatening	Yes	Yes	i.v. antibiotics with Tazobactam	Yes	Drug withdrawn	Yes

Subject	Description	Term	CTCAE Grading	SAE	Treatment	Description of treatment	Related to IMP	Action	DLT
NOA26-013	Hepatic failure	Hepatobiliary disorders	4- Life-threatening	Yes	Yes	i.v. antibiotics, Prednisolon, surveillance and hospitalisation	Yes	Drug withdrawn	Yes
NOA26-016	Bacterial meningitis	Infections and infestations	1 – Mild	Yes	Yes	i.v. antibiotics with Meropenem and Vancomycin and prednisolon. No surgical teatment	No	n.a.	No DLT acc. to protocol
NOA26-016	Acute renal failure due to therapy with vancomycin	Renal and urinary disorders	3- Severe	No	Yes	i.v. fluids, human albumin tranfusions, daily blood taking and controll of electrolytes	No	n.a.	No DLT acc. to protocol
NOA26-016	Fatigue	General disorders and administration site conditions	2- Moderate	No	No	-	No	Dose not changed	No DLT acc. to protocol
NOA26-016	Myocardial infarction	Cardic disorders	3- Severe	Yes	Yes	clicinical monitoring, BRAF MEK inhibition was paused	No	Dose not changed	No DLT acc. to protocol
NOA26-017	Urinary tract infection	Infections and infestations	2- Moderate	No	Yes	Fluconazol 100 mg 10 days	No	n.a.	No DLT acc. to protocol
NOA26-017	Leucopenia intermittant	Blood and lymphatic system disorders	3- Severe	No	Yes	Filgastrim 30MIO IE was given	No	Dose not changed	No DLT acc. to protocol

Subject	Description	Term	CTCAE Grading	SAE	Treatment	Description of treatment	Related to IMP	Action	DLT
NOA26-017	Hyponatriemia	Blood and lymphatic system disorders	2- Moderate	No	Yes	sodium chloride tablet, NaCl 0,9% 500 ml iv	No	Dose not changed	No DLT acc. to protocol
NOA26-017	Nycturia	Renal and urinary disorders	2- Moderate	No	No	-	No	Dose not changed	No DLT acc. to protocol
NOA26-017	Cystitis	Renal and urinary disorders	3- Severe	No	Yes	Fluconazol	No	Dose not changed	No DLT acc. to protocol
NOA26-017	Urinary tract infection	Renal and urinary disorders	2- Moderate	No	Yes	Fluconazol<br><br>Ampicillin	No	Dose not changed	No DLT acc. to protocol
NOA26-017	Urinary tract infection	Renal and urinary disorders	2- Moderate	No	Yes	Nitrofurantion 100 mg	No	Dose not changed	No DLT acc. to protocol
NOA26-018	Urinary retention	Renal and urinary disorders	2- Moderate	No	Yes	Pubic catheter	No	Drug withdrawn	No DLT acc. to protocol
NOA26-018	Memory impairment	Nervous system disorders	2- Moderate	No	No	-	No	Drug withdrawn	No DLT acc. to protocol
NOA26-018	Pyraidal tract symptoms:tetra paresis	Nervous system disorders	3- Severe	No	No	-	No	Drug withdrawn	No DLT acc. to protocol

Subject	Description	Term	CTCAE Grading	SAE	Treatment	Description of treatment	Related to IMP	Action	DLT
NOA26-018	Neuralgia, severe neuropathic pain	Nervous system disorders	3- Severe	No	Yes	i.v. treatment with hydromorphon was escalated, Dexamethason and metamizol was given add on	No	Drug withdrawn	No DLT acc. to protocol
NOA26-018	Somnolence	Nervous system disorders	3- Severe	No	No	-	No	Drug withdrawn	No DLT acc. to protocol
NOA26-018	Severe nausea	Gastrointestinal disorders	3- Severe	No	Yes	Levomepromazin, Dimenhydrinat, Butylscopalamin	No	Drug withdrawn	No DLT acc. to protocol
NOA26-018	Constipation	Gastrointestinal disorders	2- Moderate	No	Yes	Methylnaltrexon i.v.; Pyrilax supp.	No	Drug withdrawn	No DLT acc. to protocol
NOA26-018	Pyramidal tract symptoms, paraparesis	Nervous system disorders	2- Moderate	No	No	-	No	Dose not changed	No DLT acc. to protocol
NOA26-019	Nausea	Gastrointestinal disorders	2- Moderate	No	Yes	Drug treatment with ondansetron 4 mg	Yes	Dose not changed	No DLT acc. to protocol
NOA26-019	Anemia due to iron deficiency	Blood and lymphatic system disorders	3- Severe	No	Yes	Substitution of iron	No	Dose not changed	No DLT acc. to protocol
NOA26-019	Skin metastasis left forehead	Skin and subcutaneous tissue disorders	3- Severe	No	Yes	Resection of metastasis on 29-DEC-2023	No	Dose not changed	No DLT acc. to protocol

Subject	Description	Term	CTCAE Grading	SAE	Treatment	Description of treatment	Related to IMP	Action	DLT
NOA26-019	Wound healing disorder	Skin and subcutaneous tissue disorders	2- Moderate	No	No	-	No	Dose not changed	No DLT acc. to protocol
NOA26-019	Disease progression, cutanous metastase	Skin and subcutaneous tissue disorders	2- Moderate	No	No	-	No	Dose not changed	No DLT acc. to protocol
NOA26-020	Seizure	Nervous system disorders	3- Severe	Yes	Yes	Levetiracetam 2x500 mg/day	No	Dose not changed	No DLT acc. to protocol
NOA26-020	General condition after intracerebral hemorraghe into a CNS-metastatis has not ameliorated. Patient was still desoriented and suffered from dysphagia. Palliative care setting	General disorders and administration site conditions	5- Death	Yes	No	-	No	n.a.	No DLT acc. to protocol
NOA26-020	Intracranial hemmorhage	Nervous system disorders	3- Severe	Yes	No	-	No	Dose not changed	No DLT acc. to protocol

Subject	Description	Term	CTCAE Grading	SAE	Treatment	Description of treatment	Related to IMP	Action	DLT
NOA26-021	Vomitting	Gastrointestinal disorders	2- Moderate	Yes	Yes	Unacid 3g 3xday	n.a. (Date of onset prior to first administra tion of IMP)	n.a.	No DLT acc. to protocol
NOA26-021	Malaise	General disorders and administration site conditions	3- Severe	Yes	No	-	No	Dose not changed	No DLT acc. to protocol
NOA26-021	Sigmoid perforation retroperitoneall y and into the small bowel meso with associated abscess	Gastrointestinal disorders	3- Severe	Yes	Yes	Patient had a surgery on 18 December 2023: Sigmoid resection as Hartmann situation, partial omentectomy, resection of the small intestine, abscess removal, irrigation drainage; Iv antibiosis with meropenem; Stoma application on 18-DEC-2023	No	Dose not changed	No DLT acc. to protocol
NOA26-021	Sigmoid diverticulitis	Gastrointestinal disorders	1- Mild	No	Yes	Antibiotics Unacid, Tazobac	No	Dose not changed	No DLT acc. to protocol

Subject	Description	Term	CTCAE Grading	SAE	Treatment	Description of treatment	Related to IMP	Action	DLT
NOA26-021	Electrolyte deficiency	General disorders and administration site conditions	2- Moderate	No	Yes	Jonosteril	No	Dose not changed	No DLT acc. to protocol
NOA26-021	Delirium	General disorders and administration site conditions	4- Life-threatening	Yes	Yes	Infusion with Jono steril	No	Drug withdrawn	No DLT acc. to protocol
NOA26-022	Vomitting	Gastrointestinal disorders	3- Severe	Yes	Yes	MRI brain	No	Dose not changed	No DLT acc. to protocol
NOA26-022	Vision decreased	Eye disorders	1- Mild	No	No	-	No	Dose not changed	No DLT acc. to protocol
NOA26-023	Oral mycosis	Infections and infestations	2- Moderate	No	Yes	Amphotericin B local: 100 mg/ml 1 ml 1-1-1-1	No	Dose not changed	No DLT acc. to protocol
NOA26-024	Dysfunction Ommaya Reservoir	Surgical and medical procedures	3- Severe	No	Yes	surgery of new Ommaya Reservoir 29-AUG-2024	No	Dose not changed	No DLT acc. to protocol
NOA26-024	Neurocognition deficit	Nervous system disorders	1- Mild	No	No	-	n.a. (Date of onset prior to first administration of IMP)	n.a.	No DLT acc. to protocol
NOA26-024	Vomiting	Gastrointestinal disorders	1- Mild	No	Yes	Vomex 50 mg 1-1-1	Yes	Dose not changed	No DLT acc. to protocol

Subject	Description	Term	CTCAE Grading	SAE	Treatment	Description of treatment	Related to IMP	Action	DLT
NOA26-024	Neurocognition deficit	Nervous system disorders	1- Mild	No	No	-	No	Dose not changed	No DLT acc. to protocol
NOA26-024	Headache	Nervous system disorders	1- Mild	No	No	-	Yes	Dose not changed	No DLT acc. to protocol
NOA26-024	Leukocytes high, maybe related to Pegfilgastrim 03.10.2024, Filgastrim 29.09.2024	Blood and lymphatic system disorders	1- Mild	No	No	-	No	Dose not changed	No DLT acc. to protocol
NOA26-024	Lymphocytes low, maybe because of Pegfilgastrim 03.10.2024, Filgastrim 29.09.2024	Blood and lymphatic system disorders	1- Mild	No	No	-	No	Dose not changed	No DLT acc. to protocol
NOA26-025	Liver toxicity (ALT)	Blood and lymphatic system disorders	1- Mild	No	No	--	n.a. (Date of onset prior to first administra tion of IMP)	n.a.	No DLT acc. to protocol

Subject	Description	Term	CTCAE Grading	SAE	Treatment	Description of treatment	Related to IMP	Action	DLT
NOA26-025	Liver toxicity (AST)	Blood and lymphatic system disorders	1- Mild	No	No	-	n.a. (Date of onset prior to first administra tion of IMP)	n.a.	No DLT acc. to protocol
NOA26-025	Ihrombosis	Blood and lymphatic system disorders	2- Moderate	No	Yes	Drug treatment Clexane 60 mg 1-0-1	No	n.a.	No DLT acc. to protocol
NOA26-025	Increased Gamma GT	Blood and lymphatic system disorders	3- Severe	No	No	-	n.a. (Date of onset prior to first administra tion of IMP)	n.a.	No DLT acc. to protocol
NOA26-025	White blood cell decreased	Blood and lymphatic system disorders	4- Life-threatening	No	Yes	Drug treatment Filgrastim	No	Dose not changed	No DLT acc. to protocol
NOA26-025	Neutropenia	Blood and lymphatic system disorders	4- Life-threatening	No	Yes	Drug treatment Filgrastim	No	Dose not changed	No DLT acc. to protocol
NOA26-025	Stomatitis	Gastrointestinal disorders	3- Severe	No	Yes	Drug treatment Amphotericin B	No	Dose not changed	No DLT acc. to protocol
NOA26-025	Wound infection	Surgical and medical procedures	3- Severe	Yes	Yes	Clindamycin	No	Drug withdrawn	No DLT acc. to protocol
NOA26-025	Meningitis	Infections and infestations	3- Severe	Yes	Yes	Vancomycin, Rifampicin	No	Drug withdrawn	No DLT acc. to protocol

Subject	Description	Term	CTCAE Grading	SAE	Treatment	Description of treatment	Related to IMP	Action	DLT
NOA26-025	Wound complication	Infections and infestations	3- Severe	Yes	Yes	Ssurgery	No	Drug withdrawn	No DLT acc. to protocol
NOA26-025	Stomatitis	Infections and infestations	2- Moderate	No	Yes	Amphomoronal	No	Dose not changed	No DLT acc. to protocol
NOA26-025	Acute kidney injury	Injury, poisoning and procedural complications	3- Severe	No	Yes	Sterofundin was given	No	n.a.	No DLT acc. to protocol
NOA26-025	Hypokaliemia intermittant	Blood and lymphatic system disorders	1- Mild	No	Yes	Potassium substitution	n.a. (Date of onset prior to first administra tion of IMP)	Dose not changed	No DLT acc. to protocol
NOA26-025	Paresthesia anogenital	Nervous system disorders	2- Moderate	No	No	-	No	Dose not changed	No DLT acc. to protocol
NOA26-025	Muscle weakness	Musculoskeletal and connective tissue disorders	2- Moderate	No	No	-	No	Dose not changed	No DLT acc. to protocol
NOA26-027	Nausea	Gastrointestinal disorders	2- Moderate	No	Yes	Ondansetron 8 mg i.V. on 24-OCT-2024	Yes	Dose not changed	No DLT acc. to protocol
NOA26-027	Dizziness	Nervous system disorders	3- Severe	No	No	-	Yes	Dose not changed	No DLT acc. to protocol

Subject	Description	Term	CTCAE Grading	SAE	Treatment	Description of treatment	Related to IMP	Action	DLT
NOA26-027	Hyponatremia	Blood and lymphatic system disorders	1- Mild	No	Yes	Volume administration	No	Dose not changed	No DLT acc. to protocol
NOA26-027	Hypotonia	Cardic disorders	1- Mild	No	Yes	Volume administration	No	Dose not changed	No DLT acc. to protocol
NOA26-027	Febrile neutropenia	Blood and lymphatic system disorders	3- Severe	Yes	Yes	Neupogen	No	n.a.	No DLT acc. to protocol
NOA26-027	Nausea	Gastrointestinal disorders	3- Severe	No	Yes	Ondansetron 8 mg and Vomex 50 mg	Yes	Dose not changed	No DLT acc. to protocol
NOA26-027	Dysphasia	Nervous system disorders	3- Severe	Yes	Yes	Levetiracetam	Yes	Dose not changed	No DLT acc. to protocol
NOA26-027	Fever	General disorders and administration site conditions	1- Mild	No	Yes	Paracetamol	Yes	Dose not changed	No DLT acc. to protocol
NOA26-027	Diarrhea	Gastrointestinal disorders	2- Moderate	No	No	-	No	Dose not changed	No DLT acc. to protocol
NOA26-027	Fatigue	General disorders and administration site conditions	1- Mild	No	No	-	No	Dose not changed	No DLT acc. to protocol

Subject	Description	Term	CTCAE Grading	SAE	Treatment	Description of treatment	Related to IMP	Action	DLT
NOA26-027	Vomitting	Gastrointestinal disorders	2- Moderate	No	Yes	Vomex 50 mg once	Yes	Dose not changed	No DLT acc. to protocol
NOA26-027	Fever	General disorders and administration site conditions	1- Mild	No	No	-	Yes	Dose not changed	No DLT acc. to protocol
NOA26-027	Fever	Infections and infestations	1- Mild	No	No	-	Yes	Dose not changed	No DLT acc. to protocol
NOA26-028	Erythrozyten decreased	Blood and lymphatic system disorders	2- Moderate	No	No	-	n.a. (Date of onset prior to first administration of IMP)	n.a.	No DLT acc. to protocol
NOA26-028	Anemia	Blood and lymphatic system disorders	2- Moderate	No	No	-	n.a. (Date of onset prior to first administration of IMP)	n.a.	No DLT acc. to protocol
NOA26-028	Nausea during application i.th. Nivolumab	General disorders and administration site conditions	2- Moderate	No	Yes	Ondansetron 4 mg as required s.l.	Yes	Dose not changed	No DLT acc. to protocol
NOA26-028	Dizziness during application i.th. Nivolumab	General disorders and administration site conditions	1- Mild	No	Yes	Just water drinking, non-drug treatment	Yes	Dose not changed	No DLT acc. to protocol

Subject	Description	Term	CTCAE Grading	SAE	Treatment	Description of treatment	Related to IMP	Action	DLT
NOA26-028	Involuntary movements both legs, also involuntary meovemnt both legs during Treatment nivolumab 02.01.2025	General disorders and administration site conditions	1- Mild	No	No	-	Yes	Dose not changed	No DLT acc. to protocol
NOA26-028	Involuntary movement eyelid Right / twitch	General disorders and administration site conditions	1- Mild	No	No	-	Yes	Dose not changed	No DLT acc. to protocol
NOA26-028	Dizziness during application i.th. Nivolumab	General disorders and administration site conditions	1- Mild	No	Yes	Drinking water and half lying position	Yes	Dose not changed	No DLT acc. to protocol
NOA26-028	Muscle twitching for 2 days a day, not daily, for few seconds	Musculoskeletal and connective tissue disorders	1- Mild	No	No	-	Yes	Dose not changed	No DLT acc. to protocol
NOA26-028	Decreased hämatokrit	Blood and lymphatic system disorders	1- Mild	No	No	-	No	Dose not changed	No DLT acc. to protocol
NOA26-028	Increased MCH	Blood and lymphatic system disorders	1- Mild	No	No	-	No	Dose not changed	No DLT acc. to protocol

Subject	Description	Term	CTCAE Grading	SAE	Treatment	Description of treatment	Related to IMP	Action	DLT
NOA26-028	Increased MCV	Blood and lymphatic system disorders	1- Mild	No	No	-	No	Dose not changed	No DLT acc. to protocol
NOA26-028	Increased alk. phosphat	Blood and lymphatic system disorders	1- Mild	No	No	-	No	Dose not changed	No DLT acc. to protocol
NOA26-028	Increased Gamma GT	Blood and lymphatic system disorders	3- Severe	No	No	-	No	Dose not changed	No DLT acc. to protocol
NOA26-028	Nausea (during application of nivolumab)	General disorders and administration site conditions	1- Mild	No	Yes	Drinking water & waiting<br><br>Ondansetron 4mg was given on 17-JAN-2025	Yes	Dose not changed	No DLT acc. to protocol
NOA26-028	Tingling in face and feets	Nervous system disorders	1- Mild	No	No	-	Yes	Dose not changed	No DLT acc. to protocol
NOA26-028	Numbness in the lips and right side of the face	Nervous system disorders	1- Mild	No	No	-	Yes	Dose not changed	No DLT acc. to protocol
NOA26-028	Headache	General disorders and administration site conditions	1- Mild	No	No	-	Yes	Dose not changed	No DLT acc. to protocol

Subject	Description	Term	CTCAE Grading	SAE	Treatment	Description of treatment	Related to IMP	Action	DLT
NOA26-028	Vomiting after application Nivolumab	Gastrointestinal disorders	2- Moderate	No	Yes	Ondansetron 4 mg	Yes	Dose not changed	No DLT acc. to protocol
NOA26-028	Tingling in face (Right lower lip) and both feets	Nervous system disorders	1- Mild	No	No	-	Yes	Dose not changed	No DLT acc. to protocol
NOA26-028	CRP decreased, Infect, most likely respiratory	Blood and lymphatic system disorders	2- Moderate	No	Yes	Amoxicillin 1000 mg	No	Drug withdrawn	No DLT acc. to protocol
NOA26-028	Pain while breathing	Respiratory, thoracic and mediastinal disorders	2- Moderate	No	Yes	Amoxicillin 1000 mg	No	Drug withdrawn	No DLT acc. to protocol
NOA26-028	Bladder infection, cystitis	Renal and urinary disorders	2- Moderate	No	Yes	Pivmecillinam	No	n.a.	No DLT acc. to protocol
NOA26-028	Vomiting after intake amoxicillin	Gastrointestinal disorders	2- Moderate	No	No	-	No	n.a.	No DLT acc. to protocol
NOA26-028	Tingling both feets and sool of feets	Nervous system disorders	1- Mild	No	No	-	Yes	Dose not changed	No DLT acc. to protocol
NOA26-028	Dizziness during application nivolumab	General disorders and administration site conditions	1- Mild	No	No	-	Yes	Dose not changed	No DLT acc. to protocol

Subject	Description	Term	CTCAE Grading	SAE	Treatment	Description of treatment	Related to IMP	Action	DLT
NOA26-028	Nausea during application nivolumab	General disorders and administration site conditions	1- Mild	No	No	-	Yes	Dose not changed	No DLT acc. to protocol
NOA26-028	Tingling in both legs	Nervous system disorders	1- Mild	No	No	-	Yes	Dose not changed	No DLT acc. to protocol
NOA26-028	Nausea	General disorders and administration site conditions	1- Mild	No	No	-	Yes	Dose not changed	No DLT acc. to protocol
NOA26-028	Nausea during application i.th. Nivolumab	Gastrointestinal disorders	2- Moderate	No	No	-	Yes	Dose not changed	No DLT acc. to protocol
NOA26-029	Increased kreatinin	Blood and lymphatic system disorders	1- Mild	No	No	-	n.a. (Date of onset prior to first administration of IMP)	n.a.	No DLT acc. to protocol
NOA26-029	Increased GOT	Blood and lymphatic system disorders	1- Mild	No	No	-	n.a. (Date of onset prior to first administration of IMP)	n.a.	No DLT acc. to protocol

Subject	Description	Term	CTCAE Grading	SAE	Treatment	Description of treatment	Related to IMP	Action	DLT
NOA26-029	Increased GPT	Blood and lymphatic system disorders	2- Moderate	No	No	-	n.a. (Date of onset prior to first administration of IMP)	n.a.	No DLT acc. to protocol
NOA26-029	Lymphocyte count decreased	Blood and lymphatic system disorders	2- Moderate	No	No	-	n.a. (Date of onset prior to first administration of IMP)	n.a.	No DLT acc. to protocol
NOA26-029	Nausea (few days after chemotherapy-Mamma CA)	General disorders and administration site conditions	1- Mild	No	No	-	n.a. (Date of onset prior to first administration of IMP)	n.a.	No DLT acc. to protocol
NOA26-029	Diarrhae	Gastrointestinal disorders	2- Moderate	No	Yes	Loperamid 2 mg as required	Yes	Dose not changed	No DLT acc. to protocol
NOA26-029	Headache	General disorders and administration site conditions	1- Mild	No	No	-	Yes	Dose not changed	No DLT acc. to protocol
NOA26-029	Nausea	Gastrointestinal disorders	2- Moderate	No	Yes	Aprepitant	n.a. (Date of onset prior to first administration of IMP)	Dose not changed	No DLT acc. to protocol

Subject	Description	Term	CTCAE Grading	SAE	Treatment	Description of treatment	Related to IMP	Action	DLT
NOA26-029	Vomiting	General disorders and administration site conditions	2- Moderate	No	Yes	Apreptiant	n.a. (Date of onset prior to first administration of IMP)	Dose not changed	No DLT acc. to protocol
NOA26-029	Tingling in the heels both sides	Nervous system disorders	1- Mild	No	No	-	Yes	Dose not changed	No DLT acc. to protocol
NOA26-029	Weight loss	Metabolism and nutrition disorders	1- Mild	No	No	-	Yes	Dose not changed	No DLT acc. to protocol
NOA26-029	Decreased AP	Blood and lymphatic system disorders	2- Moderate	No	No	-	Yes	Dose not changed	No DLT acc. to protocol
NOA26-029	Decreased bilirubin	Blood and lymphatic system disorders	2- Moderate	No	No	-	Yes	Dose not changed	No DLT acc. to protocol
NOA26-029	Tingling in both feets	Nervous system disorders	1- Mild	No	No	-	Yes	Dose not changed	No DLT acc. to protocol
NOA26-029	Gait uncertainty	Nervous system disorders	1- Mild	No	No	-	Yes	Dose not changed	No DLT acc. to protocol
NOA26-029	Double Vision for short time intermittendly	Eye disorders	1- Mild	No	No	-	Yes	Dose not changed	No DLT acc. to protocol

Subject	Description	Term	CTCAE Grading	SAE	Treatment	Description of treatment	Related to IMP	Action	DLT
NOA26-029	Headache	Nervous system disorders	1- Mild	No	No	-	Yes	Dose not changed	No DLT acc. to protocol
NOA26-029	Chills	General disorders and administration site conditions	1- Mild	No	No	-	Yes	Dose not changed	No DLT acc. to protocol
NOA26-029	Paresis N. Trochlearis	Eye disorders	2- Moderate	No	Yes	Masking of the right or left eye possible for binocular double Vision<br><br>+ cMR recommended	Yes	Dose not changed	No DLT acc. to protocol
NOA26-029	Dizziness	General disorders and administration site conditions	1- Mild	No	No	-	Yes	Dose not changed	No DLT acc. to protocol
NOA26-029	CRP elevation	General disorders and administration site conditions	2- Moderate	Yes	Yes	Admission to our hospital ward<br><br>A focus search was performed<br><br>i.v. fluid	No	Dose not changed	No DLT acc. to protocol
NOA26-029	Fatigue	General disorders and administration site conditions	2- Moderate	No	Yes	Resting, Taking naps, regulated activities	Yes	Dose not changed	No DLT acc. to protocol
