# Supplementary material for: Intrathecal nivolumab in metastatic solid tumors with leptomeningeal disease: dose escalation part of the multicenter IT-PD1/NOA-26 phase 1 trial
Source: Nat Cancer. 2026 Jun 4;7(7):1094–103. doi: 10.1038/s43018-026-01185-4 (PMC13400297; doi:10.1038/s43018-026-01185-4)
Supplement: Supplementary file 3 — Reporting Summary [file 43018_2026_1185_MOESM3_ESM.pdf]

nature portfolio

Corresponding author(s): Ghazaleh Tabatabai

Last updated by author(s): Mar 22, 2026

Reporting Summary

Nature Portfolio wishes to improve the reproducibility of the work that we publish. This form provides structure for consistency and transparency in reporting. For further information on Nature Portfolio policies, see our Editorial Policies and the Editorial Policy Checklist.

Statistics

For all statistical analyses, confirm that the following items are present in the figure legend, table legend, main text, or Methods section.

n/a	Confirmed	
☐	☒	The exact sample size (*n*) for each experimental group/condition, given as a discrete number and unit of measurement
☐	☒	A statement on whether measurements were taken from distinct samples or whether the same sample was measured repeatedly
☒	☐	The statistical test(s) used AND whether they are one- or two-sided<br>*Only common tests should be described solely by name; describe more complex techniques in the Methods section.*
☒	☐	A description of all covariates tested
☒	☐	A description of any assumptions or corrections, such as tests of normality and adjustment for multiple comparisons
☐	☒	A full description of the statistical parameters including central tendency (e.g. means) or other basic estimates (e.g. regression coefficient) AND variation (e.g. standard deviation) or associated estimates of uncertainty (e.g. confidence intervals)
☒	☐	For null hypothesis testing, the test statistic (e.g. F, t, r) with confidence intervals, effect sizes, degrees of freedom and P value noted<br>*Give P values as exact values whenever suitable.*
☒	☐	For Bayesian analysis, information on the choice of priors and Markov chain Monte Carlo settings
☒	☐	For hierarchical and complex designs, identification of the appropriate level for tests and full reporting of outcomes
☒	☐	Estimates of effect sizes (e.g. Cohen's d, Pearson's r), indicating how they were calculated

Our web collection on statistics for biologists contains articles on many of the points above.

Software and code

Policy information about availability of computer code

Data collection	Data were collected during trial visits according to the protocol and entered in eCRFs by qualified study personal. The data were exported to statistical software.
Data analysis	The statistical analysis was descriptively. Dose levels pooled due to the small number of subjects per level (n=3 or n=6). For continuous measurements, medians and interquartile ranges (IQR) were used, for categorical variables absolute frequencies and percentages. Censored data were presented using Swimmer plots, Kaplan Meier plots and related parameters (median survival, survival after 6 and 12 and 18 months including two-sided 95% confidence limits). Quality of Life and cognitive scales were displayed using line graphs including two-sided 95% confidence limits. We used SPSS for Windows, release 29 and R release 4.1.2

For manuscripts utilizing custom algorithms or software that are central to the research but not yet described in published literature, software must be made available to editors and reviewers. We strongly encourage code deposition in a community repository (e.g. GitHub). See the Nature Portfolio guidelines for submitting code & software for further information.

Data

Policy information about availability of data

All manuscripts must include a data availability statement. This statement should provide the following information, where applicable:

- Accession codes, unique identifiers, or web links for publicly available datasets
- A description of any restrictions on data availability
- For clinical datasets or third party data, please ensure that the statement adheres to our policy

All data included in the paper have been generated as part of the clinical trial and are subject to patient confidentiality. Thus, pseudonymized / de-identified clinical data can be made available per request.
All requests will be reviewed by the Institutional Review Board of the University Hospital Tübingen and the Legal Department of the University Hospital Tübingen.
Any requests should be directly addressed to the corresponding author ghazaleh.tabatabai@uni-tuebingen.de.
These requests will be processed within a timeframe of 3-6 months.
Any patient-related data that is not included in this publication has been generated as part of the NOA-26 clinical trial and are protected by patient confidentiality.
All de-identified clinical data will be made available with the final reporting of the NOA-26 clinical trial.
All patients are referred to in the manuscript with a code (NOA-26-XXX).
This code is not the study ID of these patients but a code that has been used for this manuscript and this publication.
The full study protocol is available in the Supplementary Information.
A complete set of source data for Table 1, Figure 3, Figure 4, Extended Table 1 have been provided as Source Data Files in the Supplementary Information.
A full set of clinical data (all adverse events, all treatment-related adverse events, individual patient summaries) is available in Extended Tables and in Supplementary Information.

Research involving human participants, their data, or biological material

Policy information about studies with human participants or human data. See also policy information about sex, gender (identity/presentation), and sexual orientation and race, ethnicity and racism.

Reporting on sex and gender	The NOA-26 trial was open to any sex and / or gender. In the trial, information on sex and/or gender were mandatory requirements and these information were determined based on self-report.
Reporting on race, ethnicity, or other socially relevant groupings	The NOA-26 trial was open to all ethnicities, race, socially groups. These information have not been collected.
Population characteristics	The study population has been defined by the inclusion and exclusion criteria. These criteria are included in the Study Protocol that has been provided in Supplementary Information. Furthermore, population characteristics at baseline visit have been summarized in Table 1 of the manuscript.
Recruitment	Participants were recruited at all clinical sites in Germany according to pre-defined inclusion and exclusion criteria. Eligible patients were identified by study investigators and enrolled following provision of informed consent. Due to the specific nature of the disease and its very high unmet therapeutic need, rarity and often unfavorable course, the study population was necessarily selected. Furthermore, this is a phase 1 trial. These factors limit generalizability to broader patient populations at this stage of clinical development.
Ethics oversight	This study has been authorized and the study design complied with all relevant regulations regarding the use of human study participants and was conducted in accordance with the criteria set by the Declaration of Helsinki.<br>The first submission to the Institutional Review Board of the University Hospital Tübingen and to the Paul Ehrlich Institute (PEI) was in May 2021.<br>The study received approval by the PEI in August 2021, and in September 2021 by the Institutional Review Board of the University Hospital Tübingen.<br>The first substantial amendment was submitted in July 2022 and approved.<br>The second substantial amendment was submitted in September 2023.<br>NOA-26 trial has been transferred based on the Clinical Trials Regulation (CTR) in Europe, and the CTIS transition has been completed.<br>NOA-26 runs under the CTR since 24 May 2024. The first substantial amendment after CTIS transition was implemented in November 2024.<br>The current version of the NOA-26 Study protocol is Version 5.1, date 15th November 2024 (Supplementary information).

Note that full information on the approval of the study protocol must also be provided in the manuscript.

Field-specific reporting

Please select the one below that is the best fit for your research. If you are not sure, read the appropriate sections before making your selection.

☒ Life sciences ☐ Behavioural & social sciences ☐ Ecological, evolutionary & environmental sciences

For a reference copy of the document with all sections, see nature.com/documents/nr-reporting-summary-flat.pdf

Life sciences study design

All studies must disclose on these points even when the disclosure is negative.

Sample size	The Statistical Analyses for the NOA-26 trial outlined in Chapter #9 of the enclosed Study Protocol. Chapter 9.1.1 gives an overview of sample size and power calculations as follows: "This trial includes a minimum of 32 evaluable patients (12 Part I, 20 Part II) and a maximum of 49 evaluable patients (24 Part I, 25 Part II). Between 12 and 24 patients will be included in the dose finding phase Part I with four doses (20, 30, 40, 50 mg) and 20 evaluable patients will be included in the expansion phase using the MTD identified in the 3+3 phase. With 20 evaluable patients, it can be shown, that DLT is smaller than 33% assuming a true DLT of maximal 7% (exact binomial test, type 1 error = 0.025 one-sided, power = 80%, H0: DLT = 33%, H1: DLT < 33%, assumed alternative: DLT ≤ 7%)."
Data exclusions	No data were excluded from the analyses. Participants who did not meet screening criteria or who withdrew during the trial are separately identified, with their status clearly defined in the corresponding tables and figures.
Replication	This is a Dose Escalation Phase of an Investigator-initiated Phase 1 trial, and this has been performed according to a 3+3 design. The DSMB reviewed all safety data after each cohort and approved the continuation to the next dose level. In the last dose level (and as outlined in Figure 2), three patient were enrolled without any DLT. To confirm this, three additional patients have been enrolled per request of the DSMB. These additional three patients did not experience any DLT, and the safety signal has been confirmed. Since this is a phase 1 trial, no further replications were foreseen within the scope of this study. The currently ongoing Expansion Phase (Part B) with a fixed dose of 50 mg will investigate and confirm safety of treatment with 50 mg nivolumab IT.
Randomization	This is a prospective, multicenter, single arm investigator-initiated phase 1 trial.
Blinding	This is a prospective, multicenter, single arm investigator-initiated phase 1 trial.

Reporting for specific materials, systems and methods

We require information from authors about some types of materials, experimental systems and methods used in many studies. Here, indicate whether each material, system or method listed is relevant to your study. If you are not sure if a list item applies to your research, read the appropriate section before selecting a response.

Materials & experimental systems

n/a	Involved in the study	
☒	☐	Antibodies
☒	☐	Eukaryotic cell lines
☒	☐	Palaeontology and archaeology
☒	☐	Animals and other organisms
☐	☒	Clinical data
☒	☐	Dual use research of concern
☒	☐	Plants

Methods

n/a	Involved in the study	
☒	☐	ChIP-seq
☒	☐	Flow cytometry
☒	☐	MRI-based neuroimaging

Clinical data

Policy information about clinical studies
All manuscripts should comply with the ICMJE guidelines for publication of clinical research and a completed CONSORT checklist must be included with all submissions.

Clinical trial registration	NCT05112549
Study protocol	The full study protocol is enclosed as supplementary information.
Data collection	Data were collected at participating clinical sites in Germany in accordance with the study protocol following written informed consent. Recruitment was initiated in October 2021 , first patient enrolled in December 2021 and data collection continued until the DSMB received the final Safety Reporting on Part A on 22 April 2025. Consequently, the data cutoff of this manuscript is 22 April 2025. The Expansion Phase (Part B) of the NOA-26 trial is ongoing since May 2025.
Outcomes	The Study Objectives and Outcomes are outlined in the enclosed Study protocol in chapter #2:<br>Primary Objective and Endpoint The primary objective was the assessment of the maximum tolerable dose and safety of intrathecal (IT) nivolumab, as definded as dose limiting toxicity (DLT). The trial has been monitored by an independent Data and Safety Monitoring Committee who evaluated the study after each cohort and decided on proceeding to the next dose level.<br><br>Dose Limiting Toxicities were defined as following:<br>• CTCAE grade 4 or above Adverse Events related to the IMP.<br>• Neurological CTCAE grade 2 and 3 Adverse Events related to the IMP that have a recommendation of permanently discontinuation of immunotherapies according to the “NCCN Guideline on Management of Checkpoint Inhibitor related Toxicities” (NCCN, 2021a), see also Appendix III (section 14.3). The AE assessment included clinical and imaging signs (per LANO, see Appendices I), imaging was performed as per standard of care. Yet, if any of the above- mentioned clinical features occurred, MR imaging was be performed

immediately to investigate the following conditions:
• Increased subarachnoid or ventricular nodules in the brain/spine
• Worsening of leptomeningeal linear enhancement in the brain/spine
• Worsening of hydrocephalus in the brain
• Progression of metastases in the CNS parenchyma (if patient had metastases in the CNS parenchyma at trial entry) The assessment of the primary objectives was be accomplished by clinical assessements, documentation of Adverse Events and grading according to the Common Terminology Criteria for Adverse Events (CTCAE V5.0) from time of signing the Informed Consent until the end of the last Follow-up 4 after last dose within the scope of the clinical study. The safety endpoints will be assessed by a review of Adverse Events and serious Adverse Events.
Secondary Objectives and Endpoints The secondary endpoint is overall survival defined as the time interval from the date of first study administration to the date of death or last contact. Overall survival was analyzed by follow up visits during the clinical trial according to the protocol. They are presented using Swimmer plots, Kaplan Meier plots and related parameters (median survival, percentages of survival (product limit estimate) after 6 and 12 and 18 months including two-sided 95% confidence limits).

Exploratory Objectives The exploratory objectives allowed translational assessments. These include Patient-reported outcome as assessed by EORTC QLQ-C30/EORTC QLQ-BN20, Distress thermometer (Mehnert, M¸ller, Lehmann, & Koch, 2006), Neurocognitive assessments as assessed by Mini Mental Status (MMSE) Score; Montreal Cognitive Assessment (MoCa) Score during intrathecal applications.
The patient-reported outcomes were assessed per protocol during scheduled visits.
Quality of Life and cognitive scales were analyzed and displayed using line graphs including two-sided 95% confidence limits. In these figures, for each time point the numbers of patients under observation is given. These numbers are presented in an additional table.

Plants

Seed stocks

n/a

Novel plant genotypes

n/a

Authentication

n/a
