# Supplementary material for: Intrathecal nivolumab in metastatic solid tumors with leptomeningeal disease: dose escalation part of the multicenter IT-PD1/NOA-26 phase 1 trial
Source: Nat Cancer. 2026 Jun 4;7(7):1094–103. doi: 10.1038/s43018-026-01185-4 (PMC13400297; doi:10.1038/s43018-026-01185-4)
Supplement: Supplementary file 4 — Study protocol. [file 43018_2026_1185_MOESM4_ESM.pdf]

I. Title Page

Intrathecal application of PD-1 antibody in metastatic solid tumors with leptomeningeal disease

Short Title of Clinical Trial	IT-PD1
NOA-Number	NOA-26
Protocol Version	V5.1
Date of Protocol	15 November 2024
EudraCT-Number	2021-001795-42
EU CT Nr	2024-514068-14-00
Clinical gov	NCT05112549
Phase	I
Sponsor	University Hospital Tuebingen,<br><br>Geissweg 3<br>72076 Tuebingen<br>Germany
Investigational Medicinal Product	Nivolumab
Comparator	n/a

CONFIDENTIAL This protocol contains confidential information and is intended solely for the guidance of clinical investigation. This protocol may not be disclosed to parties not associated with the clinical investigation or used for any purpose without the prior written consent of the coordinating Investigator.

II. Table of Contents

I. Title Page 1

II. Table of Contents 2

II a) List of Tables 6

II b) List of Figures 6

III Signature Page 7

IV. Contacts 9

V. Abbreviations 10

VI. Synopsis 12

1. Introduction 21

1.1. Intrathecal therapy of patients with leptomeningeal disease 23

1.2. Ongoing phase I clinical trial at MD Anderson Cancer Center in metastatic melanoma and LMD 23

1.3. Case report (55-year-old, metastatic melanoma with LMD) from the University Hospital Tübingen 24

1.4. Benefit / Risk Assessment 26

1.5. Advisory Committes 31

1.5.1. Data and Safety Monitoring Board (DSMB) 31

2. Study Objectives 32

2.1. Primary Objective and Endpoint 32

2.2. Secondary Objectives and Endpoints 33

2.3. Exploratory Objectives 33

3. Study Design 34

3.1. Study Duration and Schedule 39

3.2. End of Study 40

4. Study Population 41

4.1. General Criteria for Subject Selection 41

4.1.1. Inclusion Criteria 41

4.1.2. Exclusion Criteria 44
5. Requirements for Trial Site and Investigator 46
6. General Information on the Investigational Medical Product 47
6.1. Manufacturing of the Investigational Medicinal Product 47
6.2. Labelling of the Investigational Medicinal Product 48
6.3. Storage of the Investigational Medicinal Product 48
6.4. Drug Accountability, Therapy Compliance and Disposal 48
6.5. Dose Schedule 49
6.6. Dose Modification and DLT (Dose Limiting Toxicities) 52
6.6.1. Dose Modification Part I: 54
6.6.2. Dose Modification Part II 54
6.7. Special warnings and precautions for use of Nivolumab (see SmPC) Monotherapy 54
7. Study Procedures and Examination Method 56
7.1. Study Entry 57
7.1.1. Patient’s Informed Consent 57
7.1.2. Screening 57
7.1.3. Enrollment 59
7.1.4. Concomitant Medication and Treatments 60
7.1.5. Prohibited Concomitant Medications and Treatments 61
7.1.6. Interaction with other medicinal products and other forms of interaction 61
7.1.6.1. Other forms of interaction Systemic immunosuppression 61
7.2. Treatment Phase 62
7.2.1. Description Treatment Visits 1-6 62
7.3. Safety Visit 1 and 2 for Subjects 64
7.4. Follow-up 1-4 and Long-time Follow-up 65
7.5. Assessment of Efficacy and Safety 66
7.6. Premature termination of clinical trial for a trial subject 67

7.7. Premature closure of a trial site 68
7.8. Premature termination of the trial 69
7.9. Plan for Treatment or Care after End of Study 69
8. Quality control and Quality assurance 70
8.1. Risk-based approach 70
8.2. Monitoring 70
8.3. Audits/ Inspections 70
8.4. Documentation: Collection, Handling, Storage and Archiving of Data 71
8.4.1. Case Report Form 71
8.4.2. Source Data 71
8.4.3. Data Handling 72
8.4.4. Storage and Archiving of Data 72
9. Statistical Analyses 73
9.1. Study Population Definition 73
9.1.1. Sample Size and Power Consideration 73
9.2. Analysis Primary Variables 73
9.3. Analysis Secondary Variables 73
9.4. Safety Interim Analysis 74
9.5. Stopping Rules 74
9.6. Drop-out 74
9.7. Biometric Report 75
10. Safety 76
10.1. Definition of Adverse Events, Serious Adverse Events and Side Effects 76
10.1.1. Adverse Events 76
10.1.2. Serious Adverse Event and Serious Adverse Reaction 77
10.1.3. Adverse Drug Reaction 77
10.1.4. Unexpected Adverse Drug Reaction 77
10.2. Reporting of Adverse Events/Serious Adverse Events 78

10.2.1. Period of Observation and Documentation 78
10.2.2. Documentation and Reporting of AE/SAE by the Investigator 78
10.2.3. Assessment of Severity and Causality 79
10.2.4. Relationship of the AEs to the Investigational Medicinal Product 79
10.2.5. Outcome of the Adverse Reactions and actions taken 80
10.2.6. Sponsors Assessment of the SAEs 81
10.2.7. Follow-up of Initial Report 81
10.2.8. Exception of reporting 81
10.2.9. Suspected Unexpected Serious Adverse Reaction (SUSAR) 82
10.2.10. Expedited Reporting to the Regulatory Authorities 82
10.2.11. Examination and Report of Changes in the Risk to Benefit Ratio 83
10.2.12 Reporting to Data and Safety Monitoring Board 83
10.2.13 Report to the Investigator 84
10.3. Annual Safety Report 84
10.4. Deviations from the Protocol and serious breaches 84
10.5. Reporting of Pregnancy 85
11. Regulatory Consideration 87
11.1. Ethical Conduct of Clinical Study 87
11.1.1. Good Clinical Practice, Declaration of Helsinki and legal Provision 87
11.2. Subject Information and Informed Consent 87
11.3. Insurance 87
11.4. Confidentiality 88
11.5. Responsibility of the Investigator 91
11.6. Registration of the Trial 91
11.7. Reporting obligations (Sponsor) according to the EU-CTR 536/2014 91
11.8. Approval of Protocol and Subsequent Amendments 93
12. Publications 94
12.1. Reports 94

12.2. Publication 94
13. Financing 95
14. Appendix 96
14.1. Appendix I: LANO (Le Rhun et al., 2019) 96
14.2. Appendix II: Reference safety information for assessment of expectedness of serious adverse reactions (RSI) 98
14.3. Appendix III: NCCN Guidelines (NCCN, 2021a) 99
15. Literature 106

II a) List of Tables

Table 1: Table of Events 18
Table 2 Dosing information for the case report 25
Table 3: Study Timelines 39
Table 4 Temporary suspension 53
Table 5: Serious Adverse Reactions (SAR) for the IMP considered expected for safety reporting purposes (n = number of subjects who have experienced the SAR; SOC = system organ class; t.b.d. = to be determined.) 98

II b) List of Figures

Figure 1 3x3 design with 4 cohorts 36
Figure 2 Overall Study Design for Part I 37
Figure 3 Overall Study Design for Part II 38
Figure 4 staggered exposure of subjects for each cohort 51

III Signature Page

The present trial protocol was subject to critical review and has been approved in the present version by the persons signed.

Sponsor:

The University Hospital Tuebingen is sponsor for the purpose of Art. 2 (2) 14.

Regulation (EU) No 536/2014 with complementary regulations. The internal responsibility to comply with the obligations of the sponsor in terms of these regulations stays with Prof. Dr. Ghazaleh Tabatabai

Date: 19 NOV 2024 Signature:________________________

Prof. Dr. med. Ghazaleh Tabatabai:

Function: Delegated sponsor and Coordinating Investigator

Declaration of the Principal Investigator

By my signature, I agree to supervise personally the conduct of this study at my study site and to ensure its conduct is in compliance with the protocol, informed consent, the national laws, the ICH Good Clinical Practices Guidelines and the Declaration of Helsinki. I will train the involved personal accordingly.

Date: ____________ Signature:___________________________________

Name:

Function: Principal Investigator

Adress of the Study Center: ____________________________________

____

____

IV. Contacts

**Sponsor**	Universitätsklinikum Tuebingen<br>Geissweg 3<br>72076 Tuebingen
**Coordinating Investigator**	Ghazaleh Tabatabai, Prof. Dr. med. Dr. rer. nat.<br>Department of Neurology and Interdisciplinary Neuro-Oncology<br>Hoppe-Seyler-Strasse 3<br>+49 (0) 7071 – 2985018<br>+49 (0) 7071 – 294608<br>ghazaleh.tabatabai@uni-tuebingen.de
**Biometrician**	Germany Institute for Clinical Epidemiology and Applied Biostatistics (IKEAB)<br>Silcherstrasse 5<br>72076 Tuebingen<br>Germany<br>Phone: +49 (0)7071 29-85072<br>Fax: +49 (0)7071 29-86829
**Data management**	Institute for Clinical Epidemiology and Applied Biometry (IKEAB)<br>Silcherstrasse 5<br>72076 Tuebingen<br>Germany<br>Phone: +49 (0)7071 29-85072<br>Fax: +49 (0)7071 29-5075
**Project management**	Zentrum fur Klinische Studien Tubingen (ZKS)<br>Röntgenweg 9<br>72076 Tuebingen, Germany<br>Phone: +49 7071 29-85273<br>Fax:+49 7071 29-25080<br>e-mail: zks-pm@med.uni-tuebingen.de
**Monitoring**	Zentrum fur Klinische Studien Tubingen (ZKS)<br>Röntgenweg 9<br>72076 Tuebingen, Germany<br>Phone: +49 7071 29-85635<br>Fax:+49 7071 29-25080<br>e-mail: zks-mo@med.uni-tuebingen.de
**SAE-Management**	Zentrum fur Klinische Studien Tubingen (ZKS)<br>Röntgenweg 9<br>72079 Tuebingen, Germany<br>Phone: +49 7071 29-85545<br>e-mail: zks-pv@med.uni-tuebingen.de
**SAE-Reporting**	**Fax: +49 7071 29 25205**

V. Abbreviations

ADR	Adverse Drug Reaction
AE	Adverse Event
AMG	German Drug Law (Deutsches Arzneimittelgesetz)
MPG	German Medical Device Law (Deutsches Medizinproduktegesetz)
BfArM	Bundesinstitut für Arzneimittel und Medizinprodukte
BMBF	Bundesministerium für Bildung und Forschung
cfDNA	cell free DNA
CI	Coordinating Investigator
CRF	Case Report Form
CSF	Cerebrospinal fluid
CTCAE	Common Terminology Criteria for Adverse Events
CNS	central nervous system
DBL	Data Base Lock
DLT	Dose Limiting Toxicities
DGN	Deutsche Gesellschaft für Neurologie
DSMB	Data and Safety Monitoring Board
EC	Ethics Committee
ECG	Electrocardiogram
EORTC QLQ	European Organisation for Research and Treatment of Cancer Core Quality of Life Questionnaire
FCBP	Female Childbearing Potential
FSI	First Subject In
FSH	Follicle Stimulating Hormone
GCP	Good Clinical Practice
GCP-V	Good Clinical Practice Ordinance (GCP-Verordnung)
GMP	Good Manufacturing Practice
IB	Investigator’s Brochure
IC	Informed Consent
ICH	International Conference on Harmonization of Technical Requirements for Registration of Pharmaceuticals for Human Use
IIT	Investigator Initiated Trial

IMP	Investigational Medicinal Product
IMPD	Investigational Medicinal Product Dossier
ISF	Investigator Site File
ITT	Intention to Treat
KPS	Karnofsky performance score
LANO	Leptomeningeal Assessment in Neuro-Oncology
LKP	Leiter der Klinischen Prüfung (Coordinating Investigator)
LMD	Leptmeningeal disease
LP	Lumbal Puncture
LSI	Last Subject In
LSO	Last Subject Out
MMSE	Mini Mental Status Score
MoCa	Montreal Cognitive Assessment Score
NANO	Neurologic Assessment in Neuro-Oncology
MRT	Magnetic Resonance Imaging
MTD	Maximum Tolerated Dose
n.a.	Not applicable
NOA	Neuroonkologischer Arbeitskreis
PBMC	Peripheral Blood Mononuclear Cell
PCR	Polymerase Chain Reaction
PD-1	Programmed Cell Death Protein 1
PD-L1	Programmed Death-Ligand 1
PEI	Paul-Ehrlich-Institut
RANO	ResponseAssessment in NeuroOncology
SAB	Scientific Advisory Board
SAE	Serious Adverse Event
SmPC	Summary of Product Characteristics (deutsch: Fachinformation)
SDV	Source Data Verification
SUSAR	Suspected Unexpected Serious Adverse Reaction
TMF	Trial Master File

VI. Synopsis

Sponsor	University Hospital Tübingen
Title	Intrathecal application of PD-1 antibody in metastatic solid tumors with leptomeningeal disease
Short Title/Study Code	NOA-26, IT-PD1
EudraCT-Number<br><br>EU CT Nr	2021-00179<br><br>2024-514068-14-005-42
Coordinating Investigator	Ghazaleh Tabatabai<br>Department of Neurology & Interdisciplinary Neuro-Oncology<br>University Hospital Tübingen<br>Comprehensive Cancer Center Tübingen-Stuttgart
Study Design	Prospective, interventional, multicenter, open label, phase 1
Number of Patients	**Part I: 12 (24) subjects**<br><br>In Part I a maximum of 24 (minimum 12) subjects will receive an intrathecal Nivolumab treatment. To reflect the primary objective, Part I is designed as a dose - escalation trial with a “3 + 3 design”. There will be 4 cohorts, each with a fixed predefined dosage (20 mg, 30 mg, 40 mg, 50 mg).<br><br>The DSMB will be conducted after the first three (or six) patients of each cohort have completed Safety Visit 1. For each cohort there will be an interim safety analysis and all Adverse Events will be assessed by the data safety monitoring board (DSMB) as outlined in the DSMB Charta. At the end of Part I of this clinical trial the maximal tolerated dosage shall be determined. This will be the fix dose for Part II.<br><br>**Part II: 20 (25) subjects**<br><br>Depending on the results of Part I the fix dosage for Part II will be 20 mg, 30 mg, 40 mg or 50 mg.

	All patients enrolled into the trial and receiving at least one intrathecal application of PD-1 will belong to the Intention-to-treat (ITT) population.
Patient Population	Intrathecal application of PD-1 antibody in metastatic solid tumors with leptomeningeal disease of solid tumors.
Length of study/ Time Lines	Total trial duration: 6 years<br>Duration for individual patient:<br>• Screening phase: up to 14 days<br>• Study treatment phase: 10 weeks, two additional Safety Visits 7 days after the 3th or 6th dose<br>• Follow-up phase: 4 months<br>FSI (First Subject In): Q4.2021<br>LSI (Last Subject In): Q1.2026<br>LSO (Last Subject Out): Q3.2026<br>DBL (Data Base Lock): Q4.2026<br>Statistical Analyses Completed: Q1.2027<br>Trial reported completed: Q3.2027
Aim of the Study	To determine the safety of intrathecal (IT) PD-1 antibody
Objectives/Endpoints	Primary Objective/Endpoint<br>• To assess the maximum tolerable dose and safety of intrathecal (IT) PD-1 antibody administration<br>Secondary Objective/Endpoints<br>• Overall survival<br>Exploratory Objective<br>• Effects of IT PD-1 on cytokines and immune cells in CSF and peripheral blood

	• Longitudinal changes of cfDNA in CSF with IT PD-1 treatment<br>• Quality of life, patient-reported outcome
Main Inclusion Criteria<br><br>See section 4.1.1	• Signed informed consent<br>• Ability to understand and voluntarily sign an informed consent form<br>• Ability to adhere to the study visit schedule and other protocol requiremends<br>• Patients with Karnofsky performance score > 50%<br>• Non-adherent and/or adherent leptomeningeal disease in patients with metastatic solid tumors based on assessments in CSF analysis and MRI<br>• Patient has already completed radiation therapy since > 2 weeks or does not have any clinical indication/option for radiation therapy<br>• Ability to undergo intrathecal therapy via an intraventricular catheter
Main Exclusion Criteria<br><br>See section 4.1.2	• The underlying primary tumor has not a registered and authorized indication in the European Union for intravenous treatment with Nivolumab, pembrolizumab or atezolizumab. The registered solid tumors are, i.e. melanoma, non-small cell lung cancer (NSCLC), Malignant pleural mesothelioma (MPM), renal cell carcinoma (RCC), Classical Hodgkin lymphoma (cHL), squamous cell cancer of the head and neck (SCCHN), urothelial carcinoma, muscle invasive urothelial carcinoma (MIUC), colorectal cancer (CRC) with Mismatch repair deficient (dMMR) or microsatellite instability-high (MSI-H), esophageal squamous cell carcinoma (ESCC), esophageal cancer (EC) or gastro-oesophageal junction cancer (GEJC), gastro-

	oesophageal junction (GEJ) or oesophageal adenocarcinoma, triple-negative breast carcinoma. In addition, leptomeningeal disease of solid tumors with a high tumor mutational burden is also eligible<br>• Patient who cannot undergo MRI
Statistics, Safety Variables and Stopping Rules	This trial includes a minimum of 32 evaluable patients (12 Part I, 20 Part II) and a maximum of 49 evaluable patients (24 Part I, 25 Part II). Between 12 and 24 patients will be included in the dose finding phase Part I with four doses (20, 30, 40, 50 mg), and 20 evaluable patients will be included in the expansion phase using the MTD identified during the 3+3 phase. With 20 evaluable patients it can be shown that DLT is smaller than 33% assuming a true DLT of maximal 7% (exact binomial test, type 1 error = 0.025 one-sided, power = 80%, H0: DLT = 33%, H1: DLT < 33%, assumed alternative: DLT ≤ 7%).<br><br>The treatment for a subject will be discontinued if (among other things) any of the following criteria applies:<br>• Clinical and MRI signs of tumor progression<br>• CTCAE grade 4 or above Adverse Events related to the IMP<br>• Neurological CTCAE grade 2 and 3 Adverse Events related to the IMP that have a recommendation of permanently discontinuation of immunotherapies according to the “NCCN Guideline on Management of Checkpoint Inhibitor related Toxicities” (NCCN, 2021a), see also Appendix III<br>• Decision of patient, i.e. withdrawal of informed consent. If a patient retracts the consent for study treatment, he/she will be asked to stay in the Follow-up to ensure clinical monitoring of patient and Follow-up data. If patient is not willing to do so, he/she will be completely excluded from the trial upon consent withdrawal

Study Intervention/ Study Medication	Intrathecal application of the PD-1 antibody Nivolumab. The minimal dosage is 20 mg, the maximal dosage will be 50 mg.<br><br>Part I (dose escalation) is a dose cohort design with four cohorts (20 mg, 30 mg, 40 mg, 50 mg). For each cohort 3 or 6 subjects are enrolled into a given dose cohort. In case of completion Safety Visit 1 without any DLT the trial proceeds to enroll additional subjects into the next higher dose cohort. If one subject develops a DLT (Dose Limiting Toxicities) at a specific dose, three additional subjects are enrolled into that same dose cohort. The DSMB will monitor the study and the safety aspects of the trial for each cohort.<br><br>Dose Limiting Toxicities are defined as following:<br><br>• CTCAE grade 4 or above: Adverse Events related to the IMP<br>• Neurological CTCAE grade 2 and 3: Adverse Events related to the IMP that have a recommendation of permanently discontinuation of immunotherapies according to the “NCCN Guideline on Management of Checkpoint Inhibitor related Toxicities” (NCCN, 2021a), see also Appendix III<br><br>Development of DLTs in more than 1 of 6 subjects in a specific dose cohort suggests that the MTD (Maximum Tolerated Dose) has been exceeded, and further dose escalation is not pursued. On each dose level, exposure of subjects follows a staggered approach by an interval of one week for the first three subjects. At the end of Part I the DSMB will make a recommendation to determine the maximal fix dose for Part II.
Description of the Investigational Medicinal Product	Nivolumab is a registered PD-1 antibody for the treatment of metastatic solid tumors (as outlined in Fachinformation).

GCP-compliance	The study will be conducted according to the ICH-GCP guidelines
Financing	“Health First: Translationale Forschung”<br>Ministry of Science and of Arts, Baden Württemberg

Table 1: Table of Events

Events	Screening period [a]	IMP Visit (V1-3) [b]	Safety Visit 1 [c]	IMP Visit (4-6) [b]	Safety Visit 2 [c]	Follow-up (1-4), [d]	Continuation of IT treatment until progression (outside of trial) [e]	Long time FU outside of the trial [f]
Study Entry								
Informed Consent (IC), secondary IC is optional	X							
Tumor block/unstained slices[1]	X							
Inclusion/Exclusion Criteria	X							
Disease Diagnosis[2]	X							
Prior Therapies	X							
Demographics	X							
Medical History[3]	X							
Safety Assessments								
Adverse Events	after signing the IC the AE assessment will start and be performed until Follow-up 4 after the last IMP dose within the trial							
Concomitant Medications	Conc. medication will be documented from signing IC until 4 weeks after discontinuation from treatment.							
Physical Exam	X	X	X	X	X			
Height/weight[4]	X	X	X	X	X			
Vital Signs[5]	X	X	X	X	X			
KPS	X	X	X	X	X			
12 –lead EKG	X							
Hematology[6]	X	X	X	X	X			
Chemistry[7]	X	X	X	X	X			
Coagulation[8]	X	X	X	X	X			
TSH, T4, Cortisol	X	X	X	X	X			
HBV, HCV and HIV	X							
Urinanalyse[9]	X	X	X	X	X			
Covid-19 test (optional)	X	X	X	X	X			
Pregnancy test[10]	X	X	X	X	X			
CSF[11]	X	X	X	X	X	(X)		
CSF biomarker[12]	X	X	X	X	X	(X)		

**Events**	**Screening period** [a]	**IMP Visit (V1-3)** [b]	**Safety Visit 1** [c]	**IMP Visit (4-6)** [b]	**Safety Visit 2** [c]	**Follow-up (1-4),** [d]	**Continuation of IT treatment until progression (outside of trial)** [e]	**Long time FU outside of the trial** [f]
Peripheral blood (PBMC)[13]	X	X	X	X	X	(X)		
i.th. IMP Dose		X		X				
Efficacy assessment								
NANO scale[14]	X	X	X	X	X	X		
MMSE[15]		X	X	X	X	X		
MoCa[16]		X	X	X	X	X		
Alllodynia/pain sensation monitoring (medical history)[17]	X	X	X	X	X	X		
Sympathetic skin reaction[17]	X				X			
Optional: QST[17]	X				X			
MRI[18]	X		X		X	X		
LANO assessment[19]	X		X		X	X		
RANO assesment[20]	X		X		X	X		
QoL EORTC[21]	X	X	X	X	X	X		
Survival status[f]								x

[a] screening will be performed -14 up to -1 days prior start of the first dose (V1). Hematology, chemistry coagulation at screening should be repeated within 7 days prior first dose, if done earlier

[b] IT Nivolumab administration at IMP Visit 1-3 and Visit 4-6 will be performed every 14 days (±3 days) via an intraventricular reservoir

[c] Safety Visit 1 and 2 will be performed 7 days (±3 days) after 3th and 6th dose within the study. In case of progress, or unacceptable toxicity or withdrawn, the Safety Visit 1 (after 1th ≥ 3th dose) or 2 (after 4th ≥ 6th dose) will be performed after diagnosis. For drop-out see chapter "drop out" and "study design"

[d] Follow-up 1-4 will be performed every 4 weeks (±3 days) after last dose

[e] Subjects with stable disease will continue with intrathecal Nivolumab until progression outside of this trial every 2 weeks

[f] After end of study of subject (after Follow-up 4), the subject can be followed up every 6 months for survival status, progression or further therapy for 1 year (long-time Follow-up by telephone)

[1] Paraffin tumor block or unstained slices (ca. 20-30) from the primary tumor or metastases outside the CNS, this slides/block is optional at the timepoint of inclusion and will be shipped during the trial and are NOT required prior to enrolment

[2] Diagnosis of the underlying primary solid tumor, tumor type, histology. Optional: date of planned intraventricular catheter implantation

[3] Previous chemotherapy and radiotherapy treatments, current status of systemic disease, presence of parenchymal metastases in the brain and spinal cord

[4] Weight and height (height only at screening)

[5] Blood pressure, heart rate and temperature

[6] Hemoglobin, hematrocit, eryrocytes/RBC (abs.), platelets (abs.), leucocyten/WBC (abs.), neutrophils/ANC (abs.), esoinophils (abs.), basophils (abs.), monocytes (abs.), lymphocytes/ALC (abs.)

[7] Albumin, sodium, potassium, calcium, magnesium, chloride, glucose, alkaline phosphatase, ALT, AST, bilirubin (total + direct), creatinine; urea

[8] Prothrombin time, partial thromboplastin time, INR

[9] Dipstick

[10] Serum pregnancy test for female study participants of childbearing potential must be done within 72 hours prior to the 1st administration of IT Nivolumab until last Safety Visit

[11] Cell count, atypical (tumor) cells, glucose, protein, IL-6, lactate, sample collection until last Safety Visit and optional at the first Follow-up

[12] Collection of biomarker in an extra tube, for further sample processing and storage see laboratory manual. Sample is sent to the sponsor at regular intervals. Sample collection until last Safety Visit and optional at the first Follow-up

[13] For further sample processing and storage see laboratory manual, sample is sent to the sponsor at regular intervals. Sample collection until last Safety Visit and optional at the first Follow-up. For Part I, the PBMC sample will be collected only at the lead center. For Part II, the PBMC sample will be taken at all sites.

[14] NANO (Nayak et al., 2017)

[15] Mini Mental Status (MMSE) Score

[16] Montreal Cognitive Assessment (MoCa) Score

[17] The occurrence of allodynia and increased pain sensation will be monitored clinically (as integral part of the medical history) and by the following neurological examinations: The assessment of sympathetic skin response (per standard of care) is necessary before the start of intrathecal Nivolumab and after 6 applications. Optional and only if applicable and available at the stuy center, a quantitative sensory testing (QST) can be performed for those sites with available QST equipment.

[18] Cerebral and spinal MRI should be done within 14 days prior first IT administration of Nivolumab and as part of the Safety Visits (+/-7 days) according to standard of care as well as during the Follow-up according to the clinical routine every 12 weeks

[19] LANO scorecard (Le Rhun et al., 2019)

[20] RANO Assessment during clinical routine

[21] Distress-thermometer, EORTC QLQ-C30/EORTC QLQ-BN20/BN20

1. Introduction

Leptomeningeal disease (LMD) is an aggressive subtype of metastatic disease in the central nervous system (CNS) and has a poor prognosis with a median overall survival of a few months. Risk stratification of patients in “good risk” group and a “poor risk” group is currently based on clinical assessments (Karnofsky performance score (KPS), the presence of neurologic deficits, the extent of systemic disease, the existence of treatment options according to the NCCN Guidelines (NCCN, 2021b).

LMD is diagnosed in approximately 5-8% of solid tumors (Clarke, Perez, Jacks, Panageas, & Deangelis, 2010; Le Rhun, Taillibert, & Chamberlain, 2013). LMD leads to multifocal metastases to the leptomeninges, i.e. to the pia mater, arachnoid and subarachnoid space. Malignant cells disseminate via the cerebrospinal fluid (CSF) and can thus lead to leptomeningeal seedings everywhere along the neurospinal axis. Preferential areas are the basilar cistern and the cauda equina (M. C. Chamberlain, 1997). The exact incidence of LMD is difficult to determine because gross examination at autopsy may overlook signs of LMD, and microscopic pathological inspection may be normal if the seeding is multifocal or if an unaffected area of the CNS is examined. Primary solid tumors underlying LMD most often include tumors in skin, lung, breast and gastro-intestinal sytem (Clarke et al., 2010; Hyun et al., 2016; Nayar et al., 2017).

Based on clinical observation, the incidence and prevalence of LMD are both increasing. This might be due to better imaging modalities and improved ability to treat tumors outside the CNS. Thus, LMD has become more frequent as survival has improved for cancer patients. It seems that longer control of non-CNS cancer manifestations of solid tumors allows more time for metastatic spread of tumor cells to the CNS. Another aspect is obviously that the use of large molecule anti-tumor agents might have reduces CSF and CNS penetration. Thus, they might control the systemic disease, however tumor manifestations behind the blood brain barrier, and the blood CSF barrier might not be (yet!) effectively targeted.

There are three growth patterns in LMD: adherent, non-adherent and mixed pattern. These patterns are also referred to as non-nodular, non-bulky versus nodular and bulky. The adherent growth pattern shows nodular contrast-enhancing lesions in the MRI of the brain and/or spinal cord. The non-adherent growth pattern, however, can only be detected by lumbar puncture and subsequent analysis of cerebro spinal fluid.

The clinical features and symptoms of LMD may vary and be subtle. Common clinical symptoms include radicular pain, back pain, cauda equina syndrome, cranial nerve deficits, seizures and gait difficulties. As tumor cell spread in the CSF, this can cause obstacles for CSF flow, thus many patients present with obstructive hydrocephalus, nausea, vomiting and positional headache. In general, new neurological symptoms in tumor patients along multiple areas of the neuraxis are highly suspicious of LMD (M. Chamberlain et al., 2014).

Diagnostic procedures include CSF cytology and magnet resonance imaging (MRI) (details are outlined in the Guideline of the German Society of Neurology (AWMF-Registernummer: 030/060). MRI parameters can include linear ependymal enhancement, cranial and spinal nerve root enhancement, leptomeningeal nodular enhancement. CSF cytology can be positive (defined as the presence of malignat cells in the CSF), equivocal (defined as the detection of atypical or suspicious cells in the CSF) or negative (defined as the absence of malignant or suspicious or atypical cells in the CSF). For standardization of assessments of treatment response, the RANO working group has recently suggested an updated Leptomeningeal Assessment in Neuro-Oncology (LANO) scorecard (Le Rhun et al., 2019).

A general standard of care for LMD does not exist. As LMD patients have been mostly excluded from clinical trial participation all current and relevant information and existing guidelines are mainly derived from case reports or retrospective clinical series. Therapeutic strategies in clinical routine are based on interdisciplinary tumor board decisions and include radiation therapy, probably combined with intrathecal and systemic chemotherapy. The main clinical goals for the treatment of these patients include the improvement of neurological deficits and quality of life. The prolongation of overall survival by treatments is rather in time frames of 2-3 months (Herrlinger et al., 2004). Favorable prognostic factors are younger age at diagnosis, a Karnofsky performance score (KPS) > 70, controlled systemic disease, lack of encephalopathy or focal neurological deficits, low level of protein in the cerebrospinal fluid (CSF) and lack of bulky disease on MRI (Chamberlain M et al., Neuro Oncol 2017;19;484-492).

The risk stratification in a “good risk” or “poor risk” group certainly facilitates the interdisciplinary tumorboard discussions in clinical routine care. The tumorboard discussion needs to carefully consider options for potential systemic chemotherapy, intrathecal therapy or radiation therapy for “good risk” LMD patients. For “poor risk” LMD patients that usually show advanced clinical signs of encephalopathy and serious neurologic deficits, best supportive care strategies or focal (involved-field) radiation therapy are discussed, e.g. to reduce pain.

Regardless of risk group, radiation therapy is a very important corner stone in the treatment of LMD patients. It can be performed at the local site of lesion to improve clinical symptoms, reduce pain or to reduce bulky disease. Patients with multifocal cerebral LMD manifestations usually receive whole brain radiation therapy. Another very important feature of radiation therapy is that it might help to increase drug penetration behind the blood brain barrier or the blood CSF barrier. Depending on interdisciplinary discussions in the tumor board, radiation therapy can be combined with intrathecal therapy (see below) or systemic therapy.

Surgical interventions in clinical routine of LMD patients are usually confined to the placement of intraventricular catheters, e.g. an Ommaya reservoir to facilitate the access to CSF for diagnostic purposes and to ensure uniform drug distribution and delivery during intrathecal therapy (as compared to repeated lumbar administration) (Kesari & Batchelor, 2003; Mack et al., 2016).

1.1. Intrathecal therapy of patients with leptomeningeal disease

Intrathecal (IT) administration of drugs in LMD is an established application route in clinical routine. The intrathecal administration is usually performed by an intraventricular catheter (e.g. Ommay reservoir) and ensures that the drug can act directly in the CSF. In fact, it is the most common method to deliver compounds in non-nodular and non-bulky LMD (i.e. in non-adherent LMD with evidence of malignant cells in the CSF but with lack of LMD signs on MRI). Common drugs that are used for intrathecal administration are methotrexate, cytarabine and thiotepa. Furthermore, intrathecal treatment with Trastuzumab in HER2-positive breast cancer in combination with methotrexate given twice weekly and then weekly has been reported (reviewed (Thomas & Ramirez, 2017). Intrathecal trastuzumab was generally well tolerated without significant side effects.

1.2. Ongoing phase I clinical trial at MD Anderson Cancer Center in metastatic melanoma and LMD

A very recent phase I clinical trial at the MD Anderson Cancer Center (Clinical Trials.gov identifier NCT03025256) currently investigates concurrent intrathecal and intravenous Nivolumab administration in melanoma patients with LMD (Glitza et al., 2020). To date, no further and no final results from this clinical phase I trial have been published. Furthermore, no other clinical trials have been carried out so far using intrathecal PD-1 antibody administration.

Intrathecal Nivolumab was administered via an intraventricular reservoir every 14 days. At the ASCO 2020, the trial group reported on 15 patients with dosages 5 mg, 10 mg and 20 mg. All 15 patients had evidence of LMD on MRI, 8 of 15 patients had positive CSF cytology. Furthermore, 12 of 15 patients had already received prior therapies for their metastatic melanoma; 11 patients had whole brain radiation therapy, 1 patient was treatment-naive. Safety profile was acceptable with only 4 Adverse Events possibly related to intrathecal Nivolumab graded as Grade 2 and 3. The investigators did not detect any Adverse Events > grade 3 that were attributed to IT or intravenous Nivolumab. The median treatment duration was 6 weeks at the time point of presentation. The interim median overall survival analysis at the time point of ASCO presentation was 46.1 weeks (0.1 – 83.3 weeks). The combination of IT and intravenous Nivolumab was well tolerated with no unexpected systemic or neurological toxicity. Taken together, this study group that solely focused on LMD in metastatic melanoma concluded that the IT administration of Nivolumab in addition to intravenous Nivolumab was safe and can thus be administered without additional toxicity in patients with metastatic melanoma and LMD.

1.3. Case report (55-year-old, metastatic melanoma with LMD) from the University Hospital Tübingen

This is a case report of a 55-year-old male who is currently treated at the University Hospital Tübingen with intrathecal Nivolumab administration for metastatic melanoma with LMD. During the course of his disease since 1987, the patient had received interferon-alpha, and after metastases in lymph node, liver and peritoneum received intravenous Nivolumab plus ipililumab in 2017. During the intravenous Nivolumab and ipililumab treatment, he had suffered from autoimmune hypophysitis, pancreatitis, thyroiditis, colitis and uveitis, and had to stop the combined treatment with Nivolumab and ipililumab. His tumor was stable until December 2019, when he developed new metastases and received dabrafenib and trametinib. In July 2020, he experienced a gradual hearing loss and tinnitus. MRI of the brain revealed contrast enhancements suggestive of leptomeningeal disease including the N. vestibulocochlearis on both sides. Further clinical symptoms included vertigo and nausea. A lumbar puncture was performed, and tumor cells were detected in the CSF. The option of cerebral irradiation was discussed with the patient. Since the contrast enhancement was rather focal, the patient did not agree to receive whole brain irradiation. He had information about the ongoing clinical trial at MD Anderson Cancer Center that had used an intrathecal application of Nivolumab, because the trial had just been presented at the ASCO 2020 conference (as trials in progress). We discussed this trial and the experimental status of intrathecal delivery of Nivolumab

together with the patient and his wife and in the tumorboards of the Centers of Neuro-Oncology and Dermatooncology at the Comprehensive Cancer Center Tübingen-Stuttgart, and also asked the Clinical Ethical Board for advice. Both tumorboards and the Clinical Ethical Board opted for an individual medical treatment with intrathecal Nivolumab in the absence of any other options. We informed the patient about the option to insert an Ommaya reservoir or to perform repetitive lumbar punctures. The patient decided to have an Ommaya reservoir. This was implanted on 22 July 2020, and on 24 July 2020, the intrathecal application of Nivolumab started (all visits are outlined in the table below). In September 2020, the MRI of the brain showed an increased volume of the contrast-enhancing areas. Thus, upon discussions in the tumorboard, patient received a single fraction of focal irradiation with 4 Gy on 24 September 2020 while the intrathecal injections continued. The current MRIs and the current CSF assessments do not show any indication for tumor progression.

Table 2 Dosing information for the case report

Date	Intrathecal Nivolumab	Comments
**24/07/2020**	10 mg	n/a
**07/08/2020**	20 mg	Dizziness immediately after injection. Resolved within 10 minutes.
**21/08/2020**	20 mg	n/a
**04/09/2020**	20 mg	n/a
**18/09/2020**	20 mg	n/a<br>Decision to increase dosage to maximum dosage that was applied by Glitza et al., 2020
**02/10/2020**	50 mg	Headache (8/10) after the injection, for 3 days. Completely resolved. Decision to reduce dosage.
**16/10/2020**	40 mg	n/a
**06/11/2020**	40 mg	Hypertension during the next day with 160 mmHg. Completely resolved without medication.
**20/11/2020**	40 mg	n/a
**04/12/2020**	40 mg	n/a

**18/12/2020**	40 mg	n/a
**08/01/2021**	40 mg	n/a
**22/01/2021**	40 mg	n/a
**05/02/2021**	40 mg	Hypertension on the following day with 180 mmHg systolic. Completely resolved.
**19/02/2021**	40 mg	Shivering, 1 minute after injection.
**05/03/2021**	40 mg	n/a
**19/03/2021**	40 mg	n/a
**07/04/2021**	40 mg	Headache, dizziness (mild) for 2-3 days. Completely resolved without medication.
**23/04/2021**	40 mg	n/a
**07/05/2021**	40 mg	n/a
**25/05/2021**	40 mg	n/a

Assessment of Treatment Response in LMD by imaging

The Response Assessment in Neuro-Oncology (RANO) group has suggested criteria for the assessment of parenchymal metastases in the CNS (Lin et al., 2015) and for leptomeningeal diseases (Le Rhun et al., 2019). In this trial, both assessment criteria will be mentioned for all assessments, i.e. RANO and LANO, because RANO is designed for assessing brain parenchymal lesions and LANO is designed to assess LMD.

1.4. Benefit / Risk Assessment

LMD is an area of high unmet clinical need. LMD occurs at a highly advanced stage of cancer disease. As outlined in the NCCN Guidelines (NCCN, 2021b), LMD patients can be defined as “good risk” and “poor risk” based on Karnofsky performance score, neurological assessment and systemic disease. Generally, a decision for any therapeutic strategy for both risk groups is made based on the judgment of the interdisciplinary tumor board. Radiation therapy, intrathecal applications (of methotrexate, AraC or cytarabine) or systemic chemotherapy are discussed in interdisciplinary tumor boards.

Experience with intrathecal therapies in LMD patients so far indicate that this form of therapeutic administration is feasible without additional burden for patients. In fact, this option

is also outlined for LMD patients at "good risk" in the NCCN guidelines. Still, the clinical outcome of LMD patients remains very unsatisfactory with an overall survival in the range of a few months (Glantz et al., 1999; Glantz, Van Horn, Fisher, & Chamberlain, 2010). The IT-PD1 trial group wants to contribute to an improvement of this situation for LMD patients by using an intrathecal application route for the PD-1 antibody, i.e. a drug that has shown clinical efficacy in the underlying tumor via the intravenous route.

Probably the outcome will be improved with an increase of sufficient drug concentrations in the CNS compartment. In fact, major improvement of clinical outcome for solid tumors outside the CNS have been achieved with targeted compounds including checkpoint inhibitors targeting the PD-1/PD-L1 axis. The systemic treatment of several solid tumors with PD-1 or PD-L1 antibodies has indeed improved the clinical outcomes, see list below (SmPc of OPDIVO®, KEYTRUDA® and Tecentriq®).

The IT-PD1 clinical trial centers have a longstanding experience with intrathecal administration of compounds. Furthermore, all participating sites have extensive experience with immunotherapy, checkpoint inhibition and with the clinical management of immune therapy-related Adverse Events.

Consequently, we will use an intrathecal application of PD-1 antibody and thereby circumvent the blood brain barrier. We will focus on LMD in the following metastatic solid tumors where either Nivolumab, Pembrolizuab and/or Atezolizumab are registered for intraveneous application (as outlined in the SmPC):

melanoma, non-small cell lung cancer (NSCLC), Malignant pleural mesothelioma (MPM), renal cell carcinoma (RCC), Classical Hodgkin lymphoma (cHL), squamous cell cancer of the head and neck (SCCHN), urothelial carcinoma, muscle invasive urothelial carcinoma (MIUC), muscle invasive urothelial carcinoma (MIUC) colorectal cancer (CRC) with Mismatch repair deficient (dMMR) or microsatellite instability-high (MSI-H), esophageal squamous cell carcinoma (ESCC), Adjuvant treatment of esophageal cancer (EC) or gastro-oesophageal junction cancer (GEJC), gastro-oesophageal junction (GEJ) or oesophageal adenocarcinoma

Furthermore, triple-negative breast carcinoma with approval for PD-L1 antibody Atezolizumab (Tecentriq®) and Pembrolizumab (KEYTRUDA®) in trastuzumab-resistant breast cancer, e.g. in the PANACEA/Keynote-014 study (Dierks, Pietsch, & Dunst, 2020; Loi et al., 2018).

In addition, leptomeningeal disease of solid tumors with a high tumor mutational burden is also eligible for enrolment into this trial. Based on the phase 2 KEYNOTE-158 trial (NCT02628067), pembrolizumab was approved 2020 by the Food and Drug Administration

(FDA) for patients with refractory solid cancers harboring a TMB ≥10 mut/Mb (Kim et al., 2019).

In general, the above mentioned citerium for patient enrollment is independent of the exact indication for approval (adjuvant, first-line, second-line, etc), independent of the biomarker status and independent of whether a checkpoint inhibitor has been used in the past.

We will use the intrathecal application route for the PD-1 antibody Nivolumab. This application route might increase the bioavailability of this compound in the central nervous system and thus might be better for LMD patients, where the location of tumor cells is “behind” the blood brain barrier. Of note and as outlined above, a recent phase I trial has used an intrathecal application of PD-1 antibody for metastatic melanoma and LMD (Glitza et al., 2020) without complications. This trial group (Glitza et al., 2020) used the PD-1 antibody Nivolumab in their trial. In the present trial, we will extend the study population/entity (i.e. not only metastatic melanoma, but also other solid tumors with a registration for PD-1/PD-L1 inhibition). The Tübingen site has been treating one patient with metastatic melanoma and LMD with Nivolumab since July 2020 (i.e. since 1 year and after ASCO 2020 where the ongoing phase I trial was presented by Glitza and colleagues), all details of this case report are outlined in section 1.3.

The protocol will fully adhere to the toxicity management guidelines for neurological Adverse Events related to therapy with IT Nivolumab as outlined in the NCCN Guideline on Management of Checkpoint Inhibitor Related Toxicities (NCCN, 2021a). The neurological side effects (Myasthenia gravis, Guillain-Barré syndrome, Peripheral neuropathy, Aseptic meningitis, Encephalitis, Transverse myelitis) for participants in this trial are expected to be similar to the well-described neurological intravenous immunotherapy-related Adverse Events (NCCN, 2021a; Pan & Haggiagi, 2019). The most frequent adverse reactions (≥ 10%) in connection with intravenous Nivolumab as monotherapy across tumour types as outlined in the SmPC were fatigue (44%), musculoskeletal pain (28%), diarrhoea (26%), rash (24%), cough (22%), nausea (22%), pruritus (19%), decreased appetite (17%), arthralgia (17%), constipation (16%), dyspnoea (16%), abdominal pain (15%), upper respiratory tract infection (15%), pyrexia (13%), headache (13%), anaemia (13%) and vomiting (12%). The majority of these adverse reactions were mild to moderate (Grade 1 or 2). The incidence of Grade 3-5 adverse reactions was 44%, with 0.3% fatal adverse reactions attributed to study drug.

All participating sites have longstanding clinical experience with immunotherapy (e.g. PD-1 antibody, vaccination clinical trials), therefore all participating sites are familiar with the NCCN Guidelines.

Trial participants will be examined carefully to ensure that all treatment-related side effects will be detected immediately and treated effectively. Of note, patients suffering from LMD do not have any curative therapeutic options.

Intrathecal applications are routinely either performed via lumbar punctures or via intraventricular reservoir. Repetitive CSF drawing might potentially bear the risk of developing a hygroma, a condition that is caused by a decrease of intracranial pressure. Yet, in the IT-PD-1 trial, the intrathecal application procedure will be performed via an intraventricular reservoir, whereby the amount of withdrawn CSF equals the amount of applied volume. Furthermore, drawing as well as intrathecal applications will be performed over 5 minutes respectively. Therefore, we consider the risk of a decrease in intracranial pressure and the development of a hygroma as negligible. Of note, current guidelines (NCCN and ESMO) recommend the application of intrathecal therapy in clinical routine by an intraventricular route (instead of repetitive lumbar punctures), e.g. use of an intraventricular reservoir also in order to ensure consistent dosing and results. Intrathecal application via a reservoir is thus a well-established procedure. As for any intervention, there is a principle probability of risks including infections. Yet, with a guideline-based standard operational procedure including a strictly sterile handling and work, these risks can be minimized and almost excluded.

A lumbar puncture will be only necessary prior to the enrollment in this trial as part of the clinical routine diagnostics for patients with suspected LMD. Thus, the lumbar puncture is not a study-specific procedure for this trial but a procedure that would occur anyway in the clinical routine diagnostics of patients. Lumbar puncture is a guideline-based intervention, part of clinical routine and performed frequently and daily in all IT-PD1 clinical trial centers. The DGN guidelines for LP will be fully complied. As for any intervention, there is a principle probability of risks. Yet, in the case of LP, a thorough handling by an experienced center of neurology according to the DGN guidelines during the procedure reduces all these risks (including bleeding, inflammation, meningitis, pain, tissue damage, transient or permanent paralysis) and make them highly unlikely. Some patients may suffer post-puncture headaches. This event cannot be predicted or foreseen beforehand. Yet, it is a treatable condition according to DGN guidelines. The benefits of an LP (and the reason why this is a guideline-based diagnostic tool in the context of LMD) are as followed: information on differential diagnosis of LMD (i.e. particularly meningitis, aseptic meningitis), the detection of non-adherent tumor spread in the CSF (that cannot be captured by MRI). These information’s are necessary prior to clinical trial enrollment.

Repetitive CSF assessments during the treatment phase of the clinical trial are important, and will be performed prior to the intrathecal Nivolumab administration via reservoir (see above). Of note, the CSF is the most accurate fluid to perform a recording of treatment-related immunogenicity in the central nervous system. Of particular importance is the detection of any treatable (!) immunotherapy-related Adverse Events (e.g. aseptic meningitis) that is only possible by CSF assessments.

Taken together, there is a clear positive risk/benefit assessment for CSF collection: very low risk and burden for the patient, clear benefit in terms of safety and significance for the individual study participant and also for the evaluation of the study results.

A recent study suggests that PD-1 blockade interferes with nociception in rodents and nonhuman primates (Wang et al., 2020). The reason might be that the PD-1/PD-L1 axis modulates the nociceptive neuron activity (Chen et al., 2017). It is currently unclear if this holds also true for the human situation and for cancer patients. Yet, to reduce the potential risk of altered nociception for study participants in the context of intrathecal delivery, we have included a very carefully monitoring for the detection of allodynia in the clinical assessments of until Follow-up. The occurrence of of allodynia or increased pain sensation will be monitored clinically (as integral part of the medical history) and by the following neurological examinations: Necessary is the assessment of sympathetic skin response (per standard of care) before the start of intrathecal Nivolumab and after 6 applications. Optional and only if applicable in the participating clinical trial centers, a quantitative sensory testing (QST) can be performed for those sites with available QST equipment.

Taken together, (1) this trial will treat here a cancer stage with very high unmet clinical need without efficient treatment options and a very poor prognosis, (2) we have selected highly qualitfied clinical sites, (3) we will use a well-established application technique and route (intathecal applications are often applied and well established either via lumbar puncture or via reservoirs), (4) we will use the PD-1 antibody Nivolumab that has been administered intrathecally in metastatic melanoma and LMD (Glitza et al., 2020) and in our case report. No further clinical data on intrathecal Nivolumab application are accessible and available at this time. (5) We will use the intrathecal Nivolumab application in LMD of those metastatic solid tumors that are treated with Nivolumab, Pembrolizumab or Atezolizumab in clincal routine. In addition, leptomeningeal disease of solid tumors with a high tumor mutational burden is also eligible (see list above). (6) We will carefully assess altered pain sensations to ensure the

detection of any signs of allodynia. We conclude that a benefit/risk assessment based on these facts favors the benefit for each enrolled patient.

The investigated patient group is an at-risk cohort for severe infections. However, these patients require a thearpy option and a regular follow up. The risk of severe infections is hence not increased through participation in this trial. The participating sites strictly follows recommendations for prevention of acute pandemic situations.
Throughout conduct of the study, continuous documented monitoring and reevaluation of benefit / risk assessment for the trial and individual recruited patients will be performed by the sponsor and investigator, respectively.

1.5. Advisory Committes

1.5.1. Data and Safety Monitoring Board (DSMB)

An independent Data and Safety Monitoring Board (DSMB) will be assembled. The DSMB will be composed of independent experts in the field of (neuro-)oncology assessing the progress, safety data and critical efficacy endpoints. The mission of the DSMB will be to ensure the ethical conduct of the trial and to protect the safety interests of patients in this trial.

The DSMB will be informed at each dose level (cohorts 1 with 20 mg dosage, cohort 2 with 30 mg dosage, cohort 3 with 40 mg dosage, cohort 4 with 50 mg dosage) in Part I of the study. The DSMB will receive a report in writing listing and summarizing all safety data, including dosing. Additionally, the report will provide data concerning recruiting rates and status of the trial. If the DSMB has no urgent concerns, the study will proceed to the next higher cohort.

Before proceeding to Part II there will be a safety interim review by the DSMB. Based on its review, the DSMB makes a recommendation to the sponsor to determine the tolerated dosage to proceed with Part II of the study.
An emergency meeting of the DSMB may be called at any time, should questions of patient safety arise and necessary safety reports will be provided. Meetings may be convened as conference or calls as well as in person.
The activities of the DSMB are described in the DSMB Charta.

2. Study Objectives

2.1. Primary Objective and Endpoint

This trial will investigate the **maximum tolerable dose** and **safety** of intrathecal PD-1 antibody administration in LMD of metastatic solid tumors with a registered indication for treatment with intravenous PD-1 antibody or PD-L1 antibody. The appropriate dose for the expansion phase (Part II) is based on the results in Part I (dose escalation cohort study, see section 3) and will define the maximum tolerable fix dose in Part II.

Dose Limiting Toxicities are defined as following:

- CTCAE grade 4 or above Adverse Events related to the IMP.
- Neurological CTCAE grade 2 and 3 Adverse Events related to the IMP that have a recommendation of permanently discontinuation of immunotherapies according to the “NCCN Guideline on Management of Checkpoint Inhibitor related Toxicities” (NCCN, 2021a), see also Appendix III (section 14.3).

The **AE assessment** includes clinical and imaging signs (per LANO, see Appendices I). Please note that imaging will be every 12 weeks per standard of care. Yet, if any of the above-mentioned clinical features occur, MR imaging will be performed immediately to investigate the following conditions:

- Increased subarachnoid or ventricular nodules in the brain/spine
- Worsening of leptomeningeal linear enhancement in the brain/spine
- Worsening of hydrocephalus in the brain
- Progression of metastases in the CNS parenchyma (if patient had metastases in the CNS parenchyma at trial entry)

The assessment of the primary objectives will be accomplished by **clinical assessements, documentation of Adverse Events and grading** according to the Common Terminology Criteria for Adverse Events (CTCAE V5.0) from time of signing the Informed Consent until the end of the last Follow-up 4 after last dose within the scope of the clinical study. The safety endpoints will be assessed by a review of Adverse Events and serious Adverse Events.

2.2. Secondary Objectives and Endpoints

The secondary endpoint is **overall survival** defined as the time interval from the date of first study administration to the date of progression.

2.3. Exploratory Objectives

The exploratory objectives will allow translational assessments that shall help to design subsequent trials upon the commencement of this phase I trial.
These include

- To determine the reommended dosage of IT administration of PD-1 antibody
- Patient-reported outcome as assessed by EORTC QLQ-C30/EORTC QLQ-BN20, Distress thermometer (Mehnert, Müller, Lehmann, & Koch, 2006)
- Neurocognitive assessments as assessed by Mini Mental Status (MMSE) Score; Montreal Cognitive Assessment (MoCa) Score during intrathecal applications
- Longitudinal changes of cfDNA, immunopeptidome, cytokines during IT PD-1 treatment in CSF and peripheral blood
- Association of PD-1 and PD-L1 expression status of the primary tumor and LMD with progression-free and overall survival

3. Study Design

This is a prospective, interventional, open label, multicenter phase I trail in leptomeningeal disease in patients with solid tumors that have a registered indication for intravenous treatment with PD-1 antibody. The study consists of two parts:

To reflect the primary objective, the study is designed as a dose - escalation trial with a "3 + 3 design" (figure 1). There will be 4 cohorts, each with a fixed predefined dosage (20 mg, 30 mg, 40 mg, 50 mg). In Part I (figure 2) minimal 12 (maximally 24) subjects will receive an intrathecal Nivolumab treatment.

For the "3 + 3 design" three subjects are initially enrolled into cohort 1 with a given dose of 20 mg. If Safety Visit 1 is completed and there is no DLT (dose limiting toxicity) observed in any of these subjects, the trial proceeds to enroll three subjects into the next higher dose cohort 2 with a given dose of 30 mg. If there is no DLT observed in any of these subjects, the trial proceeds to enroll additional three subjects into the next higher dose cohort 3 with a given dose of 40 mg. If there is no DLT observed in any of these subjects, the trial proceeds to enroll additional three subjects into the next higher and last dose cohort 4 with a given dose of 50 mg.

Drop-outs before Safety Visit 1 due to tumor progression, death, withdrawal of IC or other reasons, but without Dose Limiting Toxicity, will be replaced to reach 3 subjects in each cohort for the safety assessment. Of course, the subject will still be reported within the intention-to treat population (see also chapter "drop out").

If one subject develops a DLT at a specific dose, three additional subjects are enrolled into that same dose cohort. Development of DLTs in more than 1 of 6 subjects in a specific dose cohort suggests that the MTD (maximum tolerated dose) has been exceeded, and further dose escalation is not pursued. In this case and if the DSMB does not have any reasonable, secured and causal concerns an extension of the previous maximum tolerated dose is proposed. Cohort 1 is an exception: if limiting toxicity at this lowest dose level has been identified in more than 1 of 6 subjects, the study will be terminated. The MTD is based on number of patients experiencing DLT and not on number of events amongst a defined number of patients.

The exposure of subjects to intrathecal Nivolumab follow a staggered approach (see section 6.5 Dose schedule and figure 4).

The DSMB will be conducted after the first three (or six) patients of each cohort have completed Safety Visit 1. For each cohort there will be an interim safety analysis, all Adverse Events will be assessed by the data safety monitoring board (DSMB) as outlined in the DSMB Charta. At

the end of Part I of this clinical trial the maximal tolerated dosage shall be determined. This will be the fix dose for Part II.

In Part II (figure 3) minimal 20 (maximally 25) subjects will receive an intrathecal PD-1 treatment with a fixed dose, depending on the results from Part I.

In this clinical trial, each subject in Part I and II will receive a total of 6 intrathecal applications of Nivolumab every 2 weeks. After every 3 doses, an additional Safety Visit (1 and 2) takes place. After the last dose, the subject enters the Follow-up phase. Subjects who tolerate the intrathecal application after 6 doses and do not show any signs of progression can continue the treatment outside the study after consultation with the sponsor and their investigator.

All patients enrolled into the trial and receiving at least one intrathecal application of PD-1 will belong to the Intention-to-treat (ITT) population (see also section “drop out”).

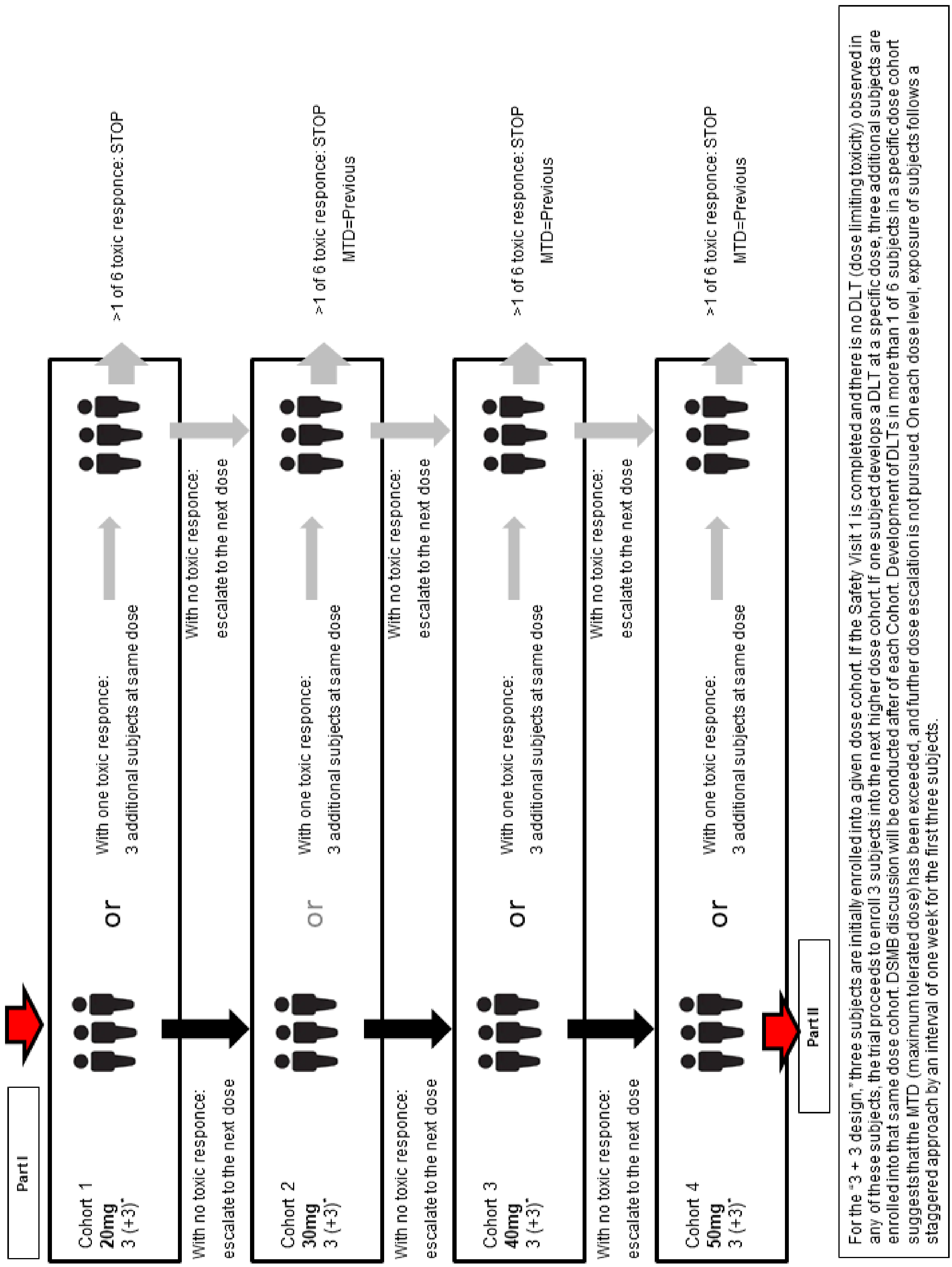

Figure 1 3x3 design with 4 cohorts

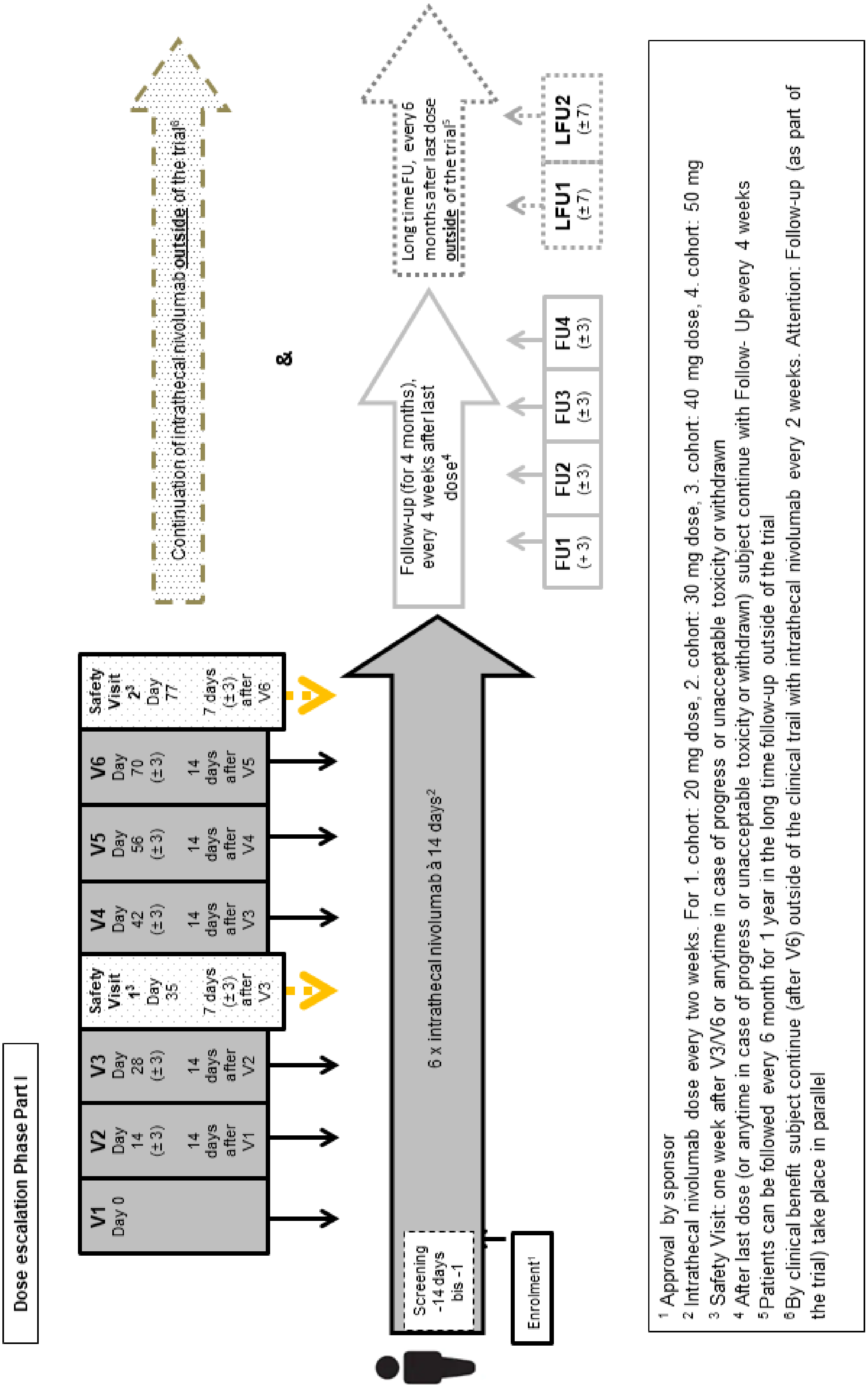

Figure 2 Overall Study Design for Part I

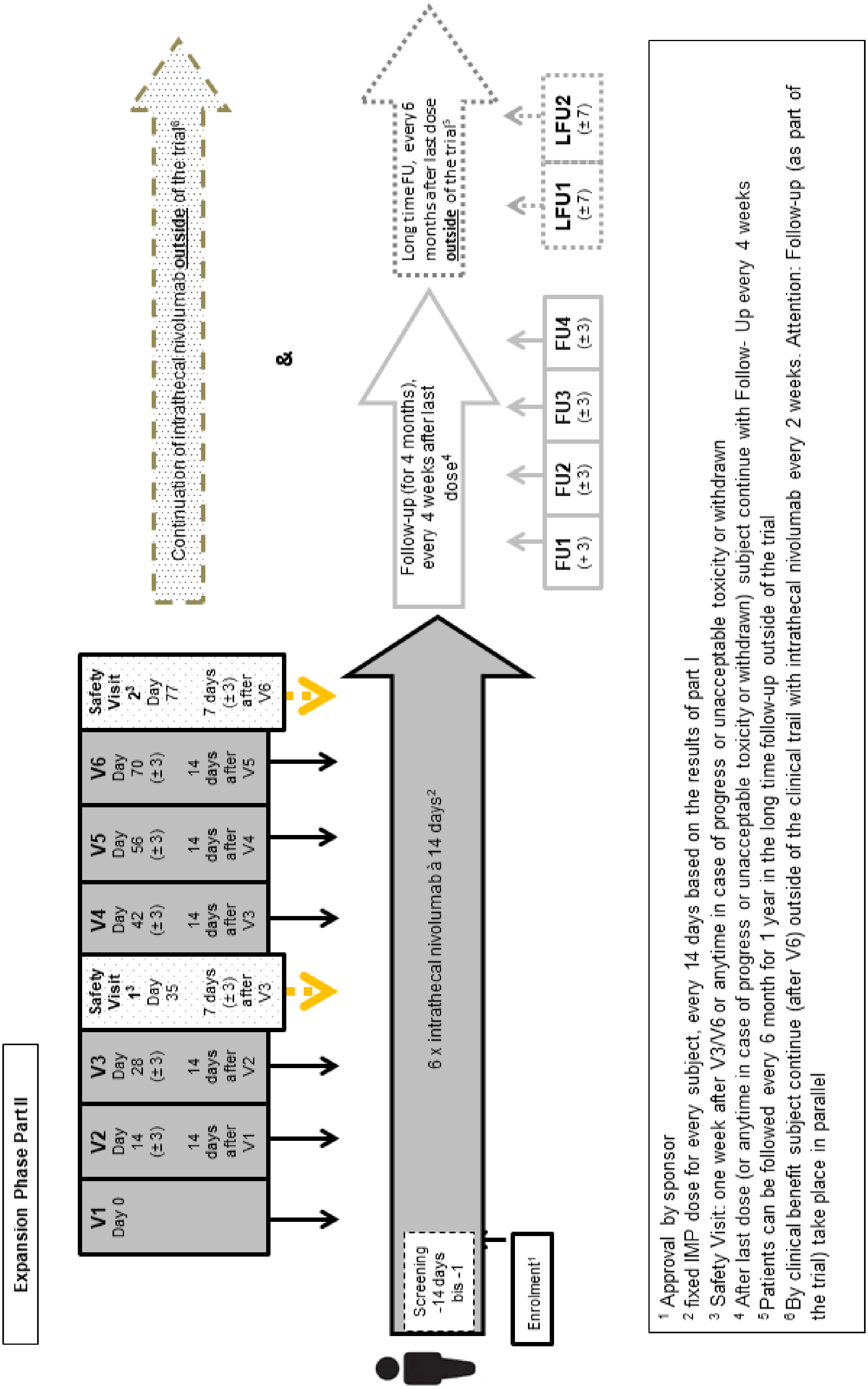

Figure 3 Overall Study Design for Part II

3.1. Study Duration and Schedule

The duration of the trial for each subject is expected to be approximately 28 weeks. The duration for each individual subject includes up to 2 weeks screening, 10 weeks study treatment plus a safety as well as 16 weeks Follow-up time. Patients who tolerate intrathecal Nivolumab and do not show signs of progression, can continue intrathecal Nivolumab outside the trial.

The overall duration of the trial is expected to be approximately 3 years including the preparatory phase. Recruitment of subjects will start in Q4 2021. The actual overall duration or recruitment may vary. The currently estimated study timelines are described in Table 3.

Table 3: Study Timelines

Total trial duration	72 months
Clinical trial duration	60 months
Duration for in individual patient	Screening: up to 14 days<br>Study treatment: 10 weeks and additional Safety Visits<br>Follow-up: approx. 4 months
FSI (First Subject In)	Q4 2021
LSI (Last Subject In)	QI 2026
LSO (Last Subject Out)	Q3 2026
DBL (Data Base Lock)	Q4 2026
Statistical Analyses Completed	Q1 2027
Trial Report Completed	Q3 2027

3.2. End of Study

<u>End of Study of Subjects</u>

The End of Study for a patient enrolled in this trial is the last Follow-up Visit 4 (after last dose or 6th dose, whatever occurs first). The IMP can be permanently suspended in case of progressive disease or unacceptable toxicity or withdrawal of consent.

<u>End of complete Study:</u>

The end of study is defined as follows: Last Patient Last Visit.

4. Study Population

This study will screen and enroll patients with metastatic solid tumors and LMD. The underlying primary tumor must have a registered indication for intravenous application of PD-1 or PD-L1 antibody. The present trial will use an intrathecal delivery of Nivolumab in these patients.

4.1. General Criteria for Subject Selection

Adult male and female patients fulfilling the below outlined inclusion criteria will be enrolled into the study. Trial population will consist of both genders. Gender distribution in the trial is supposed to reflect the distribution in the real patient's population (approx. 60% male and 40% female patients), there will be no prior defined quantitative ratio between females and males. No statistical analysis will be performed based on the genders or differences between the genders.

4.1.1. Inclusion Criteria

Subjects meeting all of the following criteria will be considered for admission to the trial:

1. Must be ≥ 18 years at the time of signing the informed consent.
2. Understand and voluntarily sign an informed consent document prior to any study related assessments/procedures.
3. Patients with a "good risk" status as defined by the NCCN guidelines (version 1.2021)
4. Tumor board protocol confirming:
- a clinical recommendation for intrathecal therapy and evaluation of trial enrollment
- a statement on the potential necessity of additional systemic treatment of metastatic tumor outside the CNS
5. Able to adhere to the study visit schedule and other protocol requirements.
6. All subjects must agree to refrain from donating blood while on study drug and for 28 days after discontinuation from this study treatment.
7. Patients with Karnofsky performance score > 50%
8. Diagnosis of LMD by CSF and/or MRI
    a. A thorough CSF evaluation must be performed in every patient prior to the inclusion in this trial. The reason is that a positive CSF cytology is considered

the gold standard for LMD diagnosis. Furthermore, a thorough CSF evaluation will allow the thorough assessment of potential differential diagnoses (for example viral meningitis, bacterial meningitis, aseptic meningitis, sarcoidosis etc.)

b. Presence of malignant cells on CSF cytology. The frequency of CSF evaluation is based on guideline of the German Society of Neurology: Please note that the first lumbar puncture is only 50-60% sensitive. Repeat collection increases sensitivity up to approximately 80%. Thus, a negative first CSF evaluation should at least be repeated once. According to guidelines from the German Society for Neurology each CSF collection should draw enough, i.e. at least 5-10 ml CSF and should be processed within one hour of collection.
c. MRI diagnosis of LMD: pial enhancement, pial nodular manifestations (as defined per LANO criteria, see appendix).
d. A positive CSF cytology and an MRI evidence is enough to determine the LMD diagnosis.
e. Please note that approximately 20% of patients with symptomatic LMD might lack positive CSF cytology even upon repeated puncture. In these cases, the LMD diagnosis can also be performed based on cerebral/spinal MRI manifestations and by exclusion of differential diagnosis.
f. In the absence of diagnostic findings for LMD in the CSF: patients must present with typical clinical and MRI signs of LMD (Le Rhun et al., 2017). If the CSF has signs of pleocytosis (BUT NOT any malignant, atypical or suspicious cells) the differential diagnosis for CSF pleocytosis (aseptic meningitis, viral meningitis, bacterial meningitis) must be excluded.
g. Some centers perform biopsies of leptomeninges for obtaining a LMD diagnosis. The LMD diagnosis will be based on histology and should be documented accordingly. Yet, a histological diagnosis of LMD is NOT required for the inclusion in this trial.

9. If radiation therapy had occurred: Please make sure that a documentation of the past radiation therapy is available (including applied dosage and radiation therapy fields):
   a. Participants eligible for IT-PD1 should have completed their radiation therapy due to clinical indication > 2 weeks prior to enrollment into the trial.
   b. All LMD patients without an indication for radiation therapy (per investigator`s choice) can be enrolled immediately
10. Neurological examination (NANO scale) (Nayak et al., 2017).

11. MRI: the assessment at baseline and for subsequent time points should be based on the LANO scorecard (see appendix) according to (Le Rhun et al., 2019).
12. Ability to undergo intrathecal therapy via an intraventricular catheter (e.g. Ommaya reservoir).
13. Primary tumor tissue for the assessment of PD-1 and PD-L1 is optional at the timepoint of inclusion and enrollment but does need to be shipped before end of the trial.
14. Female Patient of childbearing potential[1] and male patients with female partner of childbearing potential[1] is willing to use highly effective contraceptive methods during treatment and for 150 days (male or female, see SmPC) after the last dose. Recommendations highly effective contraceptive methods are:
    a. combined hormonal contraception associated with inhibition of ovulation (oral-, intravaginal, -transdermal)
    b. progestogen-only hormonal contraception associated with inhibition of ovulation (pral injectable, implantable),
    c. intrauterine device (IUD),
    d. intrauterine hormone - releasing system (IUS),
    e. bilateral tubal occlusion,
    f. vasectomized partner[2],
    g. sexual abstinence[3]

[1] For the purpose of this document, a female is considered of childbearing potential (FCBP), i.e. fertile, following menarche and until becoming post-menopausal unless permanently sterile. Permanent sterilisation methods include hysterectomy, bilateral salpingectomy and bilateral oophorectomy. A postmenopausal state is defined as no menses for 12 months without an alternative medical cause. A high follicle stimulating hormone (FSH) level in the postmenopausal range may be used to confirm a post-menopausal state in women not using hormonal contraception or hormonal replacement therapy. However, in the absence of 12 months of amenorrhea, a single FSH measurement is insufficient. For the purpose of this document, a man is considered fertile after puberty unless permanently sterile by bilateral orchidectomy.

[2]Vasectomized partner is a highly effective birth control method provided that partner is the sole sexual partner of the WOCBP trial participant and that the vasectomized partner has received medical assessment of the surgical success

[2] In the context of this guidance sexual abstinence is considered a highly effective method only if defined as refraining from heterosexual intercourse during the entire period of risk associated with the study treatments. The reliability of sexual abstinence needs to be evaluated in relation to the duration of the clinical trial and the preferred and usual lifestyle of the subject.

4.1.2. Exclusion Criteria

Subjects presenting with any of the following criteria will not be included in the trial:

1. Women during pregnancy and lactation.
2. Previous intrathecal Nivolumab application.
3. Patient at “poor risk” (NCCN guidelines version 1.2021).
4. The following differential diagnoses to LMD are exclusion criteria:
    a. Aseptic meningitis
    b. Viral meningitis
    c. Bacterial meningitis
5. History of hypersensitivity to monoclonal antibodies.
6. Participation in other clinical AMG or MDR trials or observation period of competing trials or if there is otherwise a high risk of insurance law issues intervening between two studies and if the participation affects the primary endpoint of the IT-PD1 study. In case of uncertainty, competing insurances must be contacted prior to participation
7. A clinical condition that in the opinion of the investigator would interfere with the evaluation or interpretation of patient safety or trial results or that would prohibit the understanding of informed consent and compliance with the requirements of the protocol.
8. Any treatment-related toxicities from prior systemic anti-tumor or immune therapy not having resolved to CTCAE version 5.0 grade 1, with the exception of alopecia.
9. Patient with confirmed hisory of current autoimmune disease.
10. Patients with any disease resulting in permanent immunosuppression or requiring permanent immunosuppressive therapy.
11. Clinically significant active infection, for example:
    a. Presence of human immunodeficiency virus
    b. Active hepatitis B virus/hepatitis C virus. HIV infection or active Hepatitis B or C infectionor active infections requiring oral or intravenous antibiotics or that can cause a severe disease and pose a severe danger to lab personnel working on patients’ blood or tissue (e.g. rabies) *.
12. Inability to undergo MRI with contrast agent.
13. The underlying primary tumor has not a registered and authorized indication in the European Union for intravenous treatment with Nivolumab, Pembrolizumab or

Atezolizumab. The solide tumor registered are, i.e. melanoma, non-small cell lung cancer (NSCLC), Malignant pleural mesothelioma (MPM),renal cell carcinoma (RCC), Classical Hodgkin lymphoma (cHL), squamous cell cancer of the head and neck (SCCHN), urrothelial carcinoma, muscle invasive urothelial carcinoma (MIUC), colorectal cancer (CRC) with Mismatch repair deficient (dMMR) or microsatellite instability-high (MSI-H), esophageal squamous cell carcinoma (ESCC), Adjuvant treatment of esophageal cancer (EC) or gastro-oesophageal junction cancer (GEJC), Gastric gastro-oesophageal junction (GEJ) or oesophageal adenocarcinoma, triple-negative breast carcinoma. In addition, leptomeningeal disease of solid tumors with a high tumor mutational burden is also eligible.

14. Abnormal laboratory values for the following values in haematology, coagulation parameters, liver and renal function:
    a. Haemoglobin < 8 g/dl
    b. White blood cell count < 2.0 x 10^9/L)
    c. Platelet count decrease < 50 x 10^9/L
    d. Bilirubin > 2.5 x upper limit of normal (ULN) according to the performing laboratory`s reference range. Note that benign hereditary hyperbilirubinemia e.g. Gilbert`s syndrome is permitted.
    e. Alanine aminotransferase > 3 x ULN
    f. Aspartate aminotransferase > 3 x ULN
    g. Serum creatinine increase > 1.5 x ULN
15. Patients who have received live or attenuated vaccine therapy used for prevention of infectious disease within 4 weeks of the first IT application of Nivolumab.
16. Patients requiring chronic systemic corticosteroid therapy (> 10 mg prednisone or equivalent per day) or any other immunosuppressive therapies (including anti-TNF-a therapies).

*) These parameters are necessary in immunotherapy studies, as they in turn may have an impact on immune parameters (independent of study treatment)

5. Requirements for Trial Site and Investigator

The trial site must have documented experience with immunotherapy trials in Neurology/Neurooncology and oncology and a local pharmacy with experience in PD-1 antibody handling. The local investigators have experience with intrathecal administration of compounds. Furthermore, all participating sites have experience with the clinical management of immune therapy-related Adverse Events.

6. General Information on the Investigational Medical Product

Nivolumab (OPDIVO®) is a marketed pharmaceuticals material authorized in the European Union and will be purchased from the respective local pharmacy of trial sites. In this trial, we will use an off-label delivery route of Nivolumab, i.e. we will use an intrathecal application as was performed in a recent phase I trial at MD Anderson Cancer Center (Glitza et al., 2020). The MDACC trial (ClincialTrials.gov-identifier NCT03025256) is also an Investigator Initiated study. The study already uses Nivolumab intrathecally. From this we conclude, that from a regulatory point of view (at least in the USA) it can be administered intrathecally. After consultation with BMS, the manufacturer of Nivolumab does not have any data (including toxicological data) from the intrathecal study. Of note, this recent phase I trial was confined to melanoma patients with LMD. Our trial will cover a broader study population (see in section 4.1.1). Of note, we have data from an individual medical treatment of a patient with metastatic melanoma and meningeosis melanomatosa, which we present as a case report in this protocol (see section 1.3).

6.1. Manufacturing of the Investigational Medicinal Product

We will use the commercially available PD-1 antibody Nivolumab. All characteristics and details are outlined in the SmPC.

Nivolumab - Opdivo has a concentration of 10 mg/ml. The concentration always remains the same, as the undiluted solution is always used. In the present study, intrathecal administration is planned, i.e. application into the cerebrospinal fluid (intrathecal applications). For intrathecal use, the undiluted drug will be prepared. Undiluted use is permitted according to the Fachinformation for Nivolumab (Opdivo®). Further information on stability, follow the SmPC. Of note, it is recommended to prepare the intrathecal preparations on and for the day of administration. When preparing the dose for participants of the IT-PD1 trial, the local preparation standards of the pharmacies involved must be considered. No filters or similar are required for the application. In addition to the Nivolumab administration, a 5 ml NaCl 0.9% for rinsing must be provided.

6.2. Labelling of the Investigational Medicinal Product

Nivolumab (OPDIVO®) will be ordered from the local pharmacies (20 mg, 30 mg, 40 mg or 50 mg). The study site have to maintain a drug accountability log. A complete record of batch numbers and expiry dates of all study treatments will be maintained in the ISF. The number of administered IMPs will also be recorded in the eCRF. Special labeling for the present study is not necessary as treatment is unblinded. However, to avoid unauthorized usage, the package will be marked as containing study medication, protocol number and Patient ID by the local pharmacies.

6.3. Storage of the Investigational Medicinal Product

The medication must be kept in a locked area with access restricted to designated staff. The medication shall be stored according to well-established handling and storage instructions in the Fachinformation for Nivolumab (Opdivo®)

- Store in the refrigerator (2°C - 8°C).
- Do not freeze.
- Store in the original packaging to protect the contents from light.
- The unopened vial can be stored for up to 48 hours at a controlled room temperature of up to 25°C and under room lighting.

Further storage conditions after preparation and information on the shelf life are given in the Fachinformation of Nivolumab and are, hence, not further specified in this protocol. Please note that this drug is a widely-used drug in the treatment of cancer patients. Thus, the local pharmacies are very well equipped and well trained with its usage.

6.4. Drug Accountability, Therapy Compliance and Disposal

Trial medication will be dispensed to the subject by the investigator and must be documented on the drug accountability form. A member of the study team will document the date of dispensary, subject identification, batch/ serial numbers or other identification of nivolumab and NaCl solution andwill also keep accurate records of the quantities of nivolumab (20mg, 30mg, 40mg or 50mg) dispensed or that was discarded by each subject. Details will be recorded in the Case Report Form (CRF). The study drug (20mg, 30mg, 40mg or 50mg) will be supplied only to subjects participating in the study and in accordance with this protocol. The study drug (20mg, 30mg, 40mg or 50mg) may not be relabeled or reassigned for use by other

trial subjects. Since commercial goods are used, unused or expired medication is handled according to local pharmacy standards for further processing/destruction.

6.5. Dose Schedule

This is a single arm phase 1 trial all patients of Part I and II as outlined above (section 3).

For Part I there will be four cohorts:

Cohort 1 with a fix dose of 20 mg,

Cohort 2 with a fix dose of 30 mg,

Cohort 3 with a fix dose of 40 mg

Cohort 4 with a fix dose of 50 mg

On each dose level, exposure of subjects to intrathecal Nivolumab will follow a staggered approach by an interval of one week minimum for at least the first three subjects for the first three IT application of each cohort (see figure 4). That is to say, at each given dose level the second subject is only exposed once 7 days have passed from the first subject exposed to that dose level, and the third subject is only exposed once 7 days have passed from the second subject's first Nivolumab dose.

If no limiting toxicity has been identified and if the DSMB does not have any concerns, the study will continue with the next cohort. If there is a limiting toxicity identified in one patient, three additional patients will be included and treated in the same cohort. If no limiting toxicity has been identified in the three additional patients and if the DSMB does not have any concerns, the study will continue with the next cohort. If limiting toxicity has been identified in more than 1 one patients of cohort 1, the study will be terminated. In case of toxicities in more than one patient at all higher dose levels (cohort 2, 3 and 4) an extension of the previous maximum tolerated dose is proposed.

The starting dose of 20 mg was chosen because of the following reasons:

(i) the clinical trial NCT03025256 in metastatic melanoma patients with LMD (Glitza et al., 2020) presented dose escalation up to 20 mg at ASCO 2020 without significant CNS toxicity (and aimed at increasing up to 50 mg in their outlook);

(ii) in the case report presented above (see section 1.3) the dosage up to 40 mg is very well tolerated by the patient.

(iii) this trial wants to prevent that LMD patients (that per definition have an extremely poor prognosis and high unmet clinical need) are treated with intrathecal concentrations that are too low (i.e. 5 mg and 10 mg)

The maximum dosage of 50 mg was also chosen:

(i) based on the presentation of clinical trial design of NCT03025256 (Glitza et al., 2020) that aimed at increasing the intrathecal dosage up to 50 mg.

(ii) based on the case report. We increased the dosage up to 50 mg. The patient did not tolerate this dosage (severe headache), and the dose needed to be reduced. He has continued since then on 40 mg intrathecal Nivolumab.

The stepwise dose escalation by 10 mg steps for this trial was defined based on the experiences from the case report (dose escalations 10 mg, 20 mg, 50 mg and then 40 mg) and based on the escalation steps in the clinical trial by Glitza and colleagues (Glitza et al., 2020), who chose 5 mg, 10 mg, 20 mg and then increased up to 50 mg. We reasoned that 10 mg steps will be most appropriate.

The intrathecal administration of Nivolumab will be performed over 5 minutes. When the study drug is administered for the first time, the patient should remain 6 hours at the center for Follow-up unless the local investigator decides that from a clinical point of view an inpatient admission is necessary.

For all subsequent intrathecal administrations (V2-V6), only 1 hour of Follow-up is required.

The intrathecal Nivolumab application shall occur via an intraventricular catheter. Before each intrathecal application, the collection of 10 ml CSF for diagnostic requirements are necessary.

The intraventricular reservoirs (Ommaya or Rickham) are connected to a brain ventricle via a catheter. They allow repeated CSF sampling from for diagnostic investigations or for intracranial pressure reduction and is used for intrathecal application. For example, the subgaleal located Ommaya reservoir describes a catheter system specially developed for local chemotherapy. The outgoing catheter leads into a cerebral ventricle. A thin cannula allows the reservoir to be inserted and makes it possible on the one hand to drain off cerebrospinal fluid for medical diagnostics (cerebrospinal fluid diagnostics), and on the other hand this system allows an intrathecal application of a compound. The Ommaya reservoir has the advantage that the locally very limited injection of the IT antibody is gentler on the organism as a whole. In addition, it allows the direct injection of the active substance into the cerebrospinal fluid bypasses the blood-brain barrier, which is often a therapeutic obstacle.

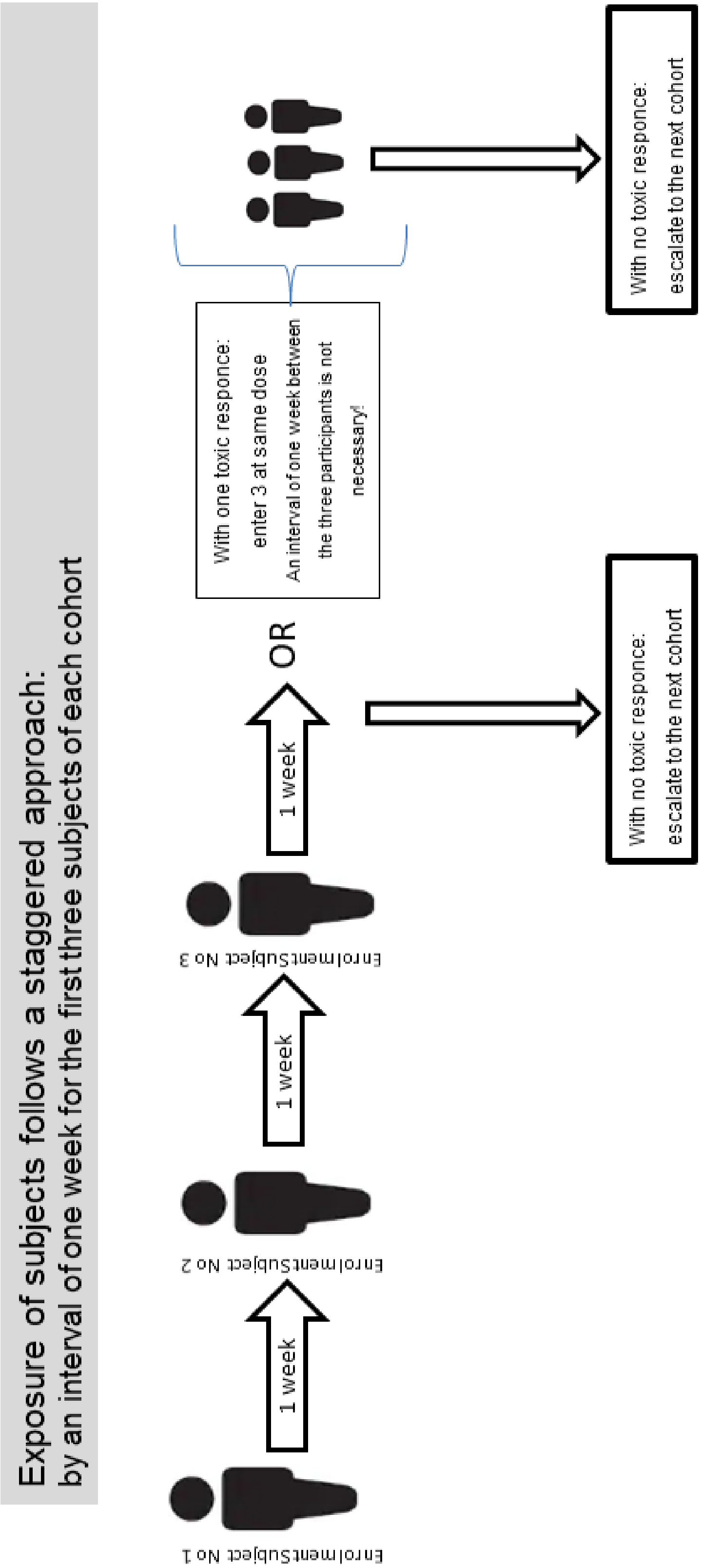

Figure 4 staggered exposure of subjects for each cohort

6.6. Dose Modification and DLT (Dose Limiting Toxicities)

Please note that the intrathecal Nivolumab application is restricted to the ventricular route. Thus, a tumor board recommendation for the intrathecal therapy must be documented prior to enrollment into the trial (see inclusion criteria 4).

Please note: 20 mg is the minimal administered dose in this trial. 50 mg is the maximal administered dose in this trail. No intra-patient dose escalation or reduction is allowed. The results of Part I (depending on the DSMB decision) will be the maximum tolerable fix dose for Part II.

All ongoing treatments for tumor manifestations outside the CNS should be documented in the section “concomitant medication”. Prior therapies and surgeries will be documented separately.

If patients receive additional systemic PD-1 or PD-L1 inhibition (per tumorboard recommendation as outlined in inclusion criteria 4), the intrathecal application can be performed. Yet, intravenous and intrathecal applications must not be performed on the same day. According to the trial protocol of the MD Anderson Cancer Center Study Group (Glitza et al., ASCO 2020), the latency between intrathecal and intravenous application should be one day.

If systemic PD-1/PD-L1 inhibition is applied in parallel to intrathecal Nivolumab application, both treatments need to be stopped if immune-related toxicities occur that necessitate discontinuation.

If patients had received systemic PD-1/PD-L1 inhibition, developed immune-related toxicities that have completely recovered prior to start of treatment in this trial, the intrathecal Nivolumab application can be performed.

Events	Action
Non treatment-related and treatment-related toxicities from level CTCAE grade 3 (not neurological)	Continuation of treatment should be stopped for at least 2 weeks. In case of a dose pausing of more than 3 cycles (6 weeks), the study must be discontinued for the subject and the sponsor should be contacted to discuss further steps.
Treatment should not be given to patients with fever (>38°C) or suffering from acute uncontrolled infections.	In such case, treatment should be postponed or omitted until the patient fully recovered. The Sponsor should be contacted to discuss the schedule for resumed doses.

Table 4 Temporary suspension

In this small phase I trial with Part I and Part II, there is no discontinuation rule based on statistical efficacy parameters. The discontinuation rules are based on Adverse Events and safety considerations.

Dose Limiting Toxicities are defined as following:

- CTCAE grade 4 or above Adverse Events related to the IMP
- Neurological CTCAE grade 2 and 3 Adverse Events related to the IMP that have a recommendation of permanently discontinuation of immunotherapies according to the "NCCN Guideline on Management of Checkpoint Inhibitor related Toxicities" (NCCN, 2021a), see also Appendix III

For the permanent discontinuation of study treatment with inthrathecal Nivolumab see section 7.6 and for discontinuation of the complete clinical trial see section 7.7 and 7.8.

6.6.1. Dose Modification Part I:

In the absence of treatment-related toxicities the dosage will be escalated stepwise after each cohort (see figure 1 and 4).

The dosage for all treatment visits in Cohort 1 will be 20 mg for the first 3 (or 6) patients.
The dosage for all treatment visits in Cohort 2 will be 30 mg for the first 3 (or 6) patients.
The dosage for all treatment visits in Cohort 3 will be 40 mg for the first 3 (or 6) patients.
The dosage for all treatment visits in Cohort 4 will be 50 mg for the first 3 (or 6) patients.

6.6.2. Dose Modification Part II

The dosage in the expansion part (Part II) will be a fixed dosage for all patients depending on the results from Part I (i.e. 20 mg, 30 mg, 40 mg or 50 mg). If no Nivolumab dose with acceptable Adverse Events is found in Part I, the trial will be closed (see section 7.6).

6.7. Special warnings and precautions for use of Nivolumab (see SmPC) Monotherapy

For suspected immune-related adverse reactions (amongs others: Immune-related pneumonitis, Immune-related colitis, Immune-related hepatitis, Immune-related nephritis and renal dysfunction, Immune-related endocrinopathies, Immune-related skin adverse reactions) adequate evaluation should be performed to confirm aetiology or exclude other causes. Based on the severity of the adverse reaction, Nivolumab should be withheld and corticosteroids administered. If immunosuppression with corticosteroids is used to treat an adverse reaction, a taper of at least 1-month duration should be initiated upon improvement. Rapid tapering may lead to worsening or recurrence of the adverse reaction.

Non-corticosteroid immunosuppressive therapy should be added for the treatment of immune-related adverse events if there is worsening or no improvement despite corticosteroid use.

Nivolumab should not be resumed while the patient is receiving immunosuppressive doses of corticosteroids or other immunosuppressive therapy. Prophylactic antibiotics should be used to prevent opportunistic infections in patients receiving immunosuppressive therapy.

Nivolumab must be permanently discontinued for any severe immune-related adverse reaction that recurs and for any life-threatening immune-related adverse reaction.

A short-term application of corticosteroids as preventive medication (e.g. as antiemetic prophylaxis) for systemic treatment regimens is allowed in this trial given the advanced stage of disease that is treated in this trial. The administration of dexamethasone > 2 mg/day as a preventive medicinal product requires prior official authorization by the sponsor. The indication and dosage of such a corticosteroid use has to be defined and documented very carefully, however, and needs to be discussed with the sponsor before its application.

7. Study Procedures and Examination Method

This trial will consist of the following consecutive phases (see figure 2 and 3):

- Screening Visit (-14 to -1 days)
- Treatment Visits (V1-V6) with IT administration of PD-1 every 14 days (+/- 3 days)
- Safety Visits 7 days (+/- 3 days)
  - after Visit 3 (or after the last intrathecal dosage within the scope of the study)
  - after Visit 6 (or after the last intrathecal dosage within the scope of the study)
- Follow-up Visits (1-4)
  - every 4 weeks days after last dose

During this trial, we will evaluate the following elements (all details will be outlined below)

- Clinical examination: neurological, neurocognitive assessment, monitoring of increased pain sensation (NANO scale, MoCa, allodynia, sympathetic skin reaction)
- Longitudinal CSF assessments at each treatment visit prior to IT administration
- MRI as assessed per revised RANO and LANO scorecard (Le Rhun et al., 2019)
- Patient-reported outcome (ECORTC QLQ-C30/EORTC QLQ-BN20/BN20, Distress-thermometer (Mehnert et al., 2006)
- Collection of CSF and PBMC biosamples (cfDNA, immunopeptidome, cytokines) for translational endpoints. For Part I, the PBMC sample will be collected only at the lead center. In Part II, PBMC samples will be also collected at external sites.

The AE assessment includes clinical and imaging signs (per LANO, see Appendices I). Please note that imaging and the assessment will be every 12 weeks per standard of care. Yet, if any of the above-mentioned clinical features occur, MR imaging will be performed immediately to investigate the following conditions:

- Increased subarachnoid or ventricular nodules in the brain/spine
- Worsening of leptomeningeal linear enhancement in the brain/spine
- Worsening of hydrocephalus in the brain
- Progression of metastases in the CNS parenchyma (if patient had metastases in the CNS parenchyma at trial entry)

All details on time-points and trial procedures are listed in Table 1.

7.1. Study Entry

7.1.1. Patient’s Informed Consent

The subject has to be informed both in writing and verbally by the investigator before any study-specific procedure is performed. Each patient will be informed about the modalities of the clinical study in accordance with the provided patient information. The patient is given sufficient time to consider participation in the clinical trials and ask for additional advice if needed. Informed consent from the patient will be obtained using a form approved by the responsible ethical committee (EC). The patient and informing investigator must each personally date and sign the informed consent form with an integrated declaration on data privacy protection on the same day. The original signed documents will be part of the investigator’s site file and retained with it and a copy included the insurance policy of the trial will be handed to the patient. The informed consent process is documented in the patient records. For participating centers with DKTK Clinical Communication Platform (CCP) infrastructure, patients can optionally consent to secondary use for data and biospecimens for further research purposes. Consent to the scientific use of their data and biomaterials for further medical questions is voluntary. This is independent of participation in the IT-PD1 study

7.1.2. Screening

Screening will be performed within 14 days prior to V1 intrathecal administration of Nivolumab.
After having signed the informed consent, patients will undergo all assessments listed below:

- Eligibility criteria inclusion and exclusion criteria
- Physical examination
- Demographics
- Weight, height, temperature
- History of underlying primary solid tumor: tumor type, histology
- Collection of previous chemotherapy and radiotherapy treatments
- Collection of current status of systemic disease, presence of parenchymal metastases in the brain and spinal cord
- Assessment of prior and concomitant medication
- Vital signs (blood pressure, heart Rate)
- Karnofsky performance score

- Clinical laboratory assessments (approx. 20 mL blood, hematology, chemistry coagulation at screening should be repeated within 7 days prior first dose, if done earlier):
  - Hematology: haemoglobin, haematocrit, erytrocytes/RBC (abs.), platelets (abs.), leucocyte/WBC (abs.), neutrophils/ANC (abs.), eosinophils (abs.), basophils (abs.), monocytes (abs.), lymphocytes/ALC (abs.)
  - Blood chemistry/electrolytes: albumin, sodium, potassium, calcium, magnesium, chloride, glucose, alkaline phosphatase, ALT, AST, bilirubin direct, Bilirubin total, creatinine, urea
  - Hormonal function: cortisol, TSH, free T4
  - Coagulation panel: prothrombin time, partial thromboplastin time, INR
  - HBV, HCV and HIV Serology
- Blood collection for PBMC preparation (approx. 50 ml blood)
- Urine analysis: urine dipstick
- test for SARS-CoV-2 (optional) Pregnancy testing of WOCBP at screening must be repeated within 72 hours prior to the 1' administration of IT Nivolumab
- Collection of paraffin tumor blocks or 15-20 unstained slides from the primary tumor and optional from metastases. Please note that slides or blocks do NOT have to be present at the timepoint of inclusion or enrolment of patient but does need to be shipped before end of the trial.
- Neurological examination (NANO scale)
- Allodynia monitoring and increased pain sensation: The occurrence of allodynia and increased pain sensation will be monitored clinically (as integral part of the medical history) and by the following neurological examinations: Necessary is the assessment of sympathetic skin response (per standard of care) before the start of intrathecal Nivolumab and after 6 applications. Optional and only if applicable and available at the study center, a quantitative sensory testing (QST) can be performed for those sites with available QST equipment.
- 12-lead electrocardiogram
- Optional: date of planned intraventricular catheter implantation (procedure conducted per clinical indication and as part of the patient`s routine clinical management)
- Results from cerebral and spinal MRI (using items of the RANO and LANO scorecard) during clinical routine. MRIs has to be done within 14 days prior to first IT administration of Nivolumab

- CSF parameters: Neuropathological assessment: cell count, atypical cells, glucose, protein, IL-6, lactate (5ml liquid)
- CSF collection for biomarker analysis/liquid biopsy (separate tube, approx. 5ml liquid)
- Patient-reported outcome assessments (Distress-thermometer, EORTC QLQ 30 & BN20)

The investigator will review all information obtained from the screening procedures.
Screening failures; i.e. screened patients not in compliance with all criteria are to be excluded and the reason will be recorded in the patient records and in the Subject Screening Log. Information of patient's trial participation can be provided to the patient's general practitioner if the patient agreed.
Screening failures are defined as patients who consent to participate in the clinical trials, but are not subsequently treated with at least one IT administration of PD-1 antibody.
Patients who have laboratory abnormalities may be rescreened at the discretion of the investigator.
All patients who receive at least one intrathecal administration of Nivolumab, will be enrolled into the intention-to-treat population.

7.1.3. Enrollment

Patients who fulfil all the inclusion criteria and none of the exclusion criteria will be eligible to participate in the trial. PI confirms and documents that the subject fulfils all criteria for eligibility and can be enrolled. Due to the present study design and the staggered inclusion of subjects in the four cohorts of Part I, each participating center must wait for approval of the sponsor for enrollment. The subject may only be entered into the database once the enrollment has been approved. The register formular must be sent by fax (+49 7071/2925080) or email (zks-pm@med.uni-tuebingen.de) to the ZKS. Approval will be communicated via fax or email. The ZKS registers the patient via the electronical Case Report Form database (secutrial®) and informs the site of the Subject ID. The eCRF system creates a unique subject number for identification. The unique subject number for identification purposes will be assigned to the patient in order to maintain his/her anonymity. The subject number will be used for the patient throughout the study. The study center documents the enrollment in the Subject Enrollment Log.

If the participant agrees to the optional use of data and biomaterials for further scientific research (this is only possible for sites with a DKTK Clinical Communication Platform (CCP) infrastructure), the personal data will be coded again at the local site. This is a separate additional procedure, independent of the pseudonymization carried out as part of the study. This encrypted data can then be transmitted to the DKTK.

7.1.4. Concomitant Medication and Treatments

Relevant additional medications and treatments administered to the subjects on entry to the trial or at any time during the trial are regarded as concomitant medications and treatments and must be documented on the appropriate pages of the eCRF.

If subjects need to undergo palliative radiotherapy to the CNS for LMD or metastases in the brain parenchyma or other metastases during the treatment phase of this trial, a tumorboard recommendation for the radiotherapy technique needs to be documented and the radiation plans need to be provided. The intrathecal Nivolumab application might continue as was the case in the case report (see section 1.3). The exact time points of radiation and intrathecal application need to be discussed with the sponsor.

According to the SmPC the treatment with the PD-1 can lead to hypersensitivity to the active substance or to any of the excipients:

- Sodium citrate dihydrate
- Sodium chloride
- Mannitol (E421)
- Pentetic acid (diethylenetriaminepentaacetic acid)
- Polysorbate 80
- Sodium hydroxide (for pH adjustment)
- Hydrochloric acid (for pH adjustment)
- Water for injections.

7.1.5. Prohibited Concomitant Medications and Treatments

The following concomitant medications and treatments are prohibited during the trial:

- permanent Dexamethasone > 2 mg/day or equivalent unless required to treat an adverse event or as preventive medication (e.g. antiemetic prophylaxis) in the context of a systemic treatment regimen (Note: the administration of dexamethasone > 2 mg/day as a preventive medicinal drug requires prior official authorization by the sponsor)
- Application of any other compound via intrathecal delivery
- Live or attenuated vaccines
- Immunostimulatory or immunosuppressive medications, including herbal remedies
- Any herbal remedies that might interfere with major organ function

All other medications are permitted.

7.1.6. Interaction with other medicinal products and other forms of interaction

Nivolumab is a human monoclonal antibody, as such pharmacokinetic interaction studies have not been conducted. As monoclonal antibodies are not metabolised by cytochrome P450 (CYP) enzymes or other drug metabolising enzymes, inhibition or induction of these enzymes by co-administered medicinal products is not anticipated to affect the pharmacokinetics of Nivolumab.

7.1.6.1. Other forms of interaction Systemic immunosuppression

The use of systemic corticosteroids and other immunosuppressants at baseline, before starting Nivolumab, should be avoided because of their potential interference with the pharmacodynamic activity. However, systemic corticosteroids and other immunosuppressants can be used after starting Nivolumab to treat immune-related adverse reactions. Corticosteroids might also be used in the context of systemic treatment systemic treatment regimens, e.g. as antiemetic prophylaxis. This scenario (> 2 mg/day as a preventive medicinal) needs to be discussed with the sponsor and has to be documented very carefully. These applications of corticosteroids are allowed in this trial because preliminary results show that systemic immunosuppression after starting Nivolumab treatment does not appear to preclude the response on Nivolumab.

7.2. Treatment Phase

This Study will consist of the following consecutive phases: Screening Phase, IT administration treatment, end of safety and Follow-up. Patients will undergo 6 cycles each 14 days in duration and a Safety Visit 7 days after the 3rd dosage and 7 days after the 6th dosage. Time-points and trial procedures are listed in Table 1.

In case of pandemic situations, the Sponsor hereby confirms to adhere according to regulary recommendations.
Before the first IT administration of PD-1 antibody, the inclusion and exclusion criteria must be checked by the investigator. Radiation therapy due to clinical indications must have been finished 2 weeks before the first study treatment administration.
It is predefined to perform the intrathecal administration of PD-1 antibody Nivolumab via an Ommaya reservoir or another intraventricular catheter. Please refrain from repetitive lumbar puncture for intrathecal administration because this increases the burden for patients and does not guarantee an equal distribution and adequate dosage of the IT drug. Rinsing with saline solution is necessary.

Ready-to-use commercial Nivolumab will be obtained from the local pharmacy.

7.2.1. Description Treatment Visits 1-6

The IT administrations will be performed every 14 days.

The following assessments will be performed prior to the IT administration:

- Physical examination
- Weight
- Temperature
- Vital signs (blood pressure, heart rate)
- Karnofsky performance score
- Assessment of AE, toxicity
- Concomitant medication
- Adverse Events recording
- Clinical laboratory assessments (approx. 20 ml blood):

  - Hematology: haemoglobin, haematocrit, erytrocytes/RBC (abs.), platelets (abs.), leucocytes/WBC (abs.), neutrophils/ANC (abs.), eosinophils (abs.), basophils (abs.), monocytes (abs.), lymphocytes/ALC (abs.)
  - Blood chemistry/electrolytes: albumin, sodium, potassium, calcium, magnesium, chloride, glucose, alkaline phosphatase, ALT, AST, bilirubin total, bilirubiun direct, creatinine, urea
  - Hormonal function: cortisol, TSH, free T4
  - Coagulation panel: prothrombin time, partial thromboplastin time, INR
- Blood collection for PBMC preparation (approx. 50ml blood)
- Urine analysis: urine dipstick
- Covid-19 Quick Test (optional)
- Pregnancy testing of WOCBP
- CSF: for cytology, neuropathological assessment: cell count, atypical (tumor) cells, glucose, protein, IL-6, lactate (approx. 5ml)
- CSF collection for biomarker analysis/liquid biopsy (separate tube, approx. 5ml)
- Neurological examination (NANO scale)
- Mini Mental Status (MMSE)
- Montreal Cognitive Assessment (MoCa)
- Allodynia monitoring and increased pain sensation: The occurrence of allodynia and increased pain sensation will be monitored clinically (as integral part of the medical history) and by the following neurological examinations: Necessary is the assessment of sympathetic skin response (per standard of care) before the start of intrathecal Nivolumab and after 6 applications. Optional and only if applicable and available at the study center, a quantitative sensory testing (QST) can be performed for those sites with available QST equipment.
- IMP application via an intraventricular catheter
- Patient-reported outcome assessments (Distress-thermometer, EORTC QLQ 30 & BN20)

The intrathecal administration of Nivolumab will be performed over 5 minutes. After the first IT administration at V1 the patient should remain 6 hours at the center for Follow-up monitoring. Unless the center decides that from a clinical point of view an inpatient admission is necessary. For all subsequent intrathecal administrations, only 1 hour of Follow-up is required.

7.3. Safety Visit 1 and 2 for Subjects

7 days after the 3rd (V3) dose and 7 days after the 6th (V6):

- Physical examination
- Weight
- Temperature
- Vital signs (blood pressure, heart rate)
- Karnofsky performance score
- Concomitant medication
- Adverse Events recording
- Clinical laboratory assessments (approx. 20ml blood):
  - Hematology: haemoglobin, haematocrit, erytrocytes/RBC (abs.), platelets (abs.), leucocytes/WBC (abs.), neutrophils/ANC (abs.), eosinophils (abs.), basophils (abs.), monocytes (abs.), lymphocytes/ALC (abs.)
  - Blood chemistry/electrolytes: albumin, sodium, potassium, calcium, magnesium, chloride, glucose, alkaline phosphatase, ALT, AST, bilirubin total, bilirubiun direct, creatinine, urea
  - Hormonal function: cortisol, TSH, free T4
  - Coagulation panel: prothrombin time, partial thromboplastin time, INR
- Blood collection for PBMC preparation (approx. 50ml blood)
- Urine analysis (stick)
- Pregnancy testing of WOCBP
- Covid -19 Quick Test (optional)
- CSF: for cytology, neuropathological assessment: cell count, atypical (tumor) cells glucose, protein, IL-6, lactate. (approx. 5 ml)
- CSF collection for biomarker analysis/liquid biopsy (separate tubes, approx. 5 ml)
- Neurological examination (NANO scale)
- Mini Mental Status (MMSE)
- Montreal Cognitive Assessment (MoCa)
- Allodynia monitoring and increased pain sensation: The occurrence of allodynia and increased pain sensation will be monitored clinically (as integral part of the medical history) and by the following neurological examinations: Necessary is the assessment of sympathetic skin response (per standard of care) before the start of intrathecal Nivolumab and after 6 applications. Optional and only if applicable and available at the study center, a quantitative sensory testing (QST) can be performed for those sites with available QST

    - Results from cerebral and spinal MRI (using items of the RANO and LANO scorecard), during clinical routine
    - Patient-reported outcome assessments (Distress-thermometer, EORTC QLQ 30 & BN20)

If patient has a progressive disease, a Dose Limiting Toxicity (DLT), or withdrawn before completion of IMP Visit 3 or 6, the Safety Visit should be performed. Patient will continue with Follow-up.

7.4. Follow-up 1-4 and Long-time Follow-up

The **Follow-up** phase will start four weeks after the last dose and will continue up to 4 Follow-up Visits in total.

- Survival Status
- Date and report of all MRIs (as performed per standard of care)
- Optional at first Follow-up Visit: Blood collection for PBMC preparation (approx. 50 ml blood)
- Optional at first Follow-up Visit: CSF collection of:
    - CSF: for cytology, neuropathological assessment: cell count, atypical (tumor) cells, glucose, protein, IL-6, lactate. (approx. 5 ml)
    - CSF collection for biomarker analysis/liquid biopsy (separate tubes, approx. 5 ml)
- Neurological examination (NANO scale)
- Mini Mental Status (MMSE)
- Montreal Cognitive Assessment (MoCa)
- Report including RANO and LANO assessment of brain MRIs during clinical routine
- Allodynia monitoring and increased pain sensation: The occurrence of allodynia and increased pain sensation will be monitored clinically (as integral part of the medical history) and by the following neurological examinations: Necessary is the assessment of sympathetic skin response (per standard of care) before the start of intrathecal Nivolumab and after 6 applications. Optional and only if applicable and available at the study center, a quantitative sensory testing (QST) can be performed for those sites with available QST equipment.
- Patient-reported outcome assessments (Distress-thermometer, EORTC QLQ 30 & BN20)

The **Long-time Follow-up** takes place outside the study for at least every 6 months for 1 year, unless the patient withdraws his consent. The observation in the Long time FU (e.g. by telephone) included:

- survival status,
- progression
- further therapy

7.5. Assessment of Efficacy and Safety

Safety assessments:

AEs and SAEs will be collected from the time of signing the Informed Consent, including those SAEs considered to be associated with protocol-specified procedures (such as MRI with contrast or CSF collection at screening). Safety assessments during the treatment phase comprise complete and continuous AE recording and SAE reporting until Follow-up 4 after last IMP administration during the trial.

Clinical assessments:

Physical/neurological examination, vital signs, KPS, haematology, biochemistry, thyroid function, ECG, coagulation, urine analysis, cerebrospinal fluid analysis are assessed on a regular basis.

Pregnancy test (if applicable) will be performed before study entry and treatment.

Immunomonitoring:

Immunogenicity analyses (detection of vaccine-induced immune responses) will be performed

with peripheral blood mononuclear cells (PBMCs) isolated from blood samples. For Part I, the PBMC sample will be collected only at the lead center and for Part II, the PBMC sample is taken at all sites. PBMCs will be frozen according to standardized procedures. CSF will be collected for Part I and II in all participating study sites. CSF will be collected in Streck tubes and will then be processed and stored frozen (as outlined in the lab manual). Details are outline in a dedicated immunogenicity assessment manual. Sample collection is being performed until end of the last Safety Visit and optional at the first Follow-up Visit. All samples shall be sent to

the sponsor (Universitätsklinikum Tübingen, Zentrum für Neurologie, Abteilung Neurologie mit interdisziplinärem Schwerpunkt Neuroonkologie, zHd Studienzentrale Neuroonkologie, Hoppe Seyler Str. 3, 72076 Tübingen) at regular intervals.

Assessment of tumor response and survival data:

All imaging assessments as indicated per clinical routine (including MRI) shall be performed according to standard intervals and procedures established at the participating sites. Tumor response from all available MRI scans taken after start of treatment will be evaluated in this study using NANO for the clinical neurological assessments, RANO (Lin et al., 2015) and the Modified LANO scale Leptomeningeal Assessment in Neuro-Oncology (Le Rhun et al., 2019). The diagnostic criteria in NANO (Nayak et al., 2017) go beyond LANO (Le Rhun et al., 2019). NANO is a standardised neurological examination, and LANO is an assessment specifically for meningeosis neoplastica. We would therefore like to use both procedures in this study.

Baseline for assessment of tumor response shall be an MRI scan taken within 2 weeks before the first day of the first cycle.

7.6. Premature termination of clinical trial for a trial subject

Reasons for premature termination of trial for an individual trial subject are:

- Death
- CTCAE grade 4 or above Adverse Events related to the IMP
- Neurological CTCAE grade 2 and 3 Adverse Events related to the IMP that have a recommendation of permanently discontinuation of immunotherapies according to the “NCCN Guideline on Management of Checkpoint Inhibitor related Toxicities” (NCCN, 2021a), see also Appendix III
- Clinical and MRI signs of tumor progression in the CNS. A tumor progression outside the CNS does not necessarily impact the study treatment (i.e patients remain eligible for further study treatments). The local investigator may decide to discontinue the treatment if the patient`s clinical condition does not allow further study treatments.
- Major protocol violation
- If, in the investigator’s opinion, continuation of the trial would be detrimental to the subject’s well-being
- For women, in case of pregnancy

- Noncompliance
- Decision of patient, i.e. withdrawal of informed consent. If a patient retracts the consent for study treatment, he/she will be asked to stay in the Follow-up to ensure clinical monitoring of patient and Follow-up data. If patient is not willing to do so, he/she will be completely excluded from the trial upon consent withdrawal

The local investigator decides about withdrawal of subjects from trial treatment in case of occurrence of criteria mentioned above. In all cases, the reason for withdrawal must be recorded in the CRF and in the subject's medical records. In case of withdrawal of a subject at his/ her own request, the reason should be determined and documented.

All examinations scheduled for the last trial day will be performed and documented as far as possible, subject to the consent of the patient. These subjects will enter the regular Follow-up of the trial, unless the subject has withdrawn his/her consent to any further study-related procedure. If a subject may / will be withdrawn from all trial-related procedures (including Follow-up Visits) (e.g. at his/her own request), this will not result in any disadvantages for the patient.
Premature termination should be avoided. In case of a premature termination of therapy, reasons/circumstances and if applicable the final status have to be documented. If the patient does not withdraw the consent for further Follow-up, he/she should be followed-up as planned.

7.7. Premature closure of a trial site

Premature closure of a trial site has to be considered if:

- The conduct of the study is not compliant with the protocol or the legal regulations, or
- The data quality is not sufficient

The premature closure of a site will be decided by the sponsor.

Site principal investigators may terminate his/her participation in the study. If this occurs they should provide a written statement of the reasons for terminating participation and should provide the sponsor with all available and up-to-date study data.

The sponsor may also decide to terminate participation of an investigator or study center for the following reasons:

- Breach of agreement
- Serious non-compliance to protocol or the legal regulations
- Insufficient patient recruitment

If a participating center closes, or is closed, prior to termination of the whole trial, the sponsor expects that data from patients already entered into the trial will be reported as per protocol. Details on further treatment and Follow-up of patients on study have to be discussed with the site principal investigator.

7.8. Premature termination of the trial

The trial may be prematurely terminated, if in the opinion of the sponsor and coordinating investigator there is sufficient reasonable cause for terminating the study as a whole. Written notification documenting the reason for study termination will be provided to the investigators.

In case of the following situations, a premature termination of the trial has to be considered:

- An unacceptable profile or incidence rate of Adverse Events revealed in this or any other study in which PD-1 of this trial is administered
- If dose limiting-toxicities has been identified in more than 1 of 6 subjects in cohort I in Part I (20 mg) the study will be terminated.
- If no Nivolumab dose with acceptable Adverse Events is found in Part I, the trial will be closed. Furthermore, during Part I and II, the DSMB and/or the leading/coordinating investigator (as the designee of the sponsor) can stop the trial transiently or permanently for safety reason.
- New insights from other trials
- Insufficient recruitment rate
- Any other factor that in the view of the sponsor constitutes an adequate reason

The DSMB will monitor the study conduct and the safety aspects of the trial on a regular basis, and will give recommendations to the coordinating investigator/ the sponsor. The sponsor will then decide on the actions to be taken. According to the German drug law (§42a), the trial may be suspended or prematurely terminated by decision of the competent authority (PEI).

7.9. Plan for Treatment or Care after End of Study

Patients without signs for progression and who tolerate the intrathecal treament will have the possibility to continue the treatment outside the IT-PD1 trial after consultation with their local principal investigator and the sponsor. Patients will be monitored and further treated according to the current clinical guidelines.

8. Quality control and Quality assurance

8.1. Risk-based approach

During protocol development, processes and data that are critical to ensure human subject protection and the reliability of trial results were identified.

The identified risks were evaluated against existing risk controls by considering:

- The likelihood of errors occurring
- The extent to which such errors would be detectable
- The impact of such errors on human subject protection and reliability of trial results.

In case of unacceptable risks, risk reduction activities were defined and incorporated e.g. in the protocol, monitoring plan and agreements.

Results will be communicated to those who are involved in or affected by such activities.

The sponsor periodically reviews risk control measures to ascertain whether the implemented activities remain effective and relevant, considering emerging knowledge and experience.

8.2. Monitoring

Monitoring for this study is provided by the Zentrum für Klinisch Studien Tübingen (ZKS Tübingen). The monitoring will be conducted according to ZKS Tübingen internal Standard Operating Procedures (SOPs) and a dedicated monitoring manual for the study. The monitoring timelines include, for all centers, initiation visit, regular monitor visits during the course of the trial as well as a close out visit. All investigators agree that the monitors regularly visit the trial site, assure that the monitors will receive appropriate support in their activities and will have access to all trial-related documents.
Details of the monitoring's conduct will be specified in a Monitoring Manual.

8.3. Audits/ Inspections

In addition to the monitoring activities, audits can be conducted by the sponsor or assigned auditors. These audits may include checking the whole course of the study, documentation, trial center, investigators and the monitor.

The competent regulatory authorities may also conduct inspections.

With his/her participation in the study, the investigator agrees to support the activities of the auditor/inspector, provide her/him with direct access to the source documents, study documentation and give her/him the opportunity to audit/inspect the study site, laboratory facilities, storage of the investigational product, etc.

8.4. Documentation: Collection, Handling, Storage and Archiving of Data

8.4.1. Case Report Form

The trial Case Report Form (CRF) is the primary data collection instrument for the trial. All data requested on the CRF must be recorded. All missing data must be explained.

For this project, electronic Case Report Forms (eCRFs) will be used. The Clinical Data Management System [“SecuTrial”] will be used for data capture, processing and storage of study data. Data entry is performed at the investigational site by clinical staff after having received training and a user manual for the electronic CRF. Training and the user manual will detail procedures to be followed in case of technical problems. Queries resulting from edit checks and/or data verification procedures will be posted electronically in the eCRF.
The Clinical Trial Data Management System (CDMS) is validated and changes are tracked via an audit trail.

The correctness of entries in CRFs will be confirmed by dated signature of an authorized investigator. The Principal investigator or a delegated investigator is responsible for ensuring that all sections of the CRF are completed correctly. The completeness of entries (DEC) in CRFs to vertified against source data will be confirmed by an authorized investigator or delegated member of the investigater team. The Principal investigator has to verify the CRFs via dated signature/electronic signature at regular intervals and after completion of the CRF.

8.4.2. Source Data

Source data is all information, original records of clinical findings, observations or other activities in a clinical trial necessary for the reconstruction and evaluation of the trial. Source data are contained in source documents. Examples of these original documents and data records include: hospital records, clinical and office charts, laboratory notes, memoranda, patients’ diaries or evaluation checklists, pharmacy dispensing records, recorded data from

automated instruments, copies or transcriptions certified after verification as being accurate and complete, x-rays, CTs, MRIs, ultrasound reports, patient files, and records kept at the pharmacy, at the laboratories, and at medico-technical departments involved in the clinical trial.

8.4.3. Data Handling

Authorized clinical staff at the investigational site will enter the data into the eCRF using an access controlled, audit-trailed, ICH/GCP compliant, validated system. Entered data will be subjected to plausibility checks directly implemented in the CRF, monitoring and medical review. Implausible or missing data will be queried. Database lock will be performed after completion of data entry, data cleaning and a final data review.

8.4.4. Storage and Archiving of Data

According to the EU Clinical Trial Regulation 536/2014 all essential trial documents (e.g. CRF) will be archived for at least 25 years after the trial termination. The investigator(s) will archive all trial data (source data and Investigator Site File (ISF) including subject identification list and relevant correspondence) according to the Guideline ICH GCP (E6) and to local law or regulations.

9. Statistical Analyses

9.1. Study Population Definition

9.1.1. Sample Size and Power Consideration

This trial includes a minimum of 32 evaluable patients (12 Part I, 20 Part II) and a maximum of 49 evaluable patients (24 Part I, 25 Part II). Between 12 and 24 patients will be included in the dose finding phase Part I with four doses (20, 30, 40, 50 mg) and 20 evaluable patients will be included in the expansion phase using the MTD identified in the 3+3 phase. With 20 evaluable patients in can be shown, that DLT is smaller than 33% assuming a true DLT of maximal 7% (exact binomial test, type 1 error = 0.025 one-sided, power = 80%, H0: DLT = 33%, H1: DLT < 33%, assumed alternative: DLT ≤ 7%).

9.2. Analysis Primary Variables

The primary outcome variable is dose limiting toxicity (DLT). In Part I of the trial, a 3+3 design will be applied and the maximum tolerable dose (MTD) for the expansion phase (Part II) will be determined according to the criteria of this design (maximum dose with zero DLTs in three patients or one DLT in the first three patients and zero DLTs in patients four to six). The MTD determined in Part I will be used in the expansion phase (Part II) of the trial in 20-25 patients. An exact one-sided binomial test will be performed with H0: DLT = 33%. Additionally, an exact two-sided 95% confidence interval will be given for DLT. The primary analysis population will be a modified Intent to Treat population (mITT) which includes all patients receiving at least one treatment dose.

9.3. Analysis Secondary Variables

Safety

The statistical analysis of the secondary endpoints will be done in a descriptive manner. No statistical tests with confirmatory aim are planned. The safety will be described by absolute and relative frequencies using CTCAE V5.0-scoring.

Efficacy

The overall survival within a minimum of 4 months after start of PD-1 treatment (Kaplan Meier estimate including two-sided 95% CI CI of survival probability after four months) will be the secondary endpoint for efficacy.

9.4. Safety Interim Analysis

A safety analysis for each cohort in Part I will be undertaken to determine the maximum tolerated fix dosage to proceed with Part II of the study. The data to be evaluated by the DSMB will include (report):

- AEs/ADRs, and SAEs
- Review and, if necessary, assessment of (S)AE relatedness to IMP
- actions taken
- duration and outcome of (S)AE

The DSMB decision will be documented in a TMF. The information will be distributed to the study sponsor, all investigators/trial sites and the ZKS Department Pharmakovigilanz for information. After safety has been confirmed, the study will continue enrolment as planned.

9.5. Stopping Rules

The sponsor has the right to terminate the trial prematurely if there are any relevant medical or ethical concerns, or for reasonable administrative reasons. If such action is taken, the reasons for terminating the trial have to be documented in detail. All patients who are not considered end of study must undergo a final examination, which must be documented.

Criteria for premature termination of the study as a whole are listed in section 7.8 “Premature termination” of the trial:

The Sponsor has to be informed without delay if any investigator has ethical concerns.

9.6. Drop-out

If a subject discontinues treatment prior to Safety Visit 1 due to disease progression, death, IC withdrawal or other reasons, but without Dose Limited Toxicity (DLT), the subject will be counted as a drop-out for this cohort and shall be replaced by another subject for the DSMB safety assessments. However, the drop-out will be documented in the intention to treat population and in all further statistical analyses (except for DLT), as this naturally provides relevant information on efficacy.

9.7. Biometric Report

The biostatistical center will participate in scientific publications. Additionally, a concise statistical report including data listings, parameters and graphical displays will be provided.

10. Safety

10.1. Definition of Adverse Events, Serious Adverse Events and Side Effects

10.1.1. Adverse Events

Any untoward medical occurrence in a patient or clinical investigation subject administered a pharmaceutical product and which does not necessarily have a causal relationship with this treatment. An adverse event (AE) can therefore be any unfavorable and unintended sign (including an abnormal laboratory finding), symptom, or disease temporally associated with the use of a medicinal (investigational) product, whether or not related to the medicinal (investigational) product.

An AE may be:

- New symptoms/ medical conditions
- New diagnosis
- Changes of laboratory parameters
- Diseases and medical consequences of an accident
- Worsening of medical conditions/ diseases existing before clinical trial start
- Recurrence of disease
- Increase of frequency or intensity of episodical diseases

A pre-existing disease or symptom will not be considered an adverse event unless there will be an untoward change in its intensity, frequency or quality. This change will be documented by the investigator.

In general, abnormal laboratory findings or clinical events without clinical significance (based on the investigator´s judgement) should not be recorded as AEs.

Surgical procedures themselves are not AEs; they are therapeutic measures for conditions that require surgery. The condition for which the surgery is required may be an AE. Planned surgical measures permitted by the clinical trial protocol and the condition(s) leading to these measures are not AEs, if the condition leading to the measure was present prior to inclusion into the trial.

AEs are classified as "non-serious" or "serious".

10.1.2. Serious Adverse Event and Serious Adverse Reaction

A serious adverse event (SAE) is one that at any dose:

- Results in death.
- Is life-threatening (the term life-threatening refers to an event in which the subject was at risk of death at the time of event and not to an event which hypothetically might have cause death if it was more severe).
- Requires subject hospitalization or prolongation of existing hospitalization.
- Results in persistent or significant disability/ incapacity.
- A congenital anomaly / birth defect.
- Is medically significant (e.g. suspected transmission of an infectious agent via medicinal product) Moreover there are other situations - such as important medical events that may not be immediately life threatening or result in death or hospitalisation but may jeopardize the patient or may require intervention to prevent one of the other outcomes listed above.

Important medical event [ICH E2A; EMA/155528/2018]: Examples of such events are intensive treatment in an emergency room or at home for allergic bronchospasm; blood dyscrasias or convulsions that do not result in hospitalisation; or envelopment of drug dependency or drug abuse (Important medical event terms list (MedDRA > version 23.1).

10.1.3. Adverse Drug Reaction

An Adverse Drug Reaction (adverse reaction: undesirable effect) is a response to a medicinal product which is noxious and unintended. An unexpected Adverse Drug Reaction (ADR) is a reaction which nature or severity is not consistent with the applicable product information available for the IMP. Expected ADRs are listed in the appropriate reference documents, e.g. Summary of Product Characteristics (e.g. SmPC, Information Sheet for Health Professionals [Fachinformation in Germany]).

10.1.4. Unexpected Adverse Drug Reaction

An unexpected Adverse Drug Reaction (ADR) is a reaction which nature or severity is not consistent with the applicable product information available for the IMP. Expected ADRs are listed in the appropriate reference documents, e.g. Investigator's Brochure; Summary of

Product Characteristics (SmPC [Fachinformation in Germany]). Furthermore, reports which add significant information on specificity or severity of a known adverse reaction are counted as 'unexpected' events. In this study the IMP is not used within the terms of the marketing authorisation. In the absence of SAE data on the use of intrathecal Nivolumab in leptomeningeal disease, all Serious Adverse Events (SAEs) which are drug related are Serious Adverse Reactions (SARs) and will be assessed as unexpected (SUSAR).

10.2. Reporting of Adverse Events/Serious Adverse Events

10.2.1. Period of Observation and Documentation

For the purpose of this trial, the period of observation for collection of Adverse Events extends from the time of signing the Informed Consent until the last Follow-up 4 Visit after the last dose administrated.

All Adverse Events from grade 1 (according to CTCAE V5.0) that occur in the course of this clinical trial regardless of the causal relationship must be monitored and followed up until the outcome is known or no more information is achievable.

10.2.2. Documentation and Reporting of AE/SAE by the Investigator

The investigator must document all Adverse Events that occur during the observation period set in this protocol on the pages provided in the case report form. Additional instructions may be provided in the investigator file and in the case report form itself. The following approach will be taken for documentation:

All Adverse Events (whether serious or non-serious) must be documented on the "adverse event" page of the case report form.

If the adverse event is **serious**, the investigator must complete, in additional a "**serious adverse event report form**" at the time when the serious adverse event is detected. The investigator will document the date when he/she or any employee was first aware of the event. The initial report must be as complete as possible, including reported terms according to "Common Terminology Criteria for Adverse Events (CTCAE)-List" (one term per event), details of the current illness and (serious) adverse event, severity, serious criterium as well as an assessment of the causal relationship between the event and the trial medication. Sending all SAE reports (initial and Follow-up reports), even if they are incomplete, within 24 hours upon receipt to representative of the Sponsor:

Zentrum für Klinische Studien Tübingen (ZKS Tübingen)
Fax.: +49 7071 29 25205
Email: zks-pv@med.uni-tuebingen.de or fax all SAE reports (initial and Follow-up reports)

The investigator should also assess the severity and the causal relationship between the event and the trial medication.

10.2.3. Assessment of Severity and Causality

The investigator will also provide an assessment of the severity of the event according to CTCAE criteria (Version 5.0) and causal relationship between the event and each of the investigational products or trial procedures.

AEs and SAEs should be evaluated for severity according to the following scale:

- Grade 1 - mild event: Causing no limitations of usual activities; the patient may experience slight discomfort.
- Grade 2 - moderate event: Causing some limitation of usual activities; the patient may experience annoying discomfort.
- Grade 3 - severe event: Causing inability to carry out usual activities; the patient may experience intolerable discomfort or pain.
- Grade 4 - life threatening or disabling event
- Grade 5 - death related to event

10.2.4. Relationship of the AEs to the Investigational Medicinal Product

The investigator should also assess the causal relationship between the event and the trial medication to following scale:

Is there a reasonable causal relationship that the AE is related to the study medication?

Y (Yes) There is a reasonable possibility that the IMP/s caused the AE.

N (No) There is no reasonable possibility that the IMP/s caused the AE and other causes are more probable.

If no, other possible causes have to be specified.

10.2.5. Outcome of the Adverse Reactions and actions taken

The information about the outcome of an AE at the time of the last observation should also be reported. The following classification will apply:

Recovered/ Resolved:	All signs and symptoms of an AE disappeared without any sequels at the time of the last interrogation.
Recovering/ resolving:	The intensity of signs and symptoms has been diminishing and/ or their clinical pattern has been changing up to the time of the last interrogation in a way typical for its resolution.
Not recovered/ not resolved:	Signs and symptoms of an AE are mostly unchanged at the time of the last interrogation.
Recovered/ resolved with sequel:	Actual signs and symptoms of an AE disappeared but there are sequels related to the AE.
Fatal	Resulting in death. If there are more than one AE only the adverse event leading to death (possibly related) will be characterized as ‘fatal‘.
Unknown	The outcome is unknown or implausible and the information cannot be supplemented or verified.

The action taken will be assigned to one of the following categories:

Dose not changed	No change in the dose
Dose reduction	Reduction in the dose
Temporary Discontinuation	Temporary discontinuation of treatment
Dose increased	Increase in the dose
Drug withdrawn	Discontinuation of treatment
Unknown	The information is unknown or implausible and it cannot be supplemented or verified.
Not applicable	The question is implausible (e.g. the patient is dead).

10.2.6. Sponsors Assessment of the SAEs

All SAE will be subject to a second assessment by the trial Sponsor/Coordinating Investigator.
The second assessor will fill out a 'Second Assessment Form' for each SAE.
The 'Second Assessment Form' will contain the following information:

- Assessment of relationship between SAE and IMP/ study procedure.
- Assessment of expectedness of SAE.
- Statement if the benefit / risk assessment for the trial did change as a result of SAE.

10.2.7. Follow-up of Initial Report

Information not available at the time of the initial report (e.g. an end date for the adverse event or laboratory values received after the report) must be documented on a "Serious Adverse Event" form with the box "Follow-up" checked under "Report type".
All patients who have Adverse Events, whether considered associated with the use of the investigational products or not, must be monitored to determine the outcome. The clinical course of the adverse event will be followed up according to accepted standards of medical practice even after the end of the period of observation, until a satisfactory explanation is found or the investigator considers it medically justifiable to terminate Follow-up. Should the adverse event result in death, a full pathologist's report should be supplied, if possible. The sponsor will identify missing information for each SAE report and will require follow up information in regular intervals from the investigators until all queries are resolved or no further information can be reasonably expected. All responses to queries and supply of additional information by the investigator should follow the same reporting route and timelines as the initial report.

10.2.8. Exception of reporting

Epileptic seizures need not to be reported as SAEs (complications during these Events remain reportable as a serious Adverse Events, if these complications itself fulfil the SAE criteria).

Tumor progression in the Central Nervous System as assessed per RANO (for parenchymal lesions) and LANO (for LMD) including fatal outcomes should not be reported as an AE or as SAE.

Hospitalizations or prolongation of existing hospitalization as a result of the following causes need not to be reported as SAE (complications during these hospitalisations remain reportable as a serious Adverse Events, if these complications itself fulfil the SAE criteria):

- IMP drug application with planned overnight stay
- planned chemotherapy applications
- because of social cirumstances
- test procedure required in the protocol
- hospitalisations for diagnostic measures only
- technical, practical, or social reasons, in absence of an adverse event
- surgical intervention or other measures and the condition(s) leading to these measures are not AEs, if the condition leading to the measure was present prior to inclusion into the trial.
- planned intraventricular catheter implantation for inthratecal IMP application, in absence of an adverse event
- stay at rehabilitation clinic

If a patient develops signs or symptoms of COVID-19 during hospitalisation, this is documented as an AE. However, if the symptoms meet the the criteria for a Serious Adverse Event (SAE), it has to be reported.

10.2.9. Suspected Unexpected Serious Adverse Reaction (SUSAR)

SAEs have to be assessed by the second asssessor wether they are both suspected, i.e. related to IMP and ‘unexpected’, i.e. the nature and / or severity of which is not consistent with the applicable product information. They are then to be classified as Suspected Unexpected Serious Adverse Reactions (SUSARs).
In case, either the investigator or the second assessor classifies the SAE as related to IMP and the SAE is unexpected as assessed by the second assessor it will be categorized as a SUSAR.
All SUSARs are subject to an expedited reporting by the Sponsor to the the Member States concerned and to all participating investigators. SUSARs will be reported to the EudraVigilance database (in accordance with CTR Annex I D20c).

10.2.10. Expedited Reporting to the Regulatory Authorities

<u>Fatal and life-threatening SUSARs</u>

The European Medicines Agency must be informed by the Sponsor of all fatal or life-threatening SUSARs. This must be done immediately, at the latest seven calendar days after

becoming aware of the minimum criteria for reporting. In all cases, attempts must be made to obtain further relevant information, which must be supplied within a further eight days. Furthermore, if a trial subject dies, this information must be additionally reported. SUSARs will be reported to the EudraVigilance database (in accordance with CTR Annex I D20c).

SUSARs that are not fatal or life-threatening

The European Medicines Agency will be informed without delay by the sponsor or CI of all SUSARs, at the latest within 15 calendar days of becoming aware of the minimum criteria for reporting. Further relevant details will be passed on as soon as possible. SUSARs will be reported to the EudraVigilance database (in accordance with CTR Annex I D20c).
If the information at the time of reporting is incomplete, further information to enable adequate assessment of the case will be requested from the reporter or other available sources.

10.2.11. Examination and Report of Changes in the Risk to Benefit Ratio

Without delay, and at the latest within 15 days of the decision for the need to do so, the Sponsor / CI will inform the Member States concerned of any events or factors that could result in a review of the risk-benefit ratio of the IMP. These consist of especially:

- Individual reports of expected serious ADRs with an unexpected outcome.
- A clinically relevant increase in the rate of occurrence of expected ADRs.
- SUSARs in trial subjects who have already completed the Follow-up period of the clinical trial (”end-of-trial visit”).
- Factors emerging in connection with trial conduct or the development of the IMP that may affect the safety of persons concerned.

10.2.12 Reporting to Data and Safety Monitoring Board

The DSMB will be informed annually of all safety-relevant events by the Sponsor / CI (see Section 1 “Advisory committes”).

10.2.13 Report to the Investigator

The Sponsor / CI will inform investigators of all SUSARs including all relevant further information within the periods set by the authority. If new information becomes known that is different from the scientific information given to the investigator, all investigators will be informed of this by the sponsor.

10.3. Annual Safety Report

Once a year, the Sponsor / CI will supply a report on the safety of trial subjects with all available relevant information concerning patient safety during the reference period to the competent authorities. This report will also be supplied to the responsible ethics committee.
The annual safety report will be compiled according to the corresponding ICH guideline E2F „Development Safety Update Report – DSUR“. The safety report will cover all IMPs used in this study.

10.4. Deviations from the Protocol and serious breaches

Any deviation from the study protocol will be documented in the deviation log outlining the reason of the deviation and it`s consequence for the patient`s safety.
A ‘serious breach’ according EU 536/2014 Artikel 52 means a breach likely to affect to a significant degree the safety and rights of a subject or the reliability and robustness of the data generated in the clinical trial.
Serious protocol or regulatory breaches must be reported immediately by the trial site to the Sponsor via following Email address:

Center for Clinical studies (ZKS)
e-mail: zks-pv@med.uni-tuebingen.de
Fax +49 7071 29 25205

All reported serious breaches will be subject to a second assessment by the trial Sponsor or authorized second assessors.
If according to the second assessment the severity is confirmed the Sponsor will notify the Member States concerned about the serious breach via CTIS without undue delay but not later than seven days of becoming aware of that breach.

10.5. Reporting of Pregnancy

Maternal exposure

If a patient becomes pregnant during the course of the study, treatment has to be discontinued immediately. The outcome of any conception occurring from the date of the first dose until 1 month after the last dose should be followed up and documented.

Pregnancy itself is not regarded as an adverse event unless there is a suspicion that the investigational product under study may have interfered with the effectiveness of a contraceptive medication. Congenital abnormalities/birth defects and spontaneous miscarriages should be reported and handled as SAEs. Elective abortions without complications should not be handled as AEs. The outcome of all pregnancies (spontaneous miscarriage, elective termination, ectopic pregnancy, normal birth or congenital abnormality) should be followed up and documented even if the patient was withdrawn from the study.

If any pregnancy, suspected pregnancy, or positive pregnancy test occurs in the course of the study, this must be reported to ZKS Tübingen, department pharmacovigilance (on behalf of sponsor) immediately by fax (fax-number: + 49 (0)7071 29 25205) or mail (zks-pv@med.unituebingen.de) on the Pregnancy Report Form.

All pregnancies should be followed up and documented, even if the patient was withdrawn from the study, until its outcome (spontaneous miscarriage, elective termination, ectopic pregnancy, normal birth or congenital abnormality). The outcome must be notified immediately by the investigator to the ZKS Tübingen, department pharmacovigilance (on behalf of sponsor) within 24 hours of first knowledge as a Follow-up to the initial report.

For any event during the pregnancy, which meets a seriousness criterion, the Investigator will also follow the procedures for reporting SAEs (complete and send the SAE form to the Sponsor by fax within 24 hours of the Investigator's knowledge of the event).

All neonatal deaths that occur within 30 days of birth should be reported, without regard to causality, as SAEs. In addition, any infant death at any time thereafter that the Investigator suspects is related to the exposure to the study drug/IMPs should also be reported to the Sponsor by facsimile within 24 hours of the Investigators' knowledge of the event.

The same timelines apply when outcome information is available.

If the female is found not to be pregnant, any determination regarding the patient’s continued participation in the study will be determined by the investigator(s).

Paternal exposure

Male patients should refrain from fathering a child or donating sperm during the study and for 5 months following the last dose.

Pregnancy of the patient’s partners is not considered to be an adverse event. However, the outcome of all pregnancies (spontaneous miscarriage, elective termination, ectopic pregnancy, normal birth or congenital abnormality) should if possible be followed up and documented.

The outcome of any conception occurring from the date of the first dose until 3 months after the last dose should be followed up and documented.

Information on pregnancy must be collected on the “Pregnancy Reporting Form”. In order for Sponsor or designee to collect any pregnancy surveillance information from the female partner, the female partner must sign an informed consent form for disclosure of this information.

11. Regulatory Consideration

11.1. Ethical Conduct of Clinical Study

11.1.1. Good Clinical Practice, Declaration of Helsinki and legal Provision

The procedures set out in this trial protocol, pertaining to the conduct, evaluation, and documentation of this trial, are designed to ensure that all persons involved in the trial act according to Good Clinical Practice (GCP) and the ethical principles described in the applicable version of the Declaration of Helsinki.

The Clinical Trial will fulfil all requirements of the German Medicinal Products Act (Arzneimittelgesetz, AMG).

11.2. Subject Information and Informed Consent

Each patient will be informed about the modalities of the clinical study in accordance with the provided patient informed consent (IC). The patient is to be informed both in writing and verbally by the investigator before any study-specific procedure is performed. The patient must be given sufficient time (i.e. >24 h) to decide whether to participate in this comparative study and to ask questions concerning this trial. It must also be made clear to the patient that he / she can withdraw from the study at any time without giving reasons and that he / she will not be in any way disadvantaged for this. The subject must give consent in writing. The patient and informing physician must each personally date and sign the informed consent form with an integrated declaration on data privacy protection, whereby the physician must not sign before the patient. Original signed documents will be part of the investigator's file and retained with it. A copy of the signed informed consent document and study insurance policy must be given to the subject. The documents must be in a language understandable to the subject and must specify who informed the subject. The subjects will be informed as soon as possible if new information may influence his/her decision to participate in the trial. The communication of this information should be documented in the patient chart.

11.3. Insurance

Each patient is insured against any health impairment occurring as a result of participation in the study in accordance with the laws and regulations of the "German Arzneimittelgesetz". The insurance is covered by HDI-Gerling Industrie Versicherung AG, Am Schönenkamp 45, 40599 Düsseldorf, Policy number 57 010311 03013 and valid throughout the conduct of the study

including Follow-ups for each individual patient. A copy of the insurance policy and conditions are distributed to the patient upon enrollment into the study and the patient is advised to adhere to the conditions of the insurance policy to safeguard a valid patient insurance. Since 1.1.2020, the commuting accident insurance is included in the patient insurance

11.4. Confidentiality

The data obtained in the course of the trial will be treated according to the European General Data Protection Regulation (Datenschutz-Grundverordnung; DS-GVO) and the applicable local data protection regulations as well as the applicable regulations for the clinical trial.

Subjects have to be informed about data protection in the clinical trial and to consent in writing to collect and process their personalized data as well as to transfer their pseudonymized data. The information has to be transparent, precise, easily accessible and understandable and is written in clear and simple language. The written privacy policy must be approved by the responsible ethics committee.

In order to maintain patient privacy, all data capture records, study drug accountability records, study reports and communications will identify the patient by the assigned patient number. The PI determines which persons are authorized to view personal data, the Patient Identification Log is only accessible to authorized study team members. Restricted access rights to personal data (including pseudonymised data) are implemented to prevent unauthorized access to the data (both electronically and physically). Electronic systems and files are access-regulated, possibly password-protected. Documents and files are kept in lockable rooms, if necessary, cupboards with access control.

The patient name, initials and the full birth date should never be used in any correspondence with the Sponsor or on the Case Report Forms. The investigator will grant monitor(s) and auditor(s) and/or regulatory authorities direct access to the patient's original medical records for verification of data gathered on the data capture records and to audit the data collection process. Direct access includes examining, analyzing, and verifying any recorded data and reports that are important to the evaluation of the monitoring. The investigator is obliged to inform the patient that his/her trial-related records will be viewed without violating their confidentiality and that the collected information will only be made publicly available to the extent permitted by the applicable laws and regulations. All data will be stored either

paperbased or electronically in a pseudonymous manner and handled strictly confidential. The investigators are obliged to keep all study data and information confidential and to use those data only in context with the persons involved in the trial conduct. Study material or information developed in this trial must not be available to third parties, except for official representatives of the sponsor or regulatory authorities.

Data will be processed at the study site according to the written safety concept of this institution. Access to the data will be strictly limited to authorized persons. Loss of data is excluded due to extensive back-up procedures. All legal requirements concerning data protection and confidentiality will be respected. All authorized persons are sworn to secrecy.

In the case of withdrawal of consent the stored data collected to this time point will be stored and further used. Data not necessary any longer are deleted immediately.

Collected study data will be stored for at least 25 years after the end of the trial, if there are no other regulatory archiving periods. After archiving has expired, the data will be destructed in a data protection compliant manner.

When processing personal data, the following principles must be observed (pursuant to DS-GVO Article 5 “Principles relating to processing of personal data“):

Personal data shall be:

- processed lawfully, fairly and in a transparent manner in relation to the data subject
- collected for specified, explicit and legitimate purposes and not further processed in a manner that is incompatible with those purposes
- adequate, relevant and limited to what is necessary in relation to the purposes for which they are processed
- accurate and, where necessary, kept up to date
- kept in a form which permits identification of data subjects for no longer than is necessary for the purposes for which the personal data are processed
- processed in a manner that ensures appropriate security of the personal data, including protection against unauthorized or unlawful processing and against accidental loss, destruction or damage, using appropriate technical or organizational measures

Minimal criteria for Technical and organizational measures (TOMs) are:

Access control (measures to prevent unauthorized access)

- (Manual) locking system (access to office rooms)
- Access only for authorized persons
- Locked doors and cabinets in case of absence

Access control (measures to prevent unauthorized access to IT-systems)

- Access to IT systems is protected by an authorization concept
- Access to the PC is protected by a personal password and user name
- Password policies are in place
- Regular request to change the password
- Automatically or manually triggered screen lock when the PC is not in use
- Anti-virus software
- Firewall
- Remote access via VPN and double authentication
- Access control for external authorized persons (e.g. monitors, inspectors) to patient records

Access control (measures to prevent personal data from being read, copied, changed or removed without authorization)

- Data protected destruction of paper documents by using a document shredder or a qualified service provider
- Lockable cabinets in the offices or if available steel cabinets (fire and theft protection) for particularly sensitive (original) documents

Separation control (Measures to ensure that data collected for different purposes can be processed separately)

- Separation of productive and test environment for databases
- Definition of database rights

Pseudonymization / Transfer control

- Storage of the Subject ID Log in a separate and secure system (access only for the study team)
- Transfer of subject data only pseudonymized

Input control (Measures that ensure that it can be subsequently determined whether and by whom personal data has been entered, changed or removed in IT systems)

- Audit trail for GCP-compliant IT systems

- Login data only after appropriate training or authorization

Availability control (measures to ensure that personal data is protected against accidental destruction or loss)

- Smoke alarm system
- Fire extinguishers
- Physically separate server locations with an uninterruptible power supply
- Data security / backup via a central security system. A retrospective restoration of the data is possible.

Data protection management

- Data protection officer
- Quality management system with standard operating procedure(s)
- Employees are obliged to maintain data secrecy
- IT security officer
- Documented IT security measures

Incident Response Management

- Use of firewall and regular updates
- Use of spam filters and regular updates
- Process for reporting data breaches

11.5. Responsibility of the Investigator

The investigator should ensure that all persons assisting with the trial are adequately informed about the protocol, any amendments to the protocol, the trial treatments, and their trial-related duties and functions.
The investigator will maintain a list of subinvestigators and other appropriately qualified persons to whom he or she has delegated significant trial-related duties.

11.6. Registration of the Trial

Prior to the beginning of the clinical phase (First Patient In) the Sponsor / CI will register the trial in the EudraCT (AMG) /EUDAMED (MPG, MDR) as well as ClinicalTrials.gov Database.

11.7. Reporting obligations (Sponsor) according to the EU-CTR 536/2014

The Sponsor will notify each member state (MS) concerned via CTIS within 15 days of:

• the start of the clinical trial in relation to that MS

• the first visit of the first subject in relation to that MS

• the end of the recruitment of subjects for a clinical trial in that MS

• the end of a clinical trial in relation to that MS

• the end of a clinical trial in all MS concerned (end of the clinical trial in the last MS concerned)

• the end of a clinical trial in all MS concerned and in all third countries in which the clinical trial has been conducted

• a temporary halt of a clinical trial in all MS concerned for reasons not affecting the benefitrisk balance

• a temporarily halted clinical trial is resumed

In the case of early termination of the clinical trial for reasons not affecting the benefit-risk balance, the sponsor notifies each MS concerned through CTIS of the reasons for such action and, when appropriate, follow-up measures for the subjects.

The Sponsor will notify each member state (MS) concerned via CTIS, without undue delay but not later than 15 days of:

• the temporary halt or early termination of a clinical trial for reasons of a change of the benefit-risk balance (including the reasons for such action and specify follow-up measures)

• The restart of the clinical trial following a temporary halt shall be deemed to be a substantial modification subject to the authorisation procedure.

According to the German Drug Law (AMG) and the GCP Ordinance, eachmember state (MS) concerned will be informed of all suspected serious unexpected adverse reactions (SUSARs) via CTIS. Both institutions will be informed in case the risk/ benefit assessment did change or any others new and significant hazards for subjects' safety or welfare did occur. Furthermore, a report on all observed SAEs will be submitted once a year – Annual Safety Report.

11.8. Approval of Protocol and Subsequent Amendments

Before the start of the trial, the trial protocol, informed consent document, and any other appropriate documents will be submitted to the independent Ethics Committee (EC) as well as to the competent authority (PEI). A written favourable vote of the EC and an (implicit) approval by the competent higher federal authority as well as the notification of the local authorities (acc. to §67 AMG) are a prerequisite for initiation of this clinical trial. Before the first subject is enrolled in the trial, all ethical and legal requirements must be met. All planned substantial changes (see §10, (1) of German GCP-Regulation) will be submitted for approval to EC and the competent authority in writing as protocol amendments.

After transition every new version of a trial protocol, informed consent document, and any other appropriate documents will be submitted to the Member States concerned via CTIS. The notification of authorization of the Member State(s) concerned through CTIS is a prerequisite for continuation of this clinical trial. All planned substantial changes will be submitted for approval to the Member States concerned in writing as protocol amendments.

12. Publications

12.1. Reports

Within one year of the completion of the trial in all Member States concernced, the Sponsor will submit to the EU database a summary of the results of the clinical trial accompanied by a summary written in a manner that is understandable to laypersons.

12.2. Publication

The final results of this study will be presented at scientific meetings and published in a peer reviewed journal.
All publications based on data from this clinical trial are the responsibility of the principal coordinating investigator (sponsor). Authorships will reflect the contributions of each collaborating center.

13. Financing

This study is an investigator-initiated trial and financially supported by the Ministry of Science and Arts of the State of Baden-Württemberg within the funding call “Health First”. Translational studies will be covered by further research grants.

14. Appendix

14.1. Appendix I: LANO (Le Rhun et al., 2019)

Patient Identification	Reference Scan		Follow-up		Response Assessment[8]
Name/number:<br>Sex:<br>Date of birth:<br>Dates of MRI					
Relevant history		n.a.	Treatment since reference scan[7]:<br>Date of last lumbar puncture		n.a.
MRI findings	Present (1) or absent (0) or non-evaluable (NE)[5]	Individual dimensions (N1, N2, N3: X x Y mm) of 3 largest measurable nodules (measurable defined as > 5 x 5 mm (orthogonal diameters in 2 planes	Present (1) or absent (0) or non-evaluable (NE)	Individual dimensions (N1, N2, N3: X x Y mm) of 3 largest meas-urable nodules (measurable de-fined as > 5 x 5 mm (orthogonal diameters in 2 planes	Change from previous MRI
**Items related to assessment to leptomeningeal metastasis**					
**BRAIN**					
Nodules (subarachnoid or ventricular)					□ improved<br>□ no change<br>□ worse
Leptomeningeal linear enhancement[2]		n.a.[6]		n.a.	□ improved<br>□ no change<br>□ worse
Hydrocephalus[3]		n.a.		n.a.	□ improved<br>□ no change<br>□ worse
**SPINE**					
Nodules (subarachnoid)					
Leptomeningeal linear enhancement		n.a.		n.a.	

**Overall response assessment for LM (CR, PR, SD, PD, or NE)** Items *not* related to assessment of leptomeningeal metastasis[4]	
**BRAIN**	
Parenchymal (brain) metastases	□ CR[9]<br>□ PR<br>□ SD<br>□ PD
**Spine**	
Parenchymal (intramedullary) metastases	□ CR<br>□ PR<br>□ SD<br>□ PD

Abbreviations: CR = complete response, PD = partial response, SD = stable disease, PD = progressive disease.

Explanations:

[1] A **nodule** is a contrast-enhancing lesion that is defined as LM-related as opposed to parenchymal if there is direct contact (less than 2 mm distance) between the outer edge of the nodule and the leptomeninges on contrast-enhanced scans.

[2] **Leptomeningeal linear enhancement** may include cranial nerve or spinal nerve root, cerebellar folia, ventricular ependymal, or cerebral sulcal enhancement.

[3] Hydrocephalus is assessed by determining the Evans index calculated on T1-weighted axial MR images. It represents the ratio of the largest diameter at the maximal width of the frontal horns relative to the largest internal diameter of the cranium on the same slide (Brix et al. Eur J Radiol 2017;95:28–32).

[4] These items shall be documented as present or absent, but are not used for LM response assessment.

[5] NE refers to scans that cannot be assessed for poor quality or incomplete sequences.

[6] Not applicable.

[7] **Therapeutic options** for LM that should be noted here include any neurosurgical intervention, radiotherapy with information on the target of irradiation, systemic pharmacotherapy, and intrathecal pharmacotherapy. Assessing response treatment requires precise information on treatment delivered.

[8] **Progression** is diagnosed if there is at least one new measurable nodule, if at least one measurable nodule that does not reach 10 mm in its two largest perpendicular diameters, increases in the product of the largest perpendicular diameters by 50% or more, if at least one nodule of at least 10 mm diameter in its perpendicular diameters increases in the product of the largest perpendicular diameters by 25% or more, or if the largest ventricular diameter increases by at least 25%. De novo linear leptomeningeal contrast enhancement alone also qualifies for progression unless attributable to lumbar puncture. **Partial response** requires regression of the size of all measurable nodules by 50% or more, without an increase in ventricular size. **Complete response** requires resolution of all contrast-enhancing, LM-related measurable lesions, without an increase in ventricular size. All other situations are considered **stable disease**. LM without measurable nodules can only remain stable as its best response. Linear enhancement cannot be quantified and is thus only noted as absent or present, but not used for response assessment unless developing de novo or affecting the leptomeninges in anatomic regions not previously affected—then this constitutes **progressive disease**. Deterioration in any one item qualifying for progression will be sufficient to call progression.

[9] According to RANO imaging criteria for brain metastasis (Lin et al. Lancet Oncology 2015;16:e270-8).

Technical considerations

MRI scans should be performed on the same scanner or at least a device of identical field strength during Follow-up using the same imaging protocol at all timepoints during the Follow-up, gadolinium-based contrast agent should be injected ideally 10 min, but not less than 5 min before acquisition of T1-weighted sequences and the slice thickness should be 1 mm or less in the brain and 3 mm or less for the spinal cord, as the leptomeningeal enhancement may have complex aspects and is commonly linear (Le Rhun et al. Ann Oncol 2017;28:iv84-iv99).

Since lumbar punctures may induce leptomeningeal enhancement, the date(s) of the last CSF analysis performed before MRI acquisition should appear on the grid

14.2. Appendix II: Reference safety information for assessment of expectedness of serious adverse reactions (RSI)

The Reference Safety Information (RSI) is used for the assessment of the expectedness of all ‘suspected’ serious adverse reactions (SARs) that can occur in the clinical trial, for regulatory reporting purposes. The SARs are classified using Preferred Terms (PTs) according to the Medical Dictionary for Regulatory Activities (MedDRA).

This expectedness assessment of each ‘suspected’ SAR is required to determine expedited reporting of ‘suspected unexpected serious adverse reactions (SUSARs), and for the identification of SUSARs in the cumulative summary tabulation of ‘suspected’ SARs in the Development Safety Update Report (DSUR).

The information provided within this section does not present a comprehensive overview of the safety profile of the IMP(s).

This cumulative safety data regarding the used IMPs from across the clinical development will be regularly reviewed and revised during the annual update during DSUR.

SOC	SARs	Number of subjects exposed (n) = XX		
		All SARs	Occurrence of fatal SARs	Occurrence of life-threatening SARs
		n* (%)	n* (%)	n* (%)
t.b.d.	t.b.d.	t.b.d.	t.b.d.	t.b.d.

Table 5: Serious Adverse Reactions (SAR) for the IMP considered expected for safety reporting purposes (n = number of subjects who have experienced the SAR; SOC = system organ class; t.b.d. = to be determined.)

Expectable serious side effects are not known, yet. The section will be updated as soon as data become available.

14.3. Appendix III: NCCN Guidelines (NCCN, 2021a)

For personal use only. Not approved for distribution. Copyright © 2021 National Comprehensive Cancer Network, Inc., All Rights Reserved.

NCCN National Comprehensive Cancer Network®

NCCN Guidelines Version 3.2021
Management of Immune Checkpoint Inhibitor-Related Toxicities

NCCN Guidelines Index
Table of Contents
Discussion

NERVOUS SYSTEM ADVERSE EVENT(S)	ASSESSMENT/GRADING		MANAGEMENT[d]
Guillain-Barré syndrome (GBS)[j] →	• Inpatient care with access to ICU-level monitoring<br>• Neurology consultation<br>• MRI of spine with or without contrast (rule out compressive lesion)<br>• Lumbar puncture[k]<br>• Serum ganglioside antibody tests for GBS variants (GQ1b for Miller Fisher variant associated with ataxia and ophthalmoplegia)<br>• Pulmonary function testing (NIF/VC)	→ Moderate (G2)[l] or Severe (G3–4)[m] →	• Permanently discontinue immunotherapy[e]<br>• Inpatient care with capability of rapid transfer to ICU-level monitoring<br>• Start IVIG[g] or plasmapheresis in addition to pulse-dose methylprednisolone 1 gram daily for 5 days[n] then taper over 4 weeks<br>• Frequent neurologic evaluation and pulmonary function monitoring<br>• Monitor for concurrent autonomic dysfunction<br>• Gabapentin, pregabalin, or duloxetine for pain

d See Principles of Immunosuppression (IMMUNO-A).
e See Principles of Immunotherapy Rechallenge (IMMUNO-C).
g Total dosing should be 2 g/kg, administered in divided doses per package insert.
j Progressive, most often symmetrical muscle weakness with absent or reduced deep tendon reflexes. May involve extremities, facial, respiratory, and bulbar and oculomotor nerves. May have dysregulation of autonomic nerves. Often starts with pain in lower back and thighs.
k Cerebrospinal fluid (CSF) typically has elevated protein and often elevated white blood cell (WBC) count; even though this is not typically seen in classical GBS, cytology should be sent with any CSF sample.
l Some interference with ADLs, symptoms concerning to patient.
m Limiting self-care and aids warranted, weakness limiting walking, any dysphagia, facial weakness, respiratory muscle weakness, or rapidly progressive symptoms.
n Steroids are not usually recommended for idiopathic GBS; however, in immunotherapy-related forms, a trial is reasonable in addition to IVIG or plasmapheresis.

Note: All recommendations are category 2A unless otherwise indicated.
Clinical Trials: NCCN believes that the best management of any patient with cancer is in a clinical trial. Participation in clinical trials is especially encouraged.

Version 3.2021, 05/14/21 © 2021 National Comprehensive Cancer Network® (NCCN®), All rights reserved. NCCN Guidelines® and this illustration may not be reproduced in any form without the express written permission of NCCN.

ICI_NEURO-2

. For personal use only. Not approved for distribution. Copyright © 2021 National Comprehensive Cancer Network, Inc., All Rights Reserved.

NCCN National Comprehensive Cancer Network®

NCCN Guidelines Version 3.2021
Management of Immune Checkpoint Inhibitor-Related Toxicities

NCCN Guidelines Index
Table of Contents
Discussion

NERVOUS SYSTEM ADVERSE EVENT(S)	ASSESSMENT/GRADING		MANAGEMENT[d]
Myasthenia gravis[a] →	• Acetylcholine receptor (AChR) antibodies and anti–muscle-specific tyrosine kinase antibodies in blood (not needed for diagnosis)<br>• Pulmonary function assessment with negative inspiratory force (NIF) and vital capacity (VC)<br>• ESR, CRP, creatinine phosphokinase (CPK), aldolase, and anti-striational antibodies for superimposed myositis<br>• If respiratory insufficiency or elevated CPK, perform cardiac exam, ECG, troponin, and transthoracic echocardiogram (TTE) for possible concomitant myocarditis<br>• Electromyography (EMG) with repetitive stimulation and nerve conduction study (NCS)<br>• Neurology consultation<br>• Consider MRI brain and/or spine depending on symptoms to rule out CNS involvement by disease	Moderate (G2)[b] →	• Permanently discontinue immunotherapy[e]<br>• Inpatient care<br>• Pyridostigmine 30 mg TID and gradually increase to maximum of 120 mg orally four times a day as tolerated and based on symptoms<br>• Consider low-dose oral prednisone 20 mg daily.[f] Increase by 5 mg every 3–5 days to a target dose of 1 mg/kg/day but not more than 100 mg daily (steroid taper based on symptom improvement)
		Severe (G3–4)[c] →	• Permanently discontinue immunotherapy[e]<br>• Inpatient care (may need intensive care unit [ICU]-level monitoring)<br>• Methylprednisolone 1–2 mg/kg/day[f] (steroid taper based on symptom improvement)<br>• Initiate plasmapheresis or IVIG[g]<br>▸ Consider adding rituximab[h] (375 mg/m² weekly for 4 treatments or 500 mg/m² every 2 weeks for 2 doses) if refractory to plasmapheresis or IVIG<br>• Frequent pulmonary function assessment<br>• Daily neurologic evaluation<br>• Avoid medications that can worsen myasthenia[f,i]

[a] Progressive or fluctuating muscle weakness, generally proximal to distal. May have bulbar involvement (ie, ptosis, extraocular movement abnormalities resulting in double vision, dysphagia, facial muscle weakness) and/or respiratory muscle weakness. May occur with myositis and myocarditis. Respiratory symptoms may require evaluation to rule out pneumonitis. Miller Fisher variant of Guillain-Barré syndrome (GBS) has overlapping symptoms (ophthalmoplegia and ascending weakness).

[b] Some symptoms interfering with ADLs. Myasthenia Gravis Foundation of America (MGFA) severity class I (ocular symptoms and findings only) and MGFA severity class II (mild generalized weakness).

[c] Limiting self-care and aids warranted, weakness limiting walking, any dysphagia, facial weakness, respiratory muscle weakness, or rapidly progressive symptoms or MGFA severity class III–IV moderate to severe generalized weakness to myasthenic crisis.

[d] See Principles of Immunosuppression (IMMUNO-A).

[e] See Principles of Immunotherapy Rechallenge (IMMUNO-C).

[f] High-dose steroids (≥2 mg/kg/day) may exacerbate symptoms.

[g] Total dosing should be 2 g/kg, administered in divided doses per package insert.

[h] An FDA-approved biosimilar is an appropriate substitute for rituximab.

[i] Beta-blockers, ciprofloxacin, and IV magnesium.

Note: All recommendations are category 2A unless otherwise indicated.
Clinical Trials: NCCN believes that the best management of any patient with cancer is in a clinical trial. Participation in clinical trials is especially encouraged.

ICI_NEURO-1

Version 3.2021 , 05/14/21 © 2021 National Comprehensive Cancer Network® (NCCN®), All rights reserved. NCCN Guidelines® and this illustration may not be reproduced in any form without the express written permission of NCCN.

For personal use only. Not approved for distribution. Copyright © 2021 National Comprehensive Cancer Network, Inc., All Rights Reserved.

NCCN National Comprehensive Cancer Network®

NCCN Guidelines Version 3.2021
Management of Immune Checkpoint Inhibitor-Related Toxicities

NCCN Guidelines Index
Table of Contents
Discussion

NERVOUS SYSTEM ADVERSE EVENT(S)	GRADING		MANAGEMENT[d]
Peripheral neuropathy[o,p]	Mild (G1)[q]	• Evaluate for other causes of neuropathy such as: medication, infection, metabolic/endocrine disorders, environmental exposures, vascular or autoimmune disease, trauma, etc.<br>• Consider neuraxial imaging as per neurology	• Consider holding immunotherapy[e,t]<br>• Monitor symptoms for a week[u]
	Moderate (G2)[r]	• Evaluate for other causes of neuropathy such as: medication, infection, metabolic/endocrine disorders, environmental exposures, vascular or autoimmune disease, trauma, etc.<br>• Neuraxial imaging as per neurology<br>• Consider EMG/NCS<br>• Consider neurology consultation	• Hold immunotherapy[e]<br>• Initial observation or initiate prednisone 0.5–1 mg/kg orally (if progressing from mild)[v]<br>• If progression, initiate methylprednisolone 2–4 mg/kg/day[v] and see Guillain-Barré Syndrome (ICI_NEURO-2)<br>• Gabapentin, pregabalin, or duloxetine for pain
	Severe (G3–4)[s]	See Guillain-Barré Syndrome (ICI_NEURO-2)	

[d] See Principles of Immunosuppression (IMMUNO-A).
[e] See Principles of Immunotherapy Rechallenge (IMMUNO-C).
[o] Can present as asymmetric or symmetric sensory-motor deficit. Sensory deficit may be painful or painless paresthesias or potentially life-threatening autonomic (eg, myenteric plexus) dysfunction. Hypo- or areflexia. Isolated sensory deficit or sensory plus lower motor neuron deficit.
[p] GI tract paresis due to myenteric neuritis is a rare toxicity associated with ICI therapy. The presentation may be fulminant with profound ileus. Early institution of high-dose steroids in concert with multidisciplinary management is recommended.
[q] No interference with function and symptoms not concerning to patient. Note: any cranial nerve problem should be managed as moderate.
[r] Some interference with ADLs, symptoms concerning to patient (ie, pain but no weakness or gait limitation).
[s] Limiting self-care and aids warranted, weakness limiting walking or respiratory problems (ie, leg weakness, foot drop, rapidly ascending sensory changes). Severe peripheral neuropathy is not necessarily GBS but the management can be similar.
[t] There is a low threshold to hold ICIs in mild cases of peripheral neuropathy.
[u] Specifically monitor for new interference with iADLs from either pain or weakness, gait difficulty, ataxia, or autonomic changes.
[v] Treat until symptoms improve to Grade ≤1 then taper over 4–6 weeks.

Note: All recommendations are category 2A unless otherwise indicated.
Clinical Trials: NCCN believes that the best management of any patient with cancer is in a clinical trial. Participation in clinical trials is especially encouraged.

ICI_NEURO-3

Version 3.2021 , 05/14/21 © 2021 National Comprehensive Cancer Network® (NCCN®), All rights reserved. NCCN Guidelines® and this illustration may not be reproduced in any form without the express written permission of NCCN.

For personal use only. Not approved for distribution. Copyright © 2021 National Comprehensive Cancer Network, Inc., All Rights Reserved.

NCCN National Comprehensive Cancer Network®

NCCN Guidelines Version 3.2021
Management of Immune Checkpoint Inhibitor-Related Toxicities

NCCN Guidelines Index
Table of Contents
Discussion

NERVOUS SYSTEM ADVERSE EVENT(S)	ASSESSMENT	MANAGEMENT[d]
Aseptic meningitis[w,x] →	• MRI brain with and without contrast + pituitary protocol<br>• Consider lumbar puncture[z] if feasible<br>• Consider neurology consultation	→ • Hold immunotherapy[e] if mild/moderate<br>• Permanently discontinue immunotherapy if severe<br>• Inpatient care (G3–4[cc])<br>• Consider IV acyclovir[ee] until polymerase chain reaction (PCR) results obtained<br>• Rule out bacterial and viral infection, then may closely monitor off steroids or consider prednisone 0.5–1 mg/kg/day or methylprednisolone 1–2 mg/kg/day if moderate/severe symptoms[dd]
Encephalitis[x,y] →	• Neurology consultation<br>• MRI brain with and without contrast[aa]<br>• Lumbar puncture[bb]<br>• Electroencephalogram (EEG) to evaluate for subclinical seizures<br>• CMP, CBC, ESR, CRP, antineutrophil cytoplasmic antibody (ANCA) (if vasculitic process suspected), thyroid panel including TPO and thyroglobulin<br>• Autoimmune encephalopathy and paraneoplastic panel in CSF and serum	→ • Hold immunotherapy[e] if mild<br>• Permanently discontinue immunotherapy if moderate/severe<br>• Inpatient care (G3–4[cc])<br>• Consider IV acyclovir[ee] until PCR results obtained<br>• Trial of methylprednisolone 1–2 mg/kg/day[dd]<br>• If severe or progressing symptoms or oligoclonal bands present, consider pulse steroids methylprednisolone 1 g IV daily for 3–5 days plus IVIG[g] or plasmapheresis<br>• If positive for autoimmune encephalopathy antibody or limited or no improvement after 7–14 days, consider rituximab[h]

[d] See Principles of Immunosuppression (IMMUNO-A).
[e] See Principles of Immunotherapy Rechallenge (IMMUNO-C).
[g] Total dosing should be 2 g/kg, administered in divided doses per package insert.
[h] An FDA-approved biosimilar is an appropriate substitute for rituximab.
[w] May present with headache, photophobia, and neck stiffness, often afebrile but may be febrile. There may be nausea/vomiting. Mental status should be normal (distinguishes from encephalitis).
[x] Exclude infectious causes, especially viral (ie, HSV).
[y] Confusion, altered behavior, headaches, seizures, short-term memory loss, depressed level of consciousness, focal weakness, and speech abnormality.
[z] Measure opening pressure and check cell count, protein glucose, Gram stain, culture, PCR for HSV, and other viral PCRs depending on suspicion and cytology. May see elevated WBC with normal glucose, normal culture, Gram stain, and elevated protein. May see reactive lymphocytes or histiocytes on cytology.
[aa] May reveal T2/FLAIR changes typical of what is seen in autoimmune encephalopathies or limbic encephalitis or may be normal.
[bb] Check cell count, protein glucose, Gram stain, culture, PCR for HSV, and other viral PCRs depending on suspicion, cytology, oligoclonal bands, and autoimmune encephalopathy panel. May see elevated WBC with lymphocytic predominance and/or elevated protein.
[cc] Limiting self-care and aids warranted.
[dd] Taper steroids over 4 weeks once symptoms resolve.
[ee] 10 mg/kg IV every 8 hours.

Note: All recommendations are category 2A unless otherwise indicated.
Clinical Trials: NCCN believes that the best management of any patient with cancer is in a clinical trial. Participation in clinical trials is especially encouraged.

ICI_NEURO-4

Version 3.2021 , 05/14/21 © 2021 National Comprehensive Cancer Network® (NCCN®), All rights reserved. NCCN Guidelines® and this illustration may not be reproduced in any form without the express written permission of NCCN.

For personal use only. Not approved for distribution. Copyright © 2021 National Comprehensive Cancer Network, Inc., All Rights Reserved.

National Comprehensive Cancer Network®

NCCN Guidelines Version 3.2021
Management of Immune Checkpoint Inhibitor-Related Toxicities

NCCN Guidelines Index
Table of Contents
Discussion

NERVOUS SYSTEM ADVERSE EVENT(S)	ASSESSMENT	MANAGEMENT[d]
Transverse myelitis[ff] →	• Neurology consultation<br>• MRI of spine and brain<br>• Lumbar puncture[gg]<br>• B_{12}, HIV, rapid plasma reagin (RPR), ANA, anti-Ro/La antibodies, TSH, aquaporin-4 IgG, paraneoplastic panel for anti-Hu and anti-CRMP5/CV2<br>• Evaluation for constipation and urinary retention with bladder scan →	• Permanently discontinue immunotherapy[e]<br>• Inpatient care<br>• Methylprednisolone pulse dosing 1 g/day for 3–5 days<br>• Strongly consider IVIG[g] or plasmapheresis

[d] See Principles of Immunosuppression (IMMUNO-A).
[e] See Principles of Immunotherapy Rechallenge (IMMUNO-C).
[g] Total dosing should be 2 g/kg, administered in divided doses per package insert.
[ff] Acute or subacute weakness or sensory changes bilaterally, often with increased deep tendon reflexes.
[gg] Cell count, protein, glucose, oligoclonal bands, viral PCRs, cytology, and onconeural antibodies.

Note: All recommendations are category 2A unless otherwise indicated.
Clinical Trials: NCCN believes that the best management of any patient with cancer is in a clinical trial. Participation in clinical trials is especially encouraged.

ICI_NEURO-5

Version 3.2021 , 05/14/21 © 2021 National Comprehensive Cancer Network® (NCCN®), All rights reserved. NCCN Guidelines® and this illustration may not be reproduced in any form without the express written permission of NCCN.

For personal use only. Not approved for distribution. Copyright © 2021 National Comprehensive Cancer Network, Inc., All Rights Reserved.

NCCN National Comprehensive Cancer Network®

NCCN Guidelines Version 3.2021
Management of Immune Checkpoint Inhibitor-Related Toxicities

NCCN Guidelines Index
Table of Contents
Discussion

PRINCIPLES OF IMMUNOTHERAPY RECHALLENGE

General Principles

- **Exercise caution when considering resumption of immunotherapy after significant irAEs. Close follow-up should be performed when resuming immunotherapy to monitor for recurrent symptoms.**
  - **If re-challenged and toxicity returns, permanently discontinue class of immunotherapy.**
  - **Assess patient's tumor status prior to rechallenge. If an objective response (complete or partial) to ICI therapy was achieved, resumption of immunotherapy may not be advisable due to risk of toxicity recurrence. Discuss the risks/benefits of restarting immunotherapy with the patient.**
- **Permanent discontinuation of a given class of immunotherapy is typically warranted in the setting of severe irAEs induced by that class of immunotherapy and may be warranted in the setting of moderate irAEs. For example, if a patient experiences grade 3 or 4 toxicity from an ipilimumab-containing regimen, consideration may be given to later therapy with a PD-1 or PD-L1 monotherapy after resolution of the earlier toxicity.**
- **With some exceptions, resumption of immunotherapy following grade 2 irAEs can be considered upon resolution to ≤ grade 1.**
- **Consult with organ-specific specialists prior to resumption of immunotherapy as appropriate following an immunotherapy hold due to irAEs.**

Organ-Specific Considerations for Immunotherapy Rechallenge After a Hold

**Cardio-vascular**	• Grade 1 myocarditis: Consider resuming upon resolution of symptoms.<br>• Permanent discontinuation is warranted in the setting of grade 2–4 myocarditis.
**Endocrine**	• Thyroid: No discontinuation required for hypothyroidism. For symptomatic hyperthyroidism resembling Graves-like disease, consider holding immunotherapy and resuming after workup is complete and there is evidence for improvement in symptoms and TFTs.<br>• Hypophysitis manifested by deficiency of ACTH, TSH, and/or gonad-stimulating hormones, but without symptomatic pituitary swelling: Immunotherapy may continue while replacement endocrine therapy is regulated.<br>• Hypophysitis accompanied by symptoms of pituitary swelling (eg, headache, vision disturbance, and/or neurologic dysfunction): Hold immunotherapy until resolution of symptoms after steroid therapy; consider resumption of immunotherapy after symptoms related to mass effect are resolved.<br>• T1DM with DKA: Consider resuming once DKA has been corrected and glucose level has stabilized.<br>• Primary adrenal insufficiency: After appropriate replacement endocrine therapy is instituted, immunotherapy may continue.
**Eye**	• Grade 2-4 irAE: Hold immunotherapy per guideline; consider resumption of immunotherapy in consultation with ophthalmology upon resolution to ≤grade 1.
**GI**	• PD-1/PD-L1 agents: After grade 2–3 colitis, consider resumption of immunotherapy after symptoms have resolved to ≤ grade 1. In rare circumstances in which the patient cannot completely taper off steroids and symptoms are unresolved, immunotherapy may be resumed while patient is still on ≤10 mg prednisone equivalent daily. Consider concurrent vedolizumab upon resumption of PD-1/PD-L1.<br>• CTLA-4 agents: Discontinue if irAE is serious or life-threatening. Do not make up doses missed due to irAE and/or required steroid treatment.

Note: All recommendations are category 2A unless otherwise indicated.
Clinical Trials: NCCN believes that the best management of any patient with cancer is in a clinical trial. Participation in clinical trials is especially encouraged.

Continued

Version 3.2021 , 05/14/21 © 2021 National Comprehensive Cancer Network® (NCCN®), All rights reserved. NCCN Guidelines® and this illustration may not be reproduced in any form without the express written permission of NCCN.

For personal use only. Not approved for distribution. Copyright © 2021 National Comprehensive Cancer Network, Inc., All Rights Reserved.

NCCN National Comprehensive Cancer Network®

NCCN Guidelines Version 3.2021
Management of Immune Checkpoint Inhibitor-Related Toxicities

NCCN Guidelines Index
Table of Contents
Discussion

PRINCIPLES OF IMMUNOTHERAPY RECHALLENGE

Organ-Specific Considerations for Immunotherapy Rechallenge After a Hold

**Kidney**	• Hold immunotherapy per guidelines; upon resolution to ≤ grade 1, consider resuming concomitant with or without steroid if creatinine is stable.<br>• After restarting immunotherapy, monitor creatinine every 2–3 weeks or more frequently as clinically indicated. If creatinine remains stable, consider longer durations between creatinine checks.<br>• Consider permanent discontinuation in the setting of severe (grade 3–4) proteinuria. See Discussion.<br>• For resolved G2 and/or G3 renal irAE, may consider re-challenge if clinically indicated, at least after ≥2 months of holding ICI therapy.
**Liver**	• Transaminitis without elevated bilirubin: Following a grade 2 irAE, consider resumption of immunotherapy after ALT/AST return to baseline and steroids, if used, have been tapered to ≤10 mg prednisone equivalent daily.<br>• For grade 3 hepatitis, if on CTLA-4 combined with PD-1/PD-L1, restart with just PD-1/PD-L1 inhibitor.<br>• Permanent discontinuation is warranted in the setting of severe or life-threatening (grade 4) hepatitis.
**Lung**	• Progressive grade 1 pneumonitis requiring a hold: Consider resuming upon radiographic evidence of improvement.<br>• Grade 2: Resume once pneumonitis has resolved to ≤ grade 1 and patient is off steroids. Resume once pneumonitis has resolved to ≤ grade 1 and patient is on a corticosteroid dose of ≤10 mg/day of prednisone.<br>• Permanent discontinuation is warranted in the setting of severe (grade 3–4) pneumonitis.
**Musculo-skeletal**	• Inflammatory arthritis (moderate to severe irAE requiring hold): Resume upon stabilization, or adequate management of symptoms. Permanent discontinuation may be warranted for severe inflammatory arthritis that significantly impairs ADLs and quality of life.
**Nervous System**	• Myasthenia gravis: Permanently discontinue immunotherapy after grade 2–4 AE.<br>• GBS: Permanently discontinue immunotherapy for any grade GBS.<br>• Peripheral neuropathy: Following hold for grade 1–2 AE, consider resuming if symptoms resolve to ≤ grade 1 or if patient has well-controlled isolated painful sensory neuropathy.<br>• Aseptic meningitis: Consider resuming following mild to moderate AE if symptoms resolve to grade 0.<br>• Encephalitis: Permanent discontinuation is warranted in the setting of moderate to severe encephalitis (grade 2–4).<br>• Transverse myelitis: Discontinuation of immunotherapy following any-grade transverse myelitis.
**Pancreas**	• Symptomatic grade ≤2 pancreatitis: Consider resumption of immunotherapy if no clinical/radiologic evidence of pancreatitis ± improvement in amylase/lipase. Consider consultation with relevant pancreatic specialist regarding resumption.<br>• Permanent discontinuation is warranted for severe (grade 3–4) pancreatitis.
**Skin**	• Maculopapular rash and/or pruritus: Consider resuming after symptoms have resolved to ≤ grade 1 (ie, once skin condition is mild/localized with only topical intervention indicated).<br>• Permanent discontinuation of immunotherapy in the setting of severe or life-threatening bullous disease (grade 3–4), including all cases of SJS and TEN.

Note: All recommendations are category 2A unless otherwise indicated.
Clinical Trials: NCCN believes that the best management of any patient with cancer is in a clinical trial. Participation in clinical trials is especially encouraged.

IMMUNO-C 2 OF 2

Version 3.2021 , 05/14/21 © 2021 National Comprehensive Cancer Network® (NCCN®). All rights reserved. NCCN Guidelines® and this illustration may not be reproduced in any form without the express written permission of NCCN.

15. Literature

Chamberlain, M., Soffietti, R., Raizer, J., Rudà, R., Brandsma, D., Boogerd, W., . . . Jaeckle, K. A. (2014). Leptomeningeal metastasis: a Response Assessment in Neuro-Oncology critical review of endpoints and response criteria of published randomized clinical trials. *Neuro Oncol, 16*(9), 1176-1185. doi:10.1093/neuonc/nou089

Chamberlain, M. C. (1997). Carcinomatous meningitis. *Arch Neurol, 54*(1), 16-17. doi:10.1001/archneur.1997.00550130008003

Chen, G., Kim, Y. H., Li, H., Luo, H., Liu, D. L., Zhang, Z. J., . . . Ji, R. R. (2017). PD-L1 inhibits acute and chronic pain by suppressing nociceptive neuron activity via PD-1. *Nat Neurosci, 20*(7), 917-926. doi:10.1038/nn.4571

Clarke, J. L., Perez, H. R., Jacks, L. M., Panageas, K. S., & Deangelis, L. M. (2010). Leptomeningeal metastases in the MRI era. *Neurology, 74*(18), 1449-1454. doi:10.1212/WNL.0b013e3181dc1a69

Dierks, F., Pietsch, E., & Dunst, J. (2020). Pembrolizumab in der neoadjuvanten Behandlung des frühen triple-negativen Mammakarzinoms. *Strahlentherapie und Onkologie, 196*(9), 841-843. Retrieved from https://doi.org/10.1007/s00066-020-01641-9. doi:10.1007/s00066-020-01641-9

Glantz, M. J., Jaeckle, K. A., Chamberlain, M. C., Phuphanich, S., Recht, L., Swinnen, L. J., . . . Howell, S. B. (1999). A randomized controlled trial comparing intrathecal sustained-release cytarabine (DepoCyt) to intrathecal methotrexate in patients with neoplastic meningitis from solid tumors. *Clin Cancer Res, 5*(11), 3394-3402.

Glantz, M. J., Van Horn, A., Fisher, R., & Chamberlain, M. C. (2010). Route of intracerebrospinal fluid chemotherapy administration and efficacy of therapy in neoplastic meningitis. *Cancer, 116*(8), 1947-1952. doi:10.1002/cncr.24921

Glitza, I. C., Phillips, S., Brown, C., Haymaker, C. L., Bassett, R. L., Lee, J. J., . . . Davies, M. A. (2020). Single-center phase I/Ib study of concurrent intrathecal (IT) and intravenous (IV) nivolumab (N) for metastatic melanoma (MM) patients (pts) with leptomeningeal disease (LMD). *Journal of Clinical Oncology, 38*(15_suppl), 10008-10008. Retrieved from https://ascopubs.org/doi/abs/10.1200/JCO.2020.38.15_suppl.10008. doi:10.1200/JCO.2020.38.15_suppl.10008

Herrlinger, U., Förschler, H., Küker, W., Meyermann, R., Bamberg, M., Dichgans, J., & Weller, M. (2004). Leptomeningeal metastasis: survival and prognostic factors in 155 patients. *J Neurol Sci, 223*(2), 167-178. doi:10.1016/j.jns.2004.05.008

Hyun, J. W., Jeong, I. H., Joung, A., Cho, H. J., Kim, S. H., & Kim, H. J. (2016). Leptomeningeal metastasis: Clinical experience of 519 cases. *Eur J Cancer, 56*, 107-114. doi:10.1016/j.ejca.2015.12.021

Kesari, S., & Batchelor, T. T. (2003). Leptomeningeal metastases. *Neurol Clin, 21*(1), 25-66. doi:10.1016/s0733-8619(02)00032-4

Kim, J. Y., Kronbichler, A., Eisenhut, M., Hong, S. H., van der Vliet, H. J., Kang, J., . . . Gamerith, G. (2019). Tumor Mutational Burden and Efficacy of Immune Checkpoint Inhibitors: A Systematic Review and Meta-Analysis. *Cancers, 11*(11), 1798. Retrieved from https://pubmed.ncbi.nlm.nih.gov/31731749

https://www.ncbi.nlm.nih.gov/pmc/articles/PMC6895916/. doi:10.3390/cancers11111798

Le Rhun, E., Devos, P., Boulanger, T., Smits, M., Brandsma, D., Rudà, R., . . . Weller, M. (2019). The RANO Leptomeningeal Metastasis Group proposal to assess response to treatment: lack of feasibility and clinical utility and a revised proposal. *Neuro Oncol, 21*(5), 648-658. doi:10.1093/neuonc/noz024

Le Rhun, E., Taillibert, S., & Chamberlain, M. C. (2013). Carcinomatous meningitis: Leptomeningeal metastases in solid tumors. *Surg Neurol Int, 4*(Suppl 4), S265-288. doi:10.4103/2152-7806.111304

Le Rhun, E., Weller, M., Brandsma, D., Van den Bent, M., de Azambuja, E., Henriksson, R., . . . Preusser, M. (2017). EANO-ESMO Clinical Practice Guidelines for diagnosis, treatment and follow-up of patients with leptomeningeal metastasis from solid tumours. *Ann Oncol, 28*(suppl_4), iv84-iv99. doi:10.1093/annonc/mdx221

Lin, N. U., Lee, E. Q., Aoyama, H., Barani, I. J., Barboriak, D. P., Baumert, B. G., . . . Wen, P. Y. (2015). Response assessment criteria for brain metastases: proposal from the RANO group. *Lancet Oncol, 16*(6), e270-278. doi:10.1016/s1470-2045(15)70057-4

Loi, S., Giobbe-Hurder, A., Gombos, A., Bachelot, T., Hui, R., Curigliano, G., . . . Andre, F. (2018). Abstract GS2-06: Phase Ib/II study evaluating safety and efficacy of pembrolizumab and trastuzumab in patients with trastuzumab-resistant HER2-positive metastatic breast cancer: Results from the PANACEA (IBCSG 45-13/BIG 4-13/KEYNOTE-014) study. *Cancer Research, 78*(4 Supplement), GS2-06-GS02-06. doi:10.1158/1538-7445.Sabcs17-gs2-06

Mack, F., Baumert, B. G., Schäfer, N., Hattingen, E., Scheffler, B., Herrlinger, U., & Glas, M. (2016). Therapy of leptomeningeal metastasis in solid tumors. *Cancer Treat Rev, 43*, 83-91. doi:10.1016/j.ctrv.2015.12.004

Mehnert, A., Müller, D., Lehmann, C., & Koch, U. (2006). Die deutsche Version des NCCN Distress-Thermometers. *Zeitschrift für Psychiatrie, Psychologie und Psychotherapie, 54*(3), 213-223. Retrieved from https://econtent.hogrefe.com/doi/abs/10.1024/1661-4747.54.3.213. doi:10.1024/1661-4747.54.3.213

Nayak, L., DeAngelis, L. M., Brandes, A. A., Peereboom, D. M., Galanis, E., Lin, N. U., . . . Reardon, D. A. (2017). The Neurologic Assessment in Neuro-Oncology (NANO) scale: a tool to assess neurologic function for integration into the Response Assessment in Neuro-Oncology (RANO) criteria. *Neuro Oncol, 19*(5), 625-635. doi:10.1093/neuonc/nox029

Nayar, G., Ejikeme, T., Chongsathidkiet, P., Elsamadicy, A. A., Blackwell, K. L., Clarke, J. M., . . . Fecci, P. E. (2017). Leptomeningeal disease: current diagnostic and therapeutic strategies. *Oncotarget, 8*(42), 73312-73328. doi:10.18632/oncotarget.20272

NCCN. (2021a). NCCN Guidelines on Management of Checkpoint Inhibitor Releated Toxicities. 3.

NCCN. (2021b). NCCN Guidelines Version Central Nervous System Cancers. 1.

Pan, P. C., & Haggiagi, A. (2019). Neurologic Immune-Related Adverse Events Associated with Immune Checkpoint Inhibition. *Curr Oncol Rep, 21*(12), 108. doi:10.1007/s11912-019-0859-2

Thomas, K. H., & Ramirez, R. A. (2017). Leptomeningeal Disease and the Evolving Role of Molecular Targeted Therapy and Immunotherapy. *Ochsner J, 17*(4), 362-378.

Wang, Z., Jiang, C., He, Q., Matsuda, M., Han, Q., Wang, K., . . . Ji, R. R. (2020). Anti-PD-1 treatment impairs opioid antinociception in rodents and nonhuman primates. *Sci Transl Med, 12*(531). doi:10.1126/scitranslmed.aaw6471
